# Association between human blood metabolome and the risk of breast cancer

**DOI:** 10.1186/s13058-023-01609-4

**Published:** 2023-01-24

**Authors:** Yu Wang, Fanghua Liu, Lulu Sun, Yiming Jia, Pinni Yang, Daoxia Guo, Mengyao Shi, Aili Wang, Guo-Chong Chen, Yonghong Zhang, Zhengbao Zhu

**Affiliations:** 1 Department of Epidemiology, School of Public Health and Jiangsu Key Laboratory of Preventive and Translational Medicine for Geriatric Diseases, Suzhou Medical College of Soochow University, 199 Renai Road, Industrial Park District, Suzhou, 215123 Jiangsu Province China; 2 School of Nursing, Suzhou Medical College of Soochow University, Suzhou, China; 3 Department of Nutrition and Food Hygiene, School of Public Health, Medical College of Soochow University, Suzhou, China

**Keywords:** Breast cancer, Metabolites, Mendelian randomization

## Abstract

**Background:**

Breast cancer is the most common cancer among women with limited treatment options. To identify promising drug targets for breast cancer, we conducted a systematical Mendelian randomization (MR) study to screen blood metabolome for potential causal mediators of breast cancer and further predict target-mediated side effects.

**Methods:**

We selected 112 unique blood metabolites from 3 large-scale European ancestry-based genome-wide association studies (GWASs) with a total of 147,827 participants. Breast cancer data were obtained from a GWAS in the Breast Cancer Association Consortium (BCAC), involving 122,977 cases and 105,974 controls of European ancestry. We conducted MR analyses to systematically assess the associations of blood metabolites with breast cancer, and a phenome-wide MR analysis was further applied to ascertain the potential on-target side effects of metabolite interventions.

**Results:**

Two blood metabolites were identified as the potential causal mediators for breast cancer, including high-density lipoprotein cholesterol (HDL-C) (odds ratio [OR], 1.09; 95% confidence interval [CI], 1.06–1.12; *P* = 9.67 × 10^−10^) and acetate (OR, 1.24; 95% CI, 1.13–1.37;* P* = 1.35 × 10^−5^). In the phenome-wide MR analysis, lowering HDL-C might have deleterious effects on the risk of the circulatory system and foreign body injury, while lowering acetate had deleterious effects on mental disorders disease.

**Conclusions:**

The present systematic MR analysis revealed that HDL-C and acetate may be the causal mediators in the risk of developing breast cancer. Side-effect profiles were characterized to help inform drug target prioritization for breast cancer prevention. HDL-C and acetate might be promising drug targets for preventing breast cancer, but they should be applied under weighting advantages and disadvantages.

**Supplementary Information:**

The online version contains supplementary material available at 10.1186/s13058-023-01609-4.

## Introduction

Breast cancer is the most common cancer among women, which is the leading cause of cancer death in females [1, 2]. Over the past couple of decades, breast cancer incidence rates have increased continuously [3]. The American Cancer Society showed that breast cancer accounted for 30% of the projected cancer incidence among women in 2021 [4]. However, current treatments for breast cancer were quietly limited (e.g., surgery and radiation therapy) with a high rate of adverse side effects [5]. In addition, previous epidemiological studies had investigated possible mediators for breast cancer, but specific biomarkers still need further identification [6]. Considering the huge costs of clinical trials and the high attrition rate of drug development, it is particularly important and urgent to explore the potential biomarkers implicated in the occurrence and progression of breast cancer prior to clinical testing.

Human metabolome consists of endogenous and exogenous molecules that represent the metabolic fingerprint of individuals [7, 8]. Considering the closeness of metabolites to both genotype and phenotype, metabolomics is valuable for more clearly elucidating the pathological network underlying diseases [9–11]. Additionally, Nelson et al. demonstrated that a metabolite drug target supported by genetic evidence was twice as likely to gain market approval [12]. Moreover, in recent years, several genome-wide association studies (GWASs) have made great achievements in revealing genetic determinants for comprehensive human metabolome [13–16]. Therefore, we can accurately identify novel and safe drug targets for the prevention of breast cancer at the genetic and metabolomic levels by Mendelian randomization (MR) analysis, an emerging analytical method using genetic variants as a proxy for an exposure to assess the causal relationships between exposure and outcomes without confounding or reverse causality biases [17].

Currently, potential causal associations between several biomarkers and breast cancer have been estimated via MR design. For example, bilirubin and insulin-like growth factor-1 may be the risk factors for breast cancer [18, 19]. However, there is no large-scale MR analysis to systematically screen the human metabolome for promising drug targets of breast cancer so far. The phenome-wide MR (Phe-MR) analysis can also reveal possible side effects of potential drug targets prior to clinical trials [20]. Therefore, we first conducted a large-scale two-sample MR analysis to systematically screen 112 circulating metabolites for identifying the potential causal mediators of breast cancer. Then, a phenome-wide MR analysis of 679 disease traits was further applied to predict target-mediated side effects of metabolite intervention.

## Methods

### Study design

**Fig. 1:**
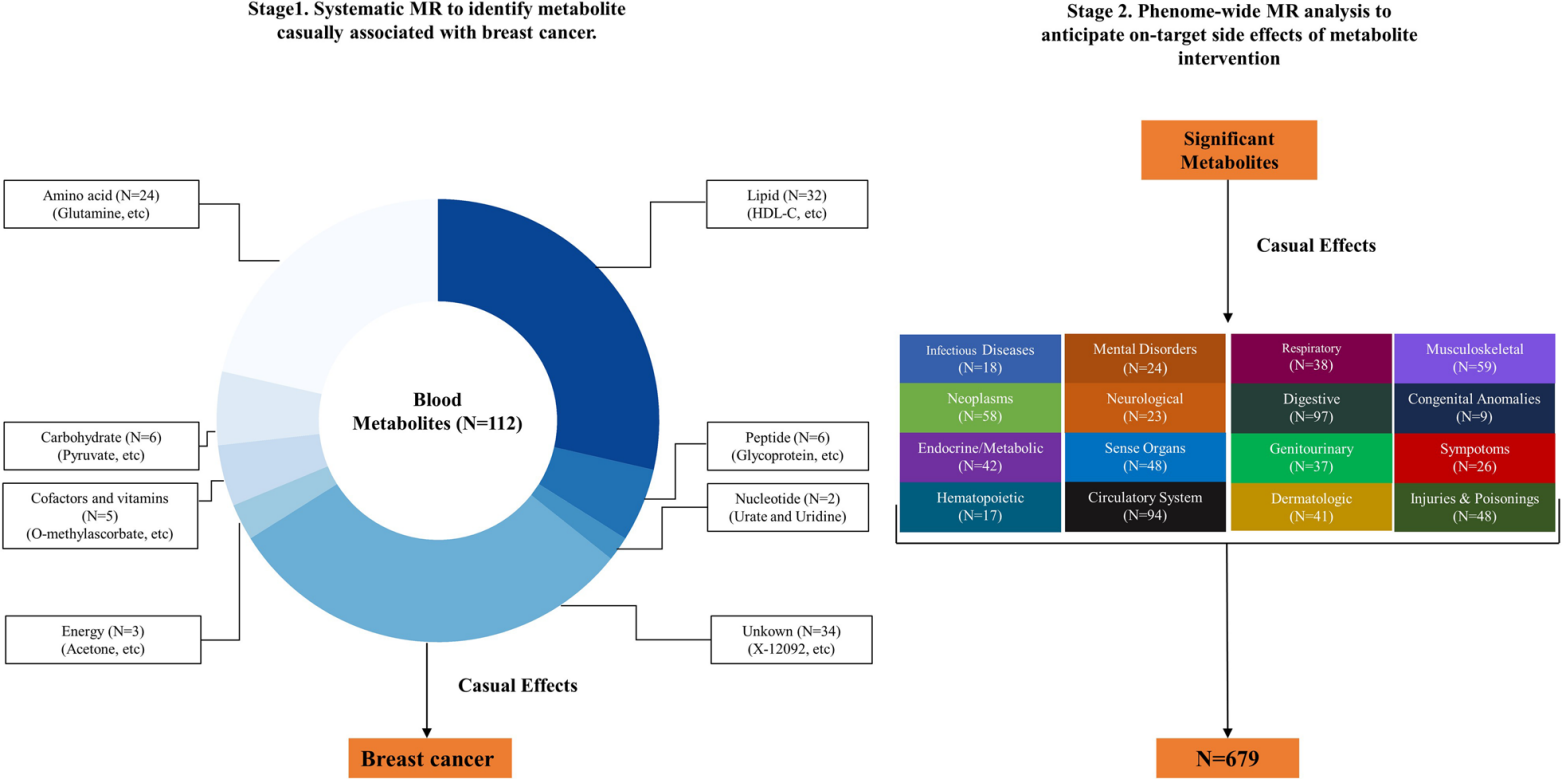
Conceptual framework of two-stage Mendelian randomization (MR) study. The study consists of a two-stage design that employs MR at all stages. First, we assessed the causality for the associations between 112 blood metabolites and the risk of breast cancer. Second, we investigated a broad spectrum of side effects associated with targeting identified metabolites in 679 non-breast cancer diseases. Among these, each disease belongs to one of 16 different International Classification of Disease (ICD)-9 chapters. At each stage, we adopted a Bonferroni-corrected *P* value threshold accounting for both the number of metabolites and diseases analyzed

We conducted a two-stage MR analysis of the blood metabolome to identify potential causal mediators for breast cancer based on the publicly available European-ancestry GWASs (Fig. 1) [13–16, 21]. Ethics approval of the protocol and data collection, and written informed consent from each participant were obtained by the original GWASs.

### Data source for blood metabolome and breast cancer

Summary statistics for genetic variants associated with human metabolome were derived from 3 large-scale GWASs with a total of 147,827 individuals of European ancestry (Table 1) [13–15]. Briefly, Shin et al. [13] analyzed 453 metabolic traits in 7,824 participants with approximately 3 million single nucleotide polymorphisms (SNPs) from two cohorts via Metabolon assay; Kettunen et al. [14] analyzed 123 metabolic traits in 24,925 participants with approximately 12 million SNPs from 14 cohorts via nuclear magnetic resonance assay; and Borges et al. [15] analyzed 249 metabolic traits in 115,078 participants with approximately 12 million SNPs from UK Biobank via Nightingale Health assay (Table 1). The public databases for the above-mentioned metabolites were available from the IEU GWAS database (https://gwas.mrcieu.ac.uk/). These 3 metabolome GWASs measured the actual blood levels of metabolites by nuclear magnetic resonance (NMR) or Metabolon platform, and we used metabolite-related SNPs to reflect the blood metabolites levels at the genetic level in the present MR study. After excluding overlapping metabolites in these 3 metabolome GWASs, a total of 469 metabolites were retained.

**Table 1:** Characteristics of GWASs on the metabolome used for genetic instrument selection

Reference	Cohort(s)	Cohort description	Sample size	Number of metabolic traits	Biomarker assay	Blood fraction tested	Ethnicity	Year
Shin et al	KORA; TwinsUK	Population-based cohort; Population-based twin study	7,824	453	Metabolon	Serum; plasma	German; British	2014
Kettunen et al	EGCUT; ERF; FTC; FR97; COROGENE; GenMets; HBCS; KORA; LLS; NTR; NFBC 1966; PredictCVD; PROTE; YFS	Population-based cohort; Family-based; Population-based twin study; Population-based cohort; Case–control study (only controls used); Case–control study; Birth cohort; Population-based cohort; Family-based; Population-based twin study; Birth cohort; Cohort study; Population-based; Follow up study in children	24,925	123	NMR	Serum; plasma	Estonian; Dutch; Finnish; Finnish; Finnish; Finnish; Finnish; German; Dutch; Dutch; Finnish; Finnish; Estonian; Finnish	2016
Borges et al	UK Biobank	Population-based cohort	115,078	249	Nightingale Health (NMR)	Blood	British	2020

*COROGENE* Genetic Predisposition of Coronary Heart Disease in Patients Verified with Coronary Angiogram, *EGCUT* Estonian Genome Center of University of Tartu Cohort, *ERF* Erasmus Rucphen Family Study, *FR97* a subsample of FINRISK 1997, *FTC* Finnish Twin Cohort, *GenMets* Genetics of METabolic Syndrome, *HBCS* Helsinki Birth Cohort Study, *KORA* Kooperative Health Research in the Region of Augsburg, *LLS* Leiden Longevity Study, *GWAS* genome-wide association study, *NFBC 1966* Northern Finland Birth Cohort 1966, *NMR* nuclear magnetic resonance, *NTR* Netherlands Twin Register, *PredictCVD* FINRISK subsample of incident cardiovascular cases and controls, *PROTE* EGCUT sub-cohort, *YFS* The Cardiovascular Risk in Young Finns Study

Genetic association data of breast cancer were derived from the GWAS conducted by Breast Cancer Association Consortium (BCAC), which is an international collaboration to investigate genetic susceptibility to the risk of developing breast cancer. In brief, this GWAS included 122,977 breast cancer cases and 105,974 controls of European ancestry with 1.06 million SNPs (available from IEU GWAS database: https://gwas.mrcieu.ac.uk/), which was from 68 studies collaborating in BCAC, the Discovery, Biology and Risk of Inherited Variants in Breast Cancer Consortium, the Illumina iSelect genotyping Collaborative Oncological Gene-Environment Study (iCOGS), and 11 other breast cancer GWASs [16]. In BCAC, incident breast cancer cases were recruited from the hospitals and cancer registries [16].

### Genetic instruments of blood metabolites

In the present MR study, SNPs that were identified to be associated with blood metabolites at the genome-wide significance level (*P* value < 5 × 10^–8^) in the published GWASs [13–15] and were not in linkage disequilibrium (LD) with other SNPs (r^2^ < 0.1 within a clumping window of 500 kb) were used as instruments for these blood metabolites. When we encountered certain SNPs above the LD threshold of 0.1, the metabolite-related SNPs with the lowest *P* value were selected. By default, a proxy SNP (r^2^ > 0.8) was selected for the MR analysis in the light of a 1000 Genomes European reference panel if the metabolite-related SNPs were not available in the outcome dataset (i.e., breast cancer dataset). Subsequently, the gtx package in R (version 4.0.3; R Development Core Team) was applied to calculate the phenotypic variance of each blood metabolite explained by the corresponding genetic variations. To ensure sufficient statistical power for a valid causal inference, the metabolites with variance explained by genetic variants less than 0.5% were removed [22]. Furthermore, metabolites with less than 3 correlated SNPs across the genome were also excluded on account of the requirement that at least 3 SNPs should be associated with the exposure in some MR sensitivity analyses [23].

In brief, 357 of 469 metabolites were further excluded according to criteria of the variance explained less than 0.5% or metabolites with the number of associated SNPs less than 3. Finally, a total of 112 unique blood metabolites were included in the MR analysis (Fig. 1). A simplified description of the data concerning SNPs used as instruments in this MR study is listed in Additional file 1: Table S1, and further detailed information is available in Additional file 1: Table S2. F-statistic was used to evaluate the strength of the genetic instruments for blood metabolites. A higher F-statistic indicates a stronger instrument, and a cutoff of 10 is used to distinguish between strong and weak instruments [24].

### Statistical analysis

The inverse-variance weighted (IVW) method was used as our main MR method to detect the causal effects of 112 blood metabolite levels on the risk of breast cancer [25]. Cochran’s Q statistic was applied to estimate the heterogeneity among genetic instruments used in the main analysis [26]. We adopted random-effects IVW model if heterogeneity existed, otherwise fix-effects IVW model was used.

To assess the robustness of causal associations identified via the IVW method, we subsequently conducted a series of sensitivity analyses, including the weighted median approach, the MR-Robust Adjusted Profile Scoring (MR-RAPS), and MR-Egger method [26–28]. The weighted median approach can provide an accurately causal estimate when up to 50% of genetic variants were invalid [26]. We also performed the MR-RAPS analysis due to its resilience to violations of certain assumptions underlying the MR study, such as horizontal pleiotropy and weak instruments [27]. Finally, MR-Egger regression was conducted to ascertain the potential directional pleiotropy via the intercept term [28].

### Phe-MR analysis for on-target side effects of breast cancer-related metabolites

Phe-MR analysis was used to assess the potential on-target side effects associated with hypothetical interventions that reduced the burden of breast cancer by targeting identified metabolites. Genetic association data of 1,403 disease traits with 408,961 white British participants were acquired from Zhou et al.’s GWAS with 28 million SNPs in the UK Biobank cohort (https://www.leelabsg.org/resources) [21]. Disease traits were defined in terms of “PheCodes,” a system developed to organize International Classification of Disease (ICD) codes into phenotypic outcomes suitable for systematic genetic analysis of numerous disease traits [21, 22]. In the present study, sex-specific disease traits and disease traits with cases < 500 were excluded due to the issues of data availability and statistical power, respectively. Additionally, to improve the interpretability of the results, we only selected representative phenotypes to minimize inherent redundancy between PheCodes. Finally, a total of 679 non-breast cancer disease traits were included in the Phe-MR analysis to further characterize the on-target potential side effects of breast cancer-related metabolites (Fig. 1; Additional file 1: Table S3). Genetic instruments for breast cancer-related metabolites were derived from the same GWASs as in the main breast cancer analysis [16]. Based on the associations between metabolites and breast cancer, the final Phe-MR results were normalized to a change in metabolite level corresponding to a 10% reduction in breast cancer risk. We standardized Phe-MR results in this way to discover the side effects of metabolite-targeted interventions for breast cancer and to directly compare the magnitude and direction of the side effects.

All MR estimates were presented as odds ratios (ORs) with 95% confidence intervals (CIs) of outcomes. In stage 1, an observed two-sided *P* < 4.46 × 10^–4^ (Bonferroni-corrected significance threshold calculated as 0.05 divided by 112 [for 112 metabolites]) was used to evaluate statistical significance for a potential causal association. In stage 2, the statistical significance threshold for Phe-MR analysis was set at *P* = 3.68 × 10^–5^, which was corrected for multiple comparisons using the Bonferroni method (0.05/1358 [2 identified breast cancer metabolites in stage 1 × 679 diseases]). A two-sided *P* < 0.05 was considered as suggestive evidence for potential directional pleiotropy in the MR-Egger regression method [29]. All statistical analyses were performed with the packages of gtx, MendelianRandomization, TwoSampleMR, ggplot2, ggrepel, grid, gridExtra, gtable, qqman, RColorBrewer, and RGraphics in R (version 4.0.3; R Development Core Team).

## Results

### Strength of genetic instruments for blood metabolites

A total of 112 unique blood metabolites are included in the present MR study (Additional file 1: Table S2), and the detailed information on genetic instruments for each blood metabolite is shown in Additional file 1: Tables S1, S2. The variance of metabolites explained by genetic instruments ranged from 0.68% to 47.25%. The F-statistics for the genetic instruments of blood metabolites range from 31 to 353, suggesting that there is no weak instrument bias in our MR study (Additional file 1: Table S1).

### Screening the significant blood metabolites for potential causal mediators of breast cancer

**Fig. 2:**
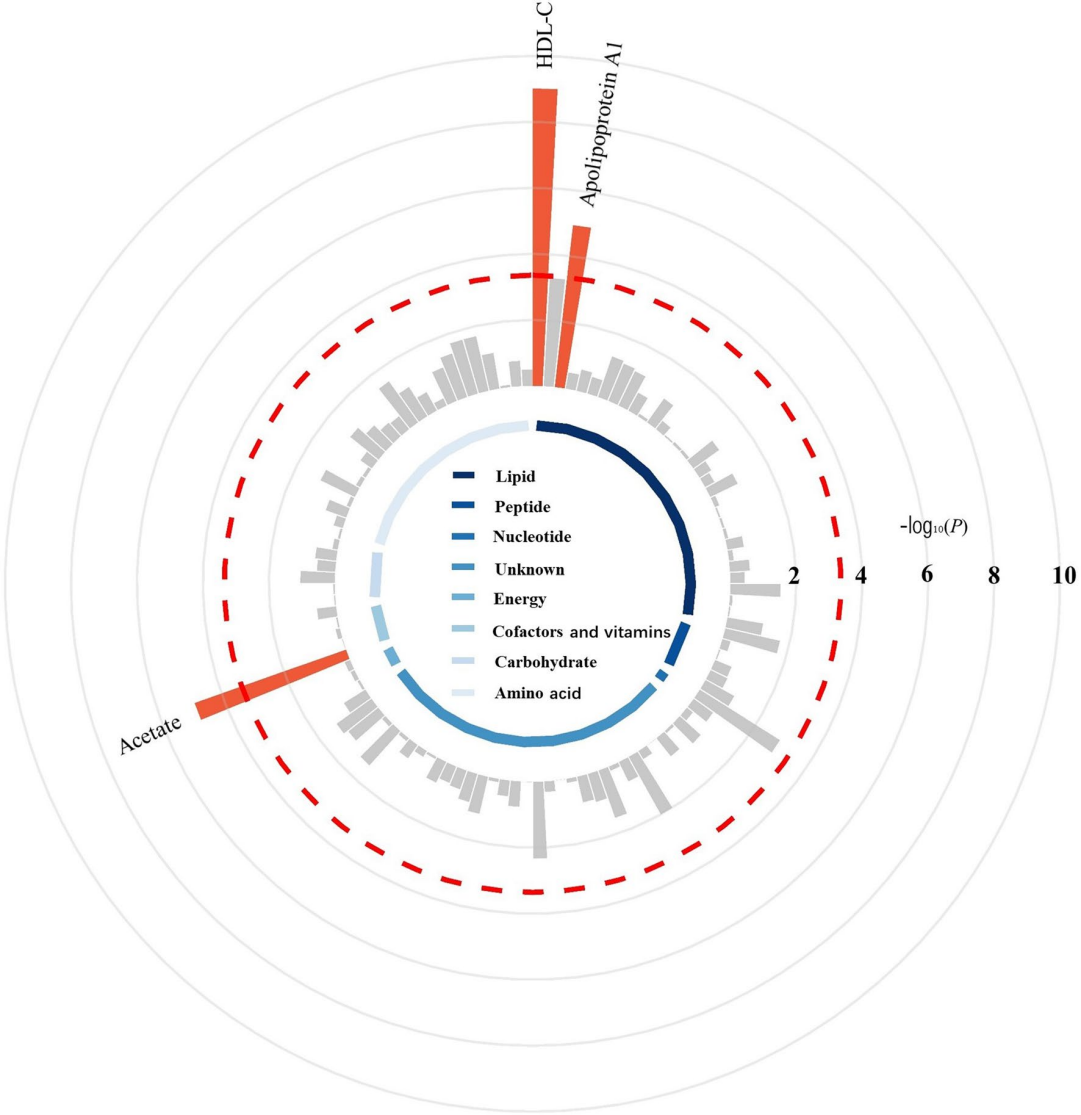
Circular Manhattan plot displaying the associations between blood metabolites and the risk of breast cancer. The red dashed line represents the Bonferroni-corrected significance threshold (*P* < 0.05/112 = 4.46 × 10^–4^), and the labels are provided for significant metabolites. The 112 blood metabolites are grouped and color-coded by super-pathway listed in Table S1. The detailed results for the associations between blood metabolites and breast cancer by inverse-variance weighted Mendelian randomization analysis are presented in Table S4

The IVW method was used to estimate the causal relationships between 112 blood metabolites and the risk of breast cancer in the main MR analysis, and the detailed results are presented in Fig. 2 and Additional file 1: Table S4. Among these 112 unique blood metabolites, genetically determined high levels of high-density lipoprotein cholesterol (HDL-C), apolipoprotein A1, and acetate were significantly associated with an increased risk of breast cancer. We subsequently conducted a series of sensitivity analyses to assess the robustness of our findings in the main analysis. As shown in Additional file 1: Table S5, genetically determined high HDL-C and acetate remained significantly associated with an increased risk of breast cancer in the sensitivity analyses using the weighted median method and MR-RAPS method, and the MR-Egger regression showed no evidence of directional pleiotropy for associations of HDL-C and acetate with the risk of breast cancer. In summary, a total of 2 potential causal mediators were identified for the risk of breast cancer (Table 2). Each SD increase in genetically determined HDL-C (OR, 1.09; 95% CI, 1.06–1.12; *P* = 9.67 × 10^−10^) and acetate (OR, 1.24; 95% CI, 1.13–1.37;* P* = 1.35 × 10^−5^) was associated with a high risk of breast cancer.

**Table 2:** MR analyses for blood metabolites having etiologic associations with breast cancer risks

Metabolite	IVW		Weighted median		MR-RAPS		MR-Egger Intercept
	OR (95% CI)	*P* value	OR (95% CI)	*P* value	OR (95% CI)	*P* value	*P* value
HDL-C	1.09 (1.06–1.12)	9.67 × 10^–10^	1.09 (1.05–1.13)	2.07 × 10^–5^	1.09 (1.07–1.11)	1.85 × 10^–15^	0.16
Acetate	1.24 (1.13–1.37)	1.35 × 10^–5^	1.33 (1.15–1.52)	7.54 × 10^–5^	1.25 (1.13–1.38)	8.89 × 10^–15^	0.13

*IVW* inverse-variance weighted, *MR-RAPS* Mendelian randomization robust adjusted profile score

Odds ratios (ORs) with their 95% confidence intervals (CIs) represent the association estimates with the risk of breast cancer of per 1-SD increase in HDL-C and acetate levels, respectively

Significant threshold in stage 1 was set at *P* < 0.05/112 = 4.46 × 10^–4^ (Bonferroni-corrected significance threshold calculated as 0.05 divided by 112 [for 112 blood metabolites])

### Phe-MR analysis for the associations between identified metabolites and 679 diseases

Phe-MR analysis was further performed to systematically assess the effects of the identified breast cancer metabolites on the risks of 679 non-breast cancer diseases to explore their potential side-effect profiles. Unlike the previous MR, the results of Phe-MR were standardized to a 10% reduction in the risk of breast cancer mediated by targeting a given metabolite. Consequently, resultant associations can be interpreted as concomitant side effects expected to arise if each metabolite is used to prevent breast cancer. In the Phe-MR analysis using the IVW method, a total of 43 associations reached a Bonferroni-corrected significance threshold of *P* = 3.68 × 10^−5^ (0.05/1358 [2 metabolites*679 diseases]) (Additional file 1: Tables S6, S7). In the sensitivity analyses with the methods of weighted median, MR-RAPS and MR-Egger, 4 significant disease associations for HDL-C and 1 disease association for acetate were identified (Additional file 1: Table S8).

**Fig. 3:**
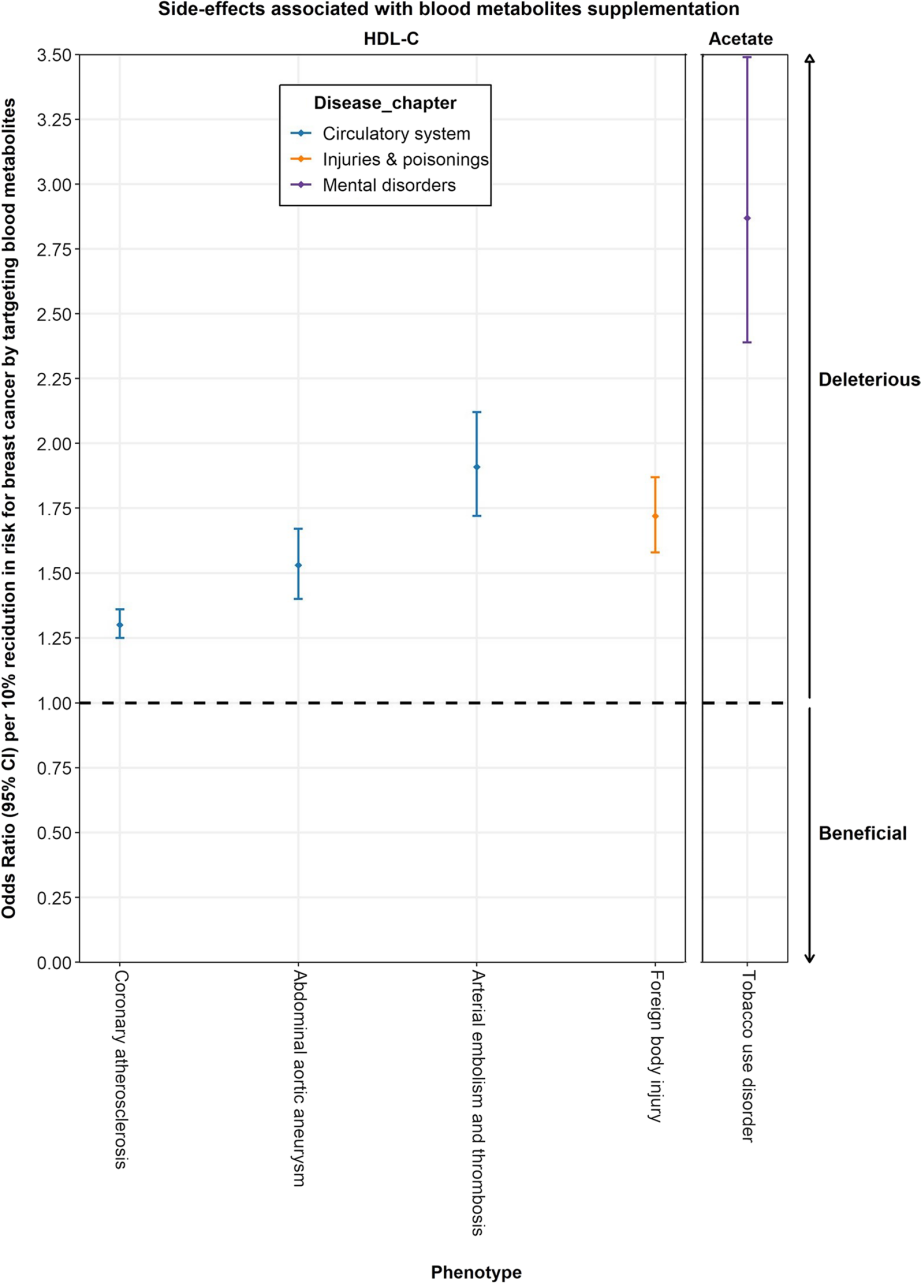
Potential on-target side effects associated with HDL-C and acetate. Odds ratios (ORs) with their 95% confidence intervals (CIs) represent the effect estimates on the risk of multiple non-breast cancer diseases of per 10% reduction in risk for breast cancer by targeting HDL-C and acetate, respectively. Associations above the horizontal black midline represent deleterious side effects. In contrast, associations below the horizontal black midline represent beneficial side effects

Taken together, 5 significant associations were identified for targeting HDL-C and acetate with numerous non-breast cancer diseases (Fig. 3, Table 3, and Additional file 1: Table S9). In brief, lowering HDL-C had detrimental effects on the risk of 4 diseases (3 circulatory system diseases and foreign body injury), and lowering acetate had deleterious effects on tobacco use disorder. The most significant disease associations for HDL-C and acetate were coronary atherosclerosis (OR per 10% reduction in breast cancer risk, 1.30; 95% CI, 1.25–1.36; *P* = 4.13 × 10^−11^) and tobacco use disorder (OR per 10% reduction in breast cancer risk, 2.87; 95% CI, 2.39–3.45; *P* = 6.87 × 10^−9^), respectively (Table 3).

**Table 3:** Phe-MR analyses for causal associations of HDL-C and acetate with the risk of multiple non-breast cancer diseases

PheCode	Outcome	Disease chapter	IVW		Weighted median		MR-RAPS		MR-Egger Intercept
			OR (95% CI)	*P* value	OR (95% CI)	*P* value	OR (95% CI)	*P* value	*P* value
*HDL-C*									
411.4	Coronary atherosclerosis	Circulatory system	1.30 (1.25–1.36)	4.13 × 10^–11^	1.23 (1.18–1.29)	3.96 × 10^–6^	1.32 (1.29–1.35)	2.45 × 10^–33^	0.07
442.11	Abdominal aortic aneurysm	Circulatory system	1.53 (1.40–1.67)	1.04 × 10^–9^	1.49 (1.29–1.73)	7.56 × 10^–6^	1.54 (1.42–1.67)	1.01 × 10^–9^	0.52
444	Arterial embolism and thrombosis	Circulatory system	1.91 (1.72–2.12)	7.01 × 10^–10^	2.24 (1.86–2.69)	1.12 × 10^–5^	1.92 (1.74–2.12)	2.32 × 10^–11^	0.71
1001	Foreign body injury	Injuries and poisonings	1.72 (1.58–1.87)	1.79 × 10^–10^	1.94 (1.65–2.27)	3.01 × 10^–5^	1.73 (1.59–1.88)	2.10 × 10^–10^	0.19
*Acetate*									
318	Tobacco use disorder	Mental disorders	2.87 (2.39–3.45)	6.87 × 10^–9^	3.11 (2.37–4.07)	2.84 × 10^–5^	2.95 (2.44–3.56)	1.21 × 10^–8^	0.92

Odds ratios (ORs) with their 95% confidence intervals (CIs) represent the effect estimates on the risk of multiple non-breast cancer diseases of per 10% reduction in risk for breast cancer by targeting HDL-C and acetate, respectively

Significant threshold in stage 2 was set at set at *P* < 0.05/1358 = 3.68 × 10^–5^, which was corrected for multiple comparisons using the Bonferroni method (0.05/1358 [2 identified breast cancer metabolites in stage 1 × 679 diseases])

*IVW* inverse-variance weighted, *MR-RAPS* Mendelian randomization robust adjusted profile score, *Phe-MR* phenome-wide Mendelian randomization, *SNPs* single nucleotide polymorphisms

## Discussion

By combining metabolomics with genomics, this systematic MR study provided novel clues that would contribute to the search for promising and safe drug targets of breast cancer. Among the 112 blood metabolites, we identified 2 metabolites as potential causal mediators for breast cancer, including HDL-C and acetate. Namely, genetically predicted high HDL-C and acetate levels are associated with increased risks of breast cancer. In addition, Phe-MR analysis was further used to assess the potential on-target side effects associated with breast cancer prevention via lowering HDL-C and acetate. Beyond breast cancer, lowering HDL-C had detrimental effects on 3 circulatory system diseases and foreign body injury, and lowering acetate had deleterious effects on 1 mental disorders disease.

HDL-C is the smallest and densest lipoprotein with the effect of transport triglycerides and cholesterol in the blood [30]. It had been reported that the glycation and oxidation of HDL could lead to abnormal actions on breast cancer cell adhesion to human umbilical vein endothelial cells and extracellular matrix, thereby promoting metastasis progression of breast cancer [31]. In a prospective study within the European Prospective Investigation into Cancer and Nutrition (EPIC)-Heidelberg cohort, high HDL-C levels were shown positively associated with breast cancer risk [32]. An analysis based on 4190 patients with operable breast cancer showed that low levels of HDL-C might be associated with a lower risk of breast cancer recurrence [33]. In another previous MR analysis of circulating lipid traits and breast cancer risk, each 15 mg/dL increase in genetically predicted HDL-C was associated with a 12% increased breast cancer risk [34]. In this large-scale metabolomics MR study, we further confirmed that HDL-C may be a mediator in the development of breast cancer. Besides these, our Phe-MR analysis further extended our knowledge on the potential side effects of lowering HDL-C for the prevention of breast cancer. In the Phe-MR analysis, lowering HDL-C levels were shown to have detrimental effects on the risk of circulatory system diseases and foreign body injury. Overall, although HDL-C may be a drug target for breast cancer, it should be carried out after weighing the advantages and disadvantages of HDL-C.

Acetate, a short-chain fatty acid, has gained increasing focus as a critical regulator of fat mass [35]. It had been reported that acetate in the human body was mainly produced by the intestinal microbes or the liver metabolizing alcohol [36]. Alcohol consumption can induce sustained increases in plasma acetate concentrations, and the increases in plasma acetate are more marked during long-term alcohol consumption [37]. After drinking alcohol, ethanol is broken down in the body to acetaldehyde, which is subsequently broken down to acetate [36]. Interestingly, alcohol consumption is a risk factor for breast cancer, and the World Cancer Research Fund (WCRF) found a 7% increased risk of breast cancer per 10 g alcohol per day [38]. Therefore, further studies about the association between alcohol consumption and breast cancer will likely take into consideration the findings in the present study. Previous studies had shown that acetate was a nutrient source of cancer cells and is closely linked to breast cancer [36, 39, 40]. Schug et al. had identified the dependence of breast cancer and prostate cancer on acetate metabolism [41]. In addition, the ^11^C-acetate positron emission tomography tracers had been used for prostate cancer and hepatocellular carcinoma [42, 43], and our study may provide relevant evidence for the application of ^11^C-acetate positron emission tomography tracers in breast cancer. All in all, based on the data for breast cancer GWAS with 122,977 cases and 105,974 controls of European ancestry, we found that genetically predicted blood acetate levels were positively associated with the risk of breast cancer. This finding was consistent with previous experimental studies and provided population-based evidence that acetate is a potential causal mediator of breast cancer from the viewpoint of genetics. Furthermore, our Phe-MR analysis suggested that lowering acetate levels for preventing breast cancer had a deleterious effect on tobacco use disorder. Therefore, acetate may be a potential drug target for preventing breast cancer, but caution with possible adverse side effects should be taken in the clinic.

Our findings have several important public health and clinical implications. Given that rapidly progressive breast cancer eludes screening and presents at an advanced stage, it is very important to ascertain some promising biomarkers for early identifying individuals at high risk of breast cancer. From the findings of our study, HDL-C and acetate, the potential biomarkers of developing breast cancer, might be served as drug targets for preventing breast cancer. Certainly, further clinical trials are needed to confirm the feasibility and safety of HDL-C and acetate in the prevention of breast cancer, and the validated findings will promote precise prevention for breast cancer.

Our study has some strengths. Firstly, to the best of our knowledge, this is the first systematic MR study using blood metabolites as exposures to estimate their causal effects on the risk of breast cancer. Secondly, the present MR study was conducted on the basis of several large-scale GWASs, which enabled us to make a valid causal inference with high statistical power. Thirdly, our results were robust by means of strict quality control conditions and a series of sensitivity analyses. Fourthly, we further employed the Phe-MR analysis to screen promising drug targets for comprehensively predicting the on-target side effects of identified metabolites.

There are also several limitations that should be noted. Firstly, all GWAS data of the present MR study were from European populations (mostly whites), which might limit the reliability when extrapolating our findings to non-European populations and other races. However, this restriction minimized population and race stratification bias, and further studies are needed to confirm our findings in other populations with different ethnic background. Secondly, although the MR study included 112 different metabolites from three large GWASs via strict selection criteria, these metabolites represent only a small proportion of the blood metabolomes. Therefore, the associations between more blood metabolites and breast cancer required further investigation. Thirdly, the UK Biobank did not collect the fasting blood, while the blood metabolites from Shin et al. [13] and Kettunen et al. [14] were measured in fasting blood. Therefore, possible bias may be caused by different studies collecting blood samples in a different way. Further studies on the basis of larger GWAS with fasting blood samples are warranted to confirm our findings. Finally, our study mainly focuses on the overall incidence of breast cancer and lacked information on breast cancer subtypes. Therefore, it is of clinical interest to investigate the relationship between breast cancer subtypes and blood metabolome merits in the future to provide more information for specific prevention and treatment.

## Conclusions

The present systematic MR analysis revealed that HDL-C and acetate may be the causal mediators in the risk of developing breast cancer. Side-effect profiles were characterized to help inform drug target prioritization for preventing breast cancer. HDL-C and acetate may be promising drug targets for the prevention of breast cancer under weighting advantages and disadvantages.

## Supplementary Information

**Additional file 1**. Supplementary Online Content.

## Data Availability

All summary statistics used in this two-stage Mendelian randomization are available online from each genome-wide association study. Statistical code is available on the request by directly contacting the corresponding author (email: zbzhu@suda.edu.cn).
