# Supplementary material for: Association between human blood metabolome and the risk of breast cancer
Source: Breast Cancer Res. 2023 Jan 24;25:9. doi: 10.1186/s13058-023-01609-4 (PMC9872401; doi:10.1186/s13058-023-01609-4)
Supplement: Supplementary file 1 — Additional file 1. Supplementary Online Content. [file 13058_2023_1609_MOESM1_ESM.docx]

**Association Between Human Blood Metabolome and the Risk of Breast Cancer**

**Supplementary Material**

**Summary of supplementary tables**

| **Table S1** Overview of the blood metabolites included in the MR study. |
| --- |
| **Table S2** Detailed description of the genetic instruments for blood metabolites used in the MR study. |
| **Table S3** 679 diseases incorporated in the Phe-MR analysis. |
| **Table S4** Inverse-variance weighted MR analysis for the associations between blood metabolites and breast cancer. |
| **Table S5** Sensitivity analyses for significant blood metabolites. |
| **Table S6** Phe-MR analyses for the associations between HDL-C and 679 diseases using the inverse-variance weighted method. |
| **Table S7** Phe-MR analyses for the associations between Acetate and 679 diseases using the inverse-variance weighted method. |
| **Table S8** Sensitivity analyses for all significant results in the inverse-variance weighted Phe-MR analysis. |
| **Table S9** Summary of significant Phe-MR findings representing side effects associated with targeting identified metabolites. |

**Table S1. Overview of the blood metabolites included in the MR study.**

| **Metabolite** | **Super-pathway** | **SNPs identified in GWASs** | **SNPs used in MR study** | **Proxy SNPs^*^** | **Sample size** | **Population** | **Variance^‡^** | **F statistic^§^** |
| --- | --- | --- | --- | --- | --- | --- | --- | --- |
| HDL-C | Lipid | 306 | 273 | 1 | 115,078 | European | 20.47% | 109 |
| Total triglycerides | Lipid | 277 | 261 | 4 | 115,078 | European | 16.39% | 90 |
| Apolipoprotein A1 | Lipid | 263 | 243 | 1 | 115,078 | European | 16.58% | 96 |
| Glycoprotein acetyls | Lipid | 220 | 205 | 4 | 115,078 | European | 13.46% | 88 |
| VLDL cholesterol | Lipid | 215 | 202 | 3 | 115,078 | European | 14.06% | 93 |
| Linoleic acid | Lipid | 211 | 197 | 4 | 114,999 | European | 12.06% | 82 |
| Sphingomyelins | Lipid | 209 | 202 | 2 | 114,999 | European | 16.03% | 82 |
| Phosphatidylcholines | Lipid | 209 | 195 | 2 | 114,999 | European | 14.40% | 100 |
| Phosphoglycerides | Lipid | 199 | 187 | 2 | 114,999 | European | 13.16% | 94 |
| Apolipoprotein B | Lipid | 197 | 183 | 1 | 115,078 | European | 16.78% | 128 |
| Total cholesterol | Lipid | 193 | 183 | 0 | 115,078 | European | 14.52% | 107 |
| Docosahexaenoic acid | Lipid | 181 | 174 | 2 | 114,999 | European | 16.52% | 131 |
| LDL cholesterol | Lipid | 176 | 167 | 0 | 115,078 | European | 16.20% | 134 |
| Free cholesterol | Lipid | 39 | 34 | 0 | 13,497 | European | 15.39% | 72 |
| 3-Hydroxybutyrate | Lipid | 26 | 23 | 0 | 113,595 | European | 1.17% | 60 |
| Butyrylcarnitine | Lipid | 25 | 3 | 0 | 7,796 | European | 1.59% | 42 |
| Carnitine | Lipid | 21 | 20 | 0 | 7,797 | European | 16.01% | 74 |
| 4-androsten-3beta,17beta-diol disulfate 1 | Lipid | 6 | 6 | 0 | 7,804 | European | 8.23% | 117 |
| Hexanoylcarnitine | Lipid | 9 | 9 | 0 | 7,786 | European | 11.20% | 109 |
| Octanoylcarnitine | Lipid | 8 | 8 | 0 | 7,790 | European | 10.17% | 110 |
| Androsterone sulfate | Lipid | 8 | 8 | 0 | 7,785 | European | 13.98% | 158 |
| Epiandrosterone sulfate | Lipid | 7 | 7 | 0 | 7,769 | European | 9.31% | 114 |
| 5alpha-androstan-3beta,17beta-diol disulfate | Lipid | 6 | 5 | 0 | 7,345 | European | 5.22% | 81 |
| 3-dehydrocarnitine | Lipid | 6 | 5 | 0 | 7,345 | European | 5.22% | 81 |
| 1-arachidonoylglycerophosphocholine | Lipid | 5 | 5 | 0 | 7,507 | European | 7.55% | 122 |
| Arachidonate (20:4n6) | Lipid | 5 | 5 | 0 | 7,816 | European | 7.25% | 122 |
| Cis-4-decenoyl carnitine | Lipid | 5 | 5 | 0 | 7,660 | European | 6.22% | 101 |
| Decanoylcarnitine | Lipid | 5 | 5 | 0 | 7,766 | European | 6.09% | 101 |
| 1-arachidonoylglycerophosphoinositol | Lipid | 5 | 3 | 0 | 7,797 | European | 2.68% | 71 |
| Propionylcarnitine | Lipid | 5 | 5 | 0 | 7,813 | European | 4.32% | 70 |
| Dihomo-linolenate (20:3n3 or n6) | Lipid | 3 | 3 | 0 | 7,805 | European | 2.08% | 55 |
| 10-undecenoate (11:1n1) | Lipid | 4 | 4 | 0 | 7,806 | European | 5.84% | 121 |
| Glycoproteins | Peptide | 86 | 86 | 0 | 18,734 | European | 34.93% | 116 |
| Albumin | Peptide | 12 | 12 | 0 | 18,960 | European | 4.13% | 68 |
| N-acetylornithine | Peptide | 10 | 10 | 0 | 7,574 | European | 30.75% | 336 |
| Bradykinin, des-arg(9) | Peptide | 5 | 5 | 0 | 4,570 | European | 9.11% | 91 |
| Gamma-glutamyltyrosine | Peptide | 5 | 5 | 0 | 7,468 | European | 2.04% | 31 |
| HWESASXX | Peptide | 3 | 3 | 0 | 7,700 | European | 1.48% | 39 |
| Urate | Nucleotide | 5 | 5 | 0 | 7,819 | European | 7.99% | 136 |
| Uridine | Nucleotide | 3 | 3 | 0 | 7,800 | European | 1.62% | 43 |
| X-12092 | Unknown | 19 | 19 | 0 | 7,500 | European | 47.25% | 353 |
| X-12063 | Unknown | 15 | 10 | 0 | 7,197 | European | 13.69% | 114 |
| X-11593--O-methylascorbate | Unknown | 14 | 13 | 0 | 7,788 | European | 17.24% | 144 |
| X-12798 | Unknown | 13 | 13 | 0 | 7,552 | European | 21.83% | 162 |
| X-11793--oxidized bilirubin | Unknown | 10 | 9 | 0 | 7,611 | European | 10.30% | 97 |
| X-12728 | Unknown | 9 | 9 | 0 | 537 | European | 40.37% | 40 |
| X-11787 | Unknown | 9 | 9 | 0 | 7,811 | European | 15.73% | 162 |
| X-03056--N-[3-(2-Oxopyrrolidin-1-yl)propyl]acetamide | Unknown | 9 | 8 | 0 | 7,812 | European | 11.16% | 123 |
| X-11530 | Unknown | 8 | 8 | 0 | 7,409 | European | 13.40% | 143 |
| X-13431--nonanoylcarnitine | Unknown | 7 | 7 | 0 | 6,591 | European | 13.88% | 152 |
| X-12510--2-aminooctanoic acid | Unknown | 7 | 7 | 0 | 7,566 | European | 13.53% | 169 |
| X-11440 | Unknown | 7 | 7 | 0 | 7,686 | European | 12.14% | 152 |
| X-11442 | Unknown | 7 | 7 | 0 | 7,142 | European | 11.52% | 133 |
| X-11444 | Unknown | 7 | 5 | 0 | 7,758 | European | 5.05% | 82 |
| X-11441 | Unknown | 6 | 6 | 0 | 7,072 | European | 11.19% | 148 |
| X-08402 | Unknown | 6 | 6 | 0 | 7,726 | European | 7.85% | 110 |
| X-12244--N-acetylcarnosine | Unknown | 6 | 6 | 0 | 6,608 | European | 5.28% | 61 |
| X-11261 | Unknown | 6 | 6 | 0 | 7,771 | European | 4.50% | 61 |
| X-12093 | Unknown | 5 | 5 | 0 | 2,854 | European | 17.83% | 124 |
| X-12696 | Unknown | 5 | 5 | 0 | 7,409 | European | 4.99% | 78 |
| X-03094 | Unknown | 5 | 5 | 0 | 7,804 | European | 3.23% | 52 |
| X-11469 | Unknown | 5 | 4 | 0 | 7,779 | European | 2.36% | 47 |
| X-12556 | Unknown | 4 | 4 | 0 | 7,483 | European | 3.12% | 60 |
| X-08988 | Unknown | 4 | 3 | 0 | 7,776 | European | 2.66% | 81 |
| X-02269 | Unknown | 4 | 3 | 0 | 7,701 | European | 1.78% | 46 |
| X-12844 | Unknown | 4 | 4 | 0 | 7,768 | European | 2.23% | 44 |
| X-14205--alpha-glutamyltyrosine | Unknown | 3 | 3 | 0 | 1,789 | European | 6.60% | 42 |
| X-11792 | Unknown | 3 | 3 | 0 | 2,442 | European | 6.44% | 56 |
| X-13435 | Unknown | 3 | 3 | 0 | 6,970 | European | 3.74% | 90 |
| X-10510 | Unknown | 3 | 3 | 0 | 7,792 | European | 2.86% | 77 |
| X-12850 | Unknown | 3 | 3 | 0 | 6,251 | European | 2.13% | 45 |
| X-11315 | Unknown | 3 | 3 | 0 | 7,785 | European | 1.75% | 46 |
| X-09789 | Unknown | 3 | 3 | 0 | 7,805 | European | 1.72% | 45 |
| X-11204 | Unknown | 3 | 3 | 0 | 7,799 | European | 1.27% | 33 |
| Acetone | Energy | 20 | 17 | 0 | 115,075 | European | 0.83% | 59 |
| Succinylcarnitine | Energy | 12 | 12 | 0 | 6,948 | European | 13.56% | 91 |
| Citrate | Energy | 7 | 7 | 0 | 7,813 | European | 3.29% | 38 |
| Acetate | Cofactors and vitamins | 22 | 19 | 0 | 115,046 | European | 0.89% | 56 |
| Biliverdin | Cofactors and vitamins | 9 | 9 | 0 | 6,686 | European | 17.81% | 161 |
| Bilirubin (Z,Z) | Cofactors and vitamins | 8 | 8 | 0 | 6,812 | European | 15.26% | 153 |
| Bilirubin (E,E) | Cofactors and vitamins | 7 | 7 | 0 | 7,748 | European | 8.46% | 102 |
| Bilirubin (E,Z or Z,E) | Cofactors and vitamins | 4 | 4 | 0 | 5,295 | European | 6.38% | 90 |
| Pyruvate | Carbohydrate | 69 | 48 | 1 | 114,748 | European | 2.65% | 67 |
| Glucose | Carbohydrate | 40 | 34 | 1 | 114,867 | European | 2.04% | 72 |
| Lactate | Carbohydrate | 19 | 15 | 1 | 114,802 | European | 0.68% | 55 |
| 1,5-anhydroglucitol (1,5-AG) | Carbohydrate | 7 | 6 | 0 | 7,746 | European | 6.53% | 90 |
| Mannose | Carbohydrate | 6 | 6 | 0 | 7,793 | European | 7.27% | 102 |
| Erythronate | Carbohydrate | 3 | 3 | 0 | 7,752 | European | 1.53% | 40 |
| Glutamine | Amino acid | 117 | 65 | 0 | 114750 | European | 3.37% | 65 |
| Creatinine | Amino acid | 99 | 95 | 1 | 110,058 | European | 4.32% | 51 |
| Valine | Amino acid | 58 | 52 | 0 | 115,048 | European | 3.22% | 78 |
| Histidine | Amino acid | 49 | 30 | 0 | 114,985 | European | 1.43% | 56 |
| Tryptophan | Amino acid | 20 | 18 | 0 | 7,804 | European | 7.55% | 40 |
| Isoleucine | Amino acid | 20 | 19 | 0 | 115,075 | European | 1.15% | 71 |
| Glutaroyl carnitine | Amino acid | 12 | 12 | 0 | 7,701 | European | 11.45% | 83 |
| Leucine | Amino acid | 12 | 12 | 0 | 7,799 | European | 5.45% | 37 |
| Glycine | Amino acid | 9 | 9 | 0 | 7,802 | European | 15.58% | 160 |
| Isobutyrylcarnitine | Amino acid | 8 | 8 | 0 | 7,812 | European | 8.77% | 94 |
| Proline | Amino acid | 7 | 7 | 0 | 7,816 | European | 8.69% | 106 |
| N-acetylglycine | Amino acid | 7 | 7 | 0 | 7,135 | European | 7.30% | 80 |
| Isovalerylcarnitine | Amino acid | 7 | 7 | 0 | 7,789 | European | 6.61% | 79 |
| Betaine | Amino acid | 7 | 5 | 0 | 7,806 | European | 3.82% | 62 |
| 4-acetamidobutanoate | Amino acid | 6 | 6 | 0 | 6,930 | European | 7.16% | 89 |
| Kynurenine | Amino acid | 6 | 4 | 0 | 7,816 | European | 3.00% | 60 |
| Pyroglutamine | Amino acid | 5 | 4 | 0 | 7,800 | European | 2.77% | 55 |
| Asparagine | Amino acid | 4 | 3 | 0 | 7,761 | European | 3.29% | 99 |
| Tryptophan betaine | Amino acid | 4 | 4 | 0 | 7,439 | European | 2.95% | 57 |
| Serine | Amino acid | 3 | 3 | 0 | 7,796 | European | 3.36% | 90 |
| Alpha-hydroxyisovalerate | Amino acid | 3 | 3 | 0 | 7,668 | European | 2.68% | 70 |
| 3-methyl-2-oxovalerate | Amino acid | 3 | 3 | 0 | 7,779 | European | 1.69% | 45 |
| Tyrosine | Amino acid | 4 | 4 | 0 | 7,807 | European | 2.04% | 41 |
| Citrulline | Amino acid | 4 | 4 | 0 | 7,773 | European | 1.76% | 35 |

Abbreviation: MR, Mendelian randomization; SNP, single nucleotide polymorphism.

* Proxy SNPs correlated (r2 >0.8) with SNPs that were not available in breast cancer dataset.

† SNPs excluded for antisense strand alleles in the present MR study.

‡ Phenotypic variance in each blood metabolite explained by used genetic variants in the present MR study.

§ The strength of genetic instruments for each blood metabolite in the present MR study.

**Table S2. Detailed description of the genetic instruments for blood metabolites used in the MR study.**

| **Metabolite** | **SNPs used in stage 1** | **SNPs used in stage 2** |
| --- | --- | --- |
| HDL-C | rs10091649, rs10184004, rs10268632, rs10405357, rs10468017, rs1047891, rs10810374, rs11040322, rs11071374, rs11076170, rs111514504, rs112001035, rs11230800, rs112771035, rs113194763, rs114165349, rs114721139, rs114760566, rs115047514, rs11640954, rs116843064, rs11696696, rs117001569, rs117199990, rs117220229, rs117687565, rs117738782, rs117749052, rs117892711, rs11789603, rs117901517, rs118078695, rs118092024, rs118136762, rs11820504, rs11828763, rs1215112, rs12421131, rs12422125, rs12447986, rs12597428, rs1264372, rs12682492, rs12708983, rs12720917, rs12786130, rs12912415, rs12950754, rs12962112, rs12963212, rs12976739, rs13107325, rs1320700, rs13248499, rs138570705, rs138785496, rs139957766, rs140584594, rs141368429, rs141584077, rs142804168, rs143376213, rs144018203, rs145205375, rs145326144, rs146077850, rs1461728, rs147464145, rs148005124, rs148134535, rs148303195, rs150564454, rs150844304, rs150911013, rs1529577, rs1565097, rs1672867, rs16842, rs16891445, rs1711037, rs172337, rs17269250, rs17301781, rs174578, rs17474890, rs1761457, rs17699030, rs1779823, rs17821274, rs1790781, rs1800777, rs1800961, rs180360, rs181479770, rs185221561, rs185334549, rs186945593, rs1875236, rs187929675, rs188501176, rs190118115, rs199651502, rs200512949, rs2011186, rs2066716, rs2070895, rs2115429, rs2119693, rs2165557, rs2176040, rs2245221, rs2276329, rs2280723, rs2292318, rs2298428, rs2307111, rs247615, rs2494748, rs2507989, rs2642438, rs267738, rs27190, rs2740488, rs2777803, rs2792735, rs283, rs28382814, rs28623088, rs28666039, rs28690720, rs28742908, rs28818616, rs289703, rs289719, rs289727, rs289752, rs291040, rs2925979, rs2978615, rs3289, rs333947, rs34514836, rs34663616, rs34955499, rs35136575, rs35138338, rs35184771, rs35311766, rs35493868, rs35511894, rs35633876, rs357905, rs36018387, rs372074906, rs3768321, rs3794649, rs3811361, rs3859113, rs3892214, rs3898938, rs3899015, rs402465, rs41272663, rs41309280, rs4149307, rs4239651, rs4240624, rs4285809, rs435306, rs439401, rs445093, rs4587963, rs4743765, rs4752973, rs4784770, rs4846921, rs4969141, rs4985155, rs4986970, rs5030789, rs5167, rs55682243, rs559355, rs56050009, rs56129100, rs56132500, rs56208677, rs56271783, rs571848809*(rs191555775), rs57912727, rs58680978, rs5883, rs5896, rs59104589, rs59238099, rs59283948, rs59542880, rs6012281, rs60219248, rs6073958, rs60900172, rs6125085, rs61854123, rs61897792, rs61905067, rs61905084, rs62000866, rs62001835, rs62100587, rs62101705, rs62402708, rs625145, rs652455, rs6586891, rs66514732, rs66806308, rs6734506, rs676210, rs688671, rs7010610, rs7012814, rs7017756, rs71336055, rs71352241, rs7197489, rs7198642, rs7200805, rs7203984, rs7229377, rs7241918, rs72780015, rs72784753, rs72786781, rs72786786, rs72823014, rs72836561, rs72959041, rs74018729, rs75566930, rs75609851, rs75662196, rs75663614, rs76083992, rs76299088, rs76769796, rs76860108, rs769449, rs77027049, rs77437185, rs77509279, rs77842142, rs77960347, rs78058190, rs7810507, rs7817574, rs7845090, rs78893833, rs79546662, rs799158, rs79968526, rs80041799, rs8023503, rs881844, rs9265559, rs9469899, rs9491697, rs964184, rs9644636, rs9687846, rs9938413, rs9947678, rs9953300, rs998584 | rs10091649, rs10184004, rs10268632, rs10405357, rs10468017, rs1047891, rs10774625, rs10810374, rs11040322, rs11071374, rs11076170, rs111514504, rs11230800, rs112771035, rs113194763, rs114165349, rs114721139, rs114760566, rs115047514, rs11609805, rs11640954, rs116843064, rs11696696, rs117001569, rs117199990, rs117209788, rs117220229, rs117687565, rs117738782, rs117749052, rs117892711, rs11789603, rs117901517, rs118078695, rs118092024, rs118136762, rs11820504, rs11828763, rs1215112, rs12230272, rs12421131, rs12422125, rs12447986, rs12597428, rs1264372, rs12682492, rs12708983, rs12720917, rs12786130, rs12912415, rs12950754, rs12963212, rs12976739, rs13107325, rs1320700, rs13248499, rs138570705, rs138785496, rs139957766, rs141368429, rs143376213, rs144018203, rs145205375, rs145326144, rs146077850, rs1461728, rs146459385, rs147464145, rs148005124, rs148134535, rs148303195, rs150564454, rs150844304, rs150911013, rs1529577, rs1565097, rs1672867, rs16842, rs16891445, rs1711037, rs1716409, rs172337, rs17269250, rs17301781, rs174578, rs17474890, rs1761457, rs17696736, rs17699030, rs1779823, rs17821274, rs1790781, rs1800777, rs1800961, rs180360, rs185334549, rs186945593, rs1875236, rs187929675, rs188501176, rs2011186, rs2066716, rs2070895, rs2100500, rs2115429, rs2119693, rs2165557, rs2176040, rs2245221, rs2276329, rs2280723, rs2292137, rs2292318, rs2298428, rs2307111, rs247615, rs2494748, rs2642438, rs267738, rs27190, rs2740488, rs2777803, rs2792735, rs283, rs28382814, rs28623088, rs28666039, rs28690720, rs28742908, rs28818616, rs289703, rs289719, rs289727, rs289752, rs291040, rs2925979, rs3289, rs333947, rs34514836, rs34663616, rs34955499, rs35136575, rs35138338, rs35184771, rs35311766, rs35493868, rs35511894, rs35633876, rs357905, rs36018387, rs36226283, rs3768321, rs3794649, rs3809113, rs3811361, rs3859113, rs3892214, rs3898938, rs3899015, rs3922628, rs3926666, rs402465, rs41272663, rs41309280, rs4149307, rs4239651, rs4240624, rs4285809, rs435306, rs439401, rs445093, rs4587963, rs4743765, rs4752973, rs4765008, rs4784770, rs4846921, rs4969141, rs4985155, rs4986970, rs5030789, rs5167, rs55682243, rs559355, rs56050009, rs56132500, rs56208677, rs56271783, rs570920454, rs58680978, rs5883, rs5896, rs59104589, rs59238099, rs59283948, rs59542880, rs60219248, rs6073958, rs60900172, rs6125085, rs61854123, rs61897792, rs61905067, rs61905084, rs61941676, rs62000866, rs62001835, rs62101705, rs62402708, rs625145, rs652455, rs6586891, rs66514732, rs66806308, rs6734506, rs676210, rs688671, rs7010610, rs7012814, rs7017756, rs7133378, rs71336055, rs7134375, rs71352241, rs7197489, rs7198642, rs7200805, rs7203984, rs7229377, rs7241918, rs72780015, rs72784753, rs72786781, rs72786786, rs72823014, rs72836561, rs72959041, rs7308864, rs74018729, rs75566930, rs75609851, rs75662196, rs75663614, rs76083992, rs76769796, rs76860108, rs769449, rs77027049, rs77437185, rs77509279, rs77842142, rs77960347, rs78058190, rs7810507, rs7817574, rs7845090, rs78893833, rs79546662, rs799158, rs79968526, rs80041799, rs8023503, rs881844, rs921919, rs9469899, rs9491697, rs964184, rs9644636, rs9687846, rs9938413, rs9947678, rs998584 |
| Total triglycerides | rs10102717, rs10109207, rs10160799, rs1021435, rs10214652, rs10455872, rs10458569, rs10892004, rs11122450, rs1117983, rs11208050, rs11216020, rs112259268*(rs72836561), rs1128249, rs113206087, rs113296769, rs113334082, rs1145210, rs114721139, rs114741460, rs115047514, rs116316096, rs1168015, rs116843064, rs116886525, rs116974927, rs117619191, rs11772762, rs117794084, rs11828763, rs11903847, rs11968046, rs11976955, rs12221682, rs1240658, rs12531884, rs12537121*(rs2353556), rs12601919, rs1260326, rs12617848, rs1268353, rs12720816, rs12721041, rs12976739, rs13108218, rs13142655, rs13186403, rs13280055, rs13408252, rs13423088, rs138458033, rs138799654, rs139392920, rs139957766, rs140707095, rs141020647, rs141414463, rs141469619, rs141584077, rs141622900, rs143439747, rs143726520, rs144022883, rs1441775, rs144957189, rs145743281, rs146184004, rs146203232, rs1471251, rs147649176, rs148303016, rs148784079, rs149492745, rs150564454, rs150844304, rs16842, rs16891156, rs17091237, rs17206350, rs17381383, rs174580, rs1801689, rs182118140, rs182611493, rs1835346, rs186696265, rs186782888, rs187582489, rs187929675, rs1883711, rs190118115, rs193108398, rs1963945, rs1998045, rs2000999, rs200149999, rs201117812, rs202049562, rs2035816, rs2081194, rs2081687, rs2092479, rs2116096, rs2119693, rs2165557, rs2186037, rs2228607, rs2391175, rs2395105, rs2409836, rs247616, rs2478236, rs2523659, rs2524129, rs261290, rs261342, rs2678379, rs283, rs28383314, rs28399635, rs28413168, rs28546518, rs28821528, rs2915400, rs2921380, rs2925979, rs2980874, rs3025053, rs328, rs3289, rs34052301, rs34417180, rs35169799, rs35686293, rs35795092, rs36177368, rs36229786, rs363064, rs37538, rs3777420, rs3798167, rs3812316, rs3822842, rs3857142, rs3860847, rs386453, rs3898938, rs3899015, rs393155, rs3936511, rs4006564, rs40270, rs405697, rs41265930, rs41267809, rs41290120, rs41316164, rs41552812, rs4273010, rs4491981, rs4635554, rs4666051, rs4704834, rs4708870, rs4804311, rs4840441, rs486915, rs4921915, rs5015801, rs5112, rs523549, rs526748, rs538174489*(rs72853923), rs55687425, rs55960868, rs56001710, rs56017239, rs56142310, rs57192995, rs58542926, rs59007384, rs60049679, rs60837248, rs614754, rs61742729, rs61904855, rs61905062, rs62128744, rs62140395, rs62222988, rs622591, rs624249, rs632057, rs6439657, rs648253, rs6547820, rs6586891, rs6587973, rs66566471, rs67120644, rs6739502, rs6750775, rs681524, rs684773, rs688359, rs6937722, rs6938550, rs6938647, rs7010610, rs7089698, rs7140110, rs71441083, rs71624136, rs72647336, rs72647352, rs72655677, rs72929768, rs72959041, rs72986845, rs731450, rs73196888, rs73401338, rs73596816, rs74444445, rs74873433, rs75542613, rs7583698, rs7588926, rs75919952, rs75991907, rs7661844, rs7679, rs76993561, rs7741969*(rs35820711), rs7758790, rs77842142, rs78058190, rs783144, rs7831557, rs7832357, rs7835546, rs7837587, rs78442878, rs78824368, rs79153732, rs7946423, rs79598313, rs799158, rs8042174, rs883863, rs920048, rs9295143, rs9347414, rs9355859, rs9365205, rs937813, rs9456577, rs964184, rs9644636, rs9844972, rs9889584, rs998584 |  |
| Apolipoprotein A1 | rs1002687, rs10091649, rs10102352, rs10184004, rs1047891, rs1050388, rs1065853, rs1077835, rs10810374, rs10838687, rs11039238, rs11040055, rs11071374, rs11076170, rs111783138, rs112001035, rs11208032, rs11216259, rs112495680, rs11249925, rs112688782, rs112839628, rs112853430, rs113149294, rs113194763, rs114165349, rs115047514, rs11643781, rs11662691, rs11671872, rs116843064, rs117154602, rs117628555, rs117687565, rs117749052, rs11789603, rs117901517, rs117910839, rs118078695, rs118136762, rs12021623, rs12287106, rs1233396, rs12447986, rs1260326, rs1263149, rs1264350, rs12708983, rs12720917, rs12962112, rs12963212, rs12969081, rs12975366, rs13107325, rs1320700, rs1358980, rs1367117, rs138785496, rs139957766, rs140584594, rs140947438, rs141368429, rs143376213, rs144018203, rs144305743, rs145205375, rs146390218, rs146548146, rs147275653, rs147464145, rs147556125, rs148134535, rs150911013, rs15285, rs1529577, rs1545614, rs1645779, rs1672867, rs16940302, rs1711041, rs172337, rs17240566, rs17269250, rs174574, rs1779823, rs1790781, rs1800961, rs185221561, rs185334549, rs1865834, rs1875236, rs187929675, rs188501176, rs190118115, rs190927521, rs1968493, rs199651502, rs200512949, rs201792139, rs2023473, rs2066716, rs2115429, rs2119693, rs2176040, rs2236252, rs2245793, rs2276329, rs2292318, rs2298428, rs235314, rs247615, rs2494748, rs2569550, rs261290, rs2642438, rs267738, rs27190, rs2740488, rs2792735, rs283, rs28382814, rs28666039, rs28690720, rs289703, rs289719, rs289727, rs289752, rs291040, rs2925979, rs2972559, rs308, rs3130893, rs3136458, rs3289, rs34003087, rs34663616, rs34707604, rs35136575, rs35138338, rs35311766, rs35364714, rs35511894, rs357905, rs36018387, rs36057735, rs36209093, rs3768321, rs3794649, rs3859113, rs3892214, rs3898938, rs402465, rs41272663, rs4149307, rs4239651, rs4240624, rs4263041, rs4285809, rs429358, rs445093, rs449647, rs4587963, rs4743765, rs4752890, rs4784717, rs4803760, rs4846921, rs4969141, rs4986970, rs501942, rs5167, rs525302, rs559355, rs56129100, rs56132500, rs56208677, rs572301360*(rs1058729), rs583104, rs58680978, rs5883, rs59238099, rs6012281, rs60219248, rs60455398, rs60900172, rs6125085, rs61854123, rs62000866, rs62000939, rs62001835, rs62100587, rs62117161, rs62117512, rs62119267, rs625145, rs652455, rs6586891, rs6587973, rs676210, rs686030, rs6993714, rs7003526, rs71352241, rs7198642, rs7200805, rs7229377, rs72786781, rs72786786, rs72836561, rs72913825, rs72925817, rs73624696, rs737338, rs7388248, rs74018729, rs75129833, rs75566930, rs75662196, rs75663614, rs75911530, rs76769796, rs76967117, rs77027049, rs77197725, rs7783857, rs77960347, rs78893833, rs78965095, rs8023503, rs8045855, rs881844, rs899228, rs907866, rs9268812, rs9304381, rs9471972, rs9491697, rs967645, rs9788871, rs9938413, rs9947678, rs9953300, rs9972789 |  |
| Glycoprotein acetyls | rs1005599, rs10160799, rs10189899, rs10245965, rs10405357, rs10455872, rs1076158, rs10865959, rs1111462, rs11208654, rs112291945, rs112875651, rs113354603, rs113452856, rs113580328, rs113816145*(rs79307388), rs115550566, rs115795737, rs11645475, rs11648003, rs11648622, rs1168032, rs11682583, rs116843064, rs116992380, rs117026536, rs117155836, rs117365732, rs11757660, rs117655998, rs117753658, rs117841020, rs117847715, rs117870898, rs11828763, rs12032372, rs12073837, rs12110481, rs12221682, rs1249749, rs12537121*(rs2353556), rs12546944, rs12598219, rs1260326, rs1268353, rs13108218, rs13408252, rs138910759, rs138913052, rs139273281, rs139781980, rs140110174, rs141378803, rs141414463, rs141428740, rs143307443, rs143588012, rs143786320, rs144018203, rs144499991, rs144883114, rs145408202, rs146203232, rs148845156, rs148967059, rs149386302, rs149807892, rs150617, rs150844304, rs1548306, rs16971543, rs16973000, rs17105232, rs17289227, rs1801689, rs180728024, rs181810147, rs182050989, rs186639850, rs1894553, rs1945391, rs202049562, rs2042439, rs204893, rs2070634, rs2157051, rs2253491, rs2266943, rs2278984, rs2294915, rs2353082, rs236012, rs2409784, rs2434886, rs2445818, rs2497337, rs2763981, rs2787337, rs2787351, rs28383207, rs28529476, rs2857694, rs28834434, rs28929474, rs2925979, rs2980874, rs2980883, rs3130668, rs3131014, rs34010237, rs34821369, rs34881399, rs35175534, rs35366052, rs4006564, rs41263825, rs41552812, rs4407894, rs4459081, rs4666042, rs4713460, rs4841472, rs4905179, rs5023763, rs55702379, rs55776462, rs55780214, rs56001710, rs56041751, rs56177707, rs56188865, rs56212732, rs58542926, rs59254395, rs59296513, rs59774409, rs60452138, rs60944058, rs62053846, rs62058275, rs62128744, rs62131877, rs62140395, rs62259939, rs62466318, rs62496677, rs62523861, rs6424109, rs6452937, rs6575424, rs6601299, rs6714780, rs6717858, rs6734238, rs676210, rs6764884, rs67934740, rs687621, rs6910879*(rs116018794), rs71403860, rs7196440, rs72655677, rs72700362, rs72787084, rs72801474, rs72803196, rs73632745, rs7404577, rs74873433, rs75563047, rs76108702, rs76473767, rs76743497, rs76844366, rs7697204, rs77009508, rs77303550, rs7744253, rs77535061, rs7758790, rs78417740, rs78939685, rs7924036, rs79287178, rs79315071, rs79440651, rs79636708, rs799158, rs8015929, rs9266184, rs9270074, rs9272445, rs9273651, rs9274062, rs9295128, rs9302635, rs937813, rs9391844, rs9457827, rs9469250*(rs2859109), rs964184, rs9921494, rs9922718, rs9930140, rs9938539 |  |
| VLDL cholesterol | rs10096633, rs10214652, rs10221768, rs10260606, rs10458569, rs1065853, rs1081106, rs11102964, rs11206517, rs112634605, rs1128249, rs112875651, rs1145210, rs115445558, rs1158784, rs11591147, rs116316096, rs116530815, rs11680233, rs1168034, rs116843064, rs116962777, rs117159625, rs117261169, rs11755689, rs117804693, rs12151108, rs12208357, rs12221682, rs12462573, rs12546944, rs1260326, rs1268353, rs12708983, rs12714102, rs12720917, rs12721041, rs12740374, rs12916, rs12983316, rs13108218, rs1337247, rs138458033, rs139100794, rs139524394, rs139974673, rs140912273, rs141414463, rs141469619, rs141584077, rs142084074, rs144261139, rs145790091, rs146576912, rs1471251, rs148333241, rs148356565, rs150856119, rs151000110, rs151330717, rs157595, rs16891156, rs17035665, rs17721559, rs17882028, rs1800777, rs1801177, rs1801695, rs1865834, rs186696265, rs186782888, rs187429064, rs187929675, rs1883711, rs190118115, rs192955957, rs1985129, rs1989985, rs199717562, rs200149999, rs203711, rs2186037, rs2326077, rs247615, rs247616, rs2478236, rs2523675, rs2570344, rs261290, rs261342, rs28399607, rs28399654, rs28550053, rs28607776, rs28917234, rs289744, rs289752, rs2902941, rs2980874, rs2980883, rs2986164, rs3210176, rs34335269, rs34429995, rs34693282, rs34894639, rs35449889, rs35836101, rs369558950, rs377122620, rs3777411, rs3777420, rs3794695, rs386453, rs3899015, rs3936511, rs395908, rs4006564, rs41551116, rs4299376, rs437444, rs4456810, rs449647, rs4635554, rs4665698, rs4704834, rs4705000, rs4708870, rs472495, rs4803748, rs4846914, rs486915, rs4876611, rs4970835, rs507766, rs536421612*(rs140780894), rs540662190*(rs7255743), rs55634260, rs55747707, rs56208677, rs56325564, rs56393506, rs579890, rs58542926, rs59379014, rs60049679, rs60403635, rs6102034, rs611060, rs62115552, rs62117161, rs62119320, rs62138973, rs62141288, rs622591, rs635634, rs6511721, rs651649, rs654689, rs6547409, rs6587973, rs6602909, rs662138, rs67120644, rs6729843, rs6857, rs693, rs6938647, rs7010610, rs7012891, rs7203984, rs7205692, rs7254133, rs72655677, rs72703210, rs72786781, rs72929768, rs73013176, rs73401338, rs73597688, rs740516, rs74434374, rs74717300, rs7519205, rs7523141, rs7543163, rs75919952, rs76272805, rs76976871, rs77082266, rs77303550, rs7750288, rs77542162, rs79429216, rs799158, rs8042174, rs822928, rs907866, rs9302635, rs937813, rs9616847, rs964184 |  |
| Linoleic acid | rs1002687, rs10214652, rs10401176, rs10455872, rs10458569, rs1065853, rs1077835, rs10838724, rs11216100, rs11239569, rs11250098, rs112853430, rs112875651, rs114863007, rs115413710, rs115478735, rs11591147, rs116316096, rs117159625, rs117261169, rs11828763, rs11887534, rs12067569, rs12208357, rs12221682, rs1233489, rs12594571, rs1260326, rs1268353, rs12907378, rs12948283, rs13108218, rs1320700, rs139524394, rs139957766, rs140231532, rs140912273, rs141414463, rs141469619, rs142158911, rs142635674, rs143376213, rs143726520, rs144261139, rs144545816, rs145400326, rs145790091, rs1461729, rs146576912, rs148333241, rs148349043, rs151000110, rs151330717, rs16891156, rs1692821, rs17388017, rs174564, rs1787328, rs1800009, rs181765708, rs182611493, rs183409343, rs185221561, rs186696265, rs186782888, rs1883711, rs188501176, rs193108398, rs1968493, rs198470, rs199869587, rs200149999, rs201125976, rs2071474, rs2115429, rs2239013, rs2245793, rs2284178, rs2378390, rs247617, rs2504922, rs261290, rs2736594, rs2736604, rs2740488, rs28399607, rs2844795, rs2854277, rs28607776, rs28646006, rs28666039, rs28917234, rs289713, rs289716, rs2927439, rs2976940, rs2980874, rs2986164, rs3130668, rs3179865, rs34121855, rs34232196, rs34653400, rs35138338, rs35311766, rs36018387, rs368424410, rs377122620, rs3777420, rs3798167, rs3810143, rs395908, rs4008004, rs402465, rs4075905, rs4149307, rs429358, rs4299376, rs4426495, rs445093, rs449647, rs4665698, rs4704210, rs4704262, rs4708870, rs486142, rs516226, rs533617, rs534417, rs535772, rs540713, rs558121619*(rs9275263), rs558702, rs560898656(rs28399653), rs56113850, rs56322906, rs57465754, rs579890, rs58542926, rs59774409, rs60049679, rs602633, rs61897792, rs62000866, rs62100587, rs62119267, rs62141288, rs622591, rs6453106, rs6471717, rs648253, rs6507945, rs6511721, rs6587973, rs6602911, rs662138, rs668948, rs67120644, rs6859, rs6882345, rs688359, rs688456, rs693, rs6938647, rs7005363, rs7118175, rs7124275, rs7229377, rs72655677, rs72902594, rs72929768, rs73401338, rs73487492, rs73664373, rs740006, rs740516, rs7519205, rs77449055, rs7750288, rs77524918, rs7771138*(rs34107231), rs77960347, rs7816447, rs79429216, rs8042174, rs8111874, rs8192701, rs907866, rs9261608*(rs6932954), rs9264960, rs9265942, rs9268671, rs9272302, rs9302635, rs9304381, rs964184, rs9953300 |  |
| Sphingomyelins | rs10102352, rs102275, rs1057208, rs10822145, rs11097807, rs11102964, rs111278137, rs112027066, rs11206515, rs112172017, rs11230741, rs112875651, rs112952893, rs1135062, rs115445558, rs115740542, rs11591147, rs116843064, rs11691986, rs116962591, rs117026536, rs117261169, rs11751347, rs117901517, rs117983270, rs118078695, rs118136762, rs12067569, rs12151108, rs12546962, rs1260326, rs12720917, rs12721030, rs12740374, rs12924331, rs12974200, rs12979820, rs13107325, rs1320700, rs13211653(rs72848230), rs13337205, rs13396400, rs139957766, rs140912273, rs142213240, rs144261139, rs145790091, rs146576912, rs149754235, rs1500187, rs150342704, rs150639794, rs150856817, rs151000110, rs151330717, rs1634791, rs16940126, rs16940302, rs16962014, rs1711041, rs17301746, rs174467, rs174569, rs17699030, rs1791786, rs1800777, rs1800961, rs181765708, rs185221561, rs188099946, rs188880086, rs198476, rs199717562, rs200756447, rs204480, rs2070895, rs2245793, rs2268702, rs2281721, rs247615, rs2479413, rs2519093(rs115478735), rs261290, rs2642438, rs268, rs2727260, rs2740488, rs2792735, rs2820232, rs28399637, rs28399657, rs28666039, rs28690720, rs287227, rs28834434, rs289716, rs2925979, rs2943650, rs2965162, rs333948, rs34514836, rs34792, rs35088884, rs35138338, rs35511894, rs35633876, rs36018387, rs372642577, rs3764261, rs3768321, rs3800461, rs387137, rs391515, rs395908, rs4008004, rs402465, rs405509, rs4149307, rs4263041, rs429358, rs4299376, rs445093, rs449647, rs4752881, rs4752973, rs4803748, rs485186, rs4860948, rs4970835, rs558971, rs55964643, rs56015824, rs56030824, rs56208677, rs562172, rs562338, rs56325564, rs56394238, rs57465754, rs581080, rs58542926, rs5883, rs59752567, rs60049679, rs60318332, rs611060, rs61897792, rs62000866, rs62001835, rs62115552, rs62117161, rs624698, rs6499863, rs6507945, rs6511721, rs651649, rs6586891, rs661278, rs6728593, rs673335, rs67616858, rs6859, rs6882345, rs695867, rs7111404, rs7115739, rs7118175, rs7203984, rs7229377, rs7247937, rs72836561, rs73013176, rs73048351, rs73189551, rs73664373, rs740006, rs74018729, rs7412, rs74607435, rs75392670, rs76423146, rs77020029, rs77027049, rs77278227, rs77437185, rs77542162, rs77960347, rs78893833, rs78912080, rs78921492, rs7941030, rs79787170, rs8042174, rs8192701, rs9304381, rs949790, rs952274, rs964184, rs9644636, rs9769088, rs984976, rs9987289 |  |
| Phosphatidylcholines | rs1002687, rs10102352, rs10458569, rs1065853, rs1077835, rs10838687, rs10838724, rs11122449, rs11208032, rs11239571, rs112688782, rs112853430, rs112875651, rs112952893, rs113629348, rs11591147, rs116316096, rs11668327, rs117488242, rs11755689, rs117597286, rs117687565, rs117749052, rs11780884, rs11789603, rs117901517, rs118078695, rs118136762, rs12022410, rs1260326, rs12691202, rs12701713, rs12708983, rs12720820, rs12720917, rs12907378, rs12924331, rs12963212, rs13107325, rs1320700, rs140525318, rs141414463, rs141936239, rs142158911, rs142187704, rs145521244, rs147275653, rs150896559, rs16940147, rs16940262, rs16940302, rs1711041, rs17240566, rs17269250, rs17301746, rs17411168, rs17440824, rs174565, rs17657001, rs17821298, rs1787328, rs1790796, rs1800777, rs1800961, rs182611493, rs183409343, rs185221561, rs186696265, rs188501176, rs190118115, rs1942417, rs1968493, rs2000999, rs200149999, rs200501619, rs2043082, rs2071379, rs2245793, rs2286276, rs2389602, rs247615, rs261264, rs2642438, rs2740488, rs2792735, rs2820232, rs2854277, rs28607776, rs28666039, rs289716, rs289752, rs3132685, rs322127, rs34002646, rs34514836, rs34663616, rs34990794, rs35030383, rs35138338, rs35311766, rs35511894, rs35686293, rs36018387, rs368424410, rs370878539, rs3764261, rs3800461, rs391515, rs395908, rs40059, rs4008004, rs4149307, rs4240624, rs445093, rs4542884, rs4708870, rs4775075, rs4860948, rs494606, rs4986970, rs501942, rs5167, rs525028, rs536271135*(rs75881220), rs55968391, rs56208677, rs56960668, rs5754102, rs581080, rs58199976, rs5883, rs59752567, rs59999923, rs60049679, rs60219248, rs60403635, rs60455398, rs6090719, rs61905076*(rs61905077), rs62000866, rs62001835, rs62100587, rs62138973, rs635634, rs6499863, rs6507945, rs6587973, rs676388, rs6882345, rs6886841, rs693, rs6938647, rs709822, rs7124275, rs7203984, rs7229377, rs72737411, rs72740871, rs72743576, rs72786781, rs72836561, rs72913825, rs72925817, rs72929768, rs72997616, rs7349418, rs737338, rs74018729, rs7551124, rs7566040, rs77021177, rs77027049, rs77303550, rs77449055, rs77524918, rs77960347, rs78359532, rs78893833, rs78936960, rs78965095, rs79119207, rs79429216, rs79489590, rs8042174, rs8107974, rs8192701, rs907866, rs9265965, rs9269386, rs9302635, rs9304381, rs937813, rs9788871, rs9953300, rs998075 |  |
| Phosphoglycerides | rs1002687, rs10102352, rs102275, rs10458569, rs1065853, rs1077835, rs10838687, rs11122449, rs111868473, rs11208032, rs11239571, rs112853430, rs112875651, rs112952893, rs113629348, rs115413710, rs11591147, rs116316096, rs11668327, rs117488242, rs11755689, rs117687565, rs117749052, rs11789603, rs117901517, rs118078695, rs118136762, rs11820589, rs12022410, rs12364432, rs1260326, rs12701713, rs12720820, rs12720917, rs12907378, rs12924331, rs12963212, rs13107325, rs13108218, rs1320700, rs141936239, rs142158911, rs142187704, rs145521244, rs147275653, rs150896559, rs1561139, rs1659684, rs16891156, rs16940147, rs16940302, rs1711041, rs17240566, rs17269250, rs17301746, rs17411168, rs17657001, rs1787328, rs1790796, rs1800777, rs1800961, rs182611493, rs182636083, rs183409343, rs183640608, rs185221561, rs186696265, rs1883711, rs188501176, rs1942417, rs200149999, rs200501619, rs2018791, rs202049562, rs2071379, rs2245793, rs2286276, rs247615, rs2596473, rs261264, rs261290, rs2642438, rs2740488, rs2792735, rs2820232, rs2853940, rs2854277, rs28607776, rs28666039, rs289716, rs2980883, rs3087611, rs3130668, rs3132685, rs34002646, rs34663616, rs34990794, rs35138338, rs35246381, rs35311766, rs35511894, rs35686293, rs36018387, rs368424410, rs370878539, rs3764261, rs3770586, rs3800461, rs391515, rs40059, rs4008004, rs402465, rs4149307, rs445093, rs4704224, rs4708870, rs4860948, rs494606, rs4986970, rs515688, rs5167, rs525028, rs536271135(rs75881220), rs55730499, rs56030824, rs56208677, rs5754104, rs581080, rs58542926, rs5883, rs59752567, rs59999923, rs60049679, rs60219248, rs60455398, rs6090719, rs61905076(rs61905077), rs61906115, rs62000866, rs62001848, rs62100587, rs62138973, rs635634, rs6499863, rs6507945rs6547820, rs6587973, rs676388, rs6882345, rs693, rs6938647, rs709822, rs7203984, rs7229377, rs72655677, rs72743576, rs72786781, rs72836561, rs72925817, rs72929768, rs72997616, rs7349418, rs737338, rs74018729, rs7551124, rs7566040, rs76483627, rs77021177, rs77027049, rs77188937, rs77303550, rs77449055, rs77524918, rs77960347, rs78893833, rs78936960, rs79429216, rs8042174, rs8192701, rs907866, rs9265965, rs9269386, rs9304381, rs937813, rs9953300, rs998075, rs9987289 |  |
| Apolipoprotein B | rs102275, rs10260606, rs10460182, rs1081106, rs11102964, rs111278137, rs112027066, rs11206513, rs11206517, rs112172017, rs112875651, rs115445558, rs115478735, rs11571787, rs1158784, rs11591147, rs11601507, rs11679386, rs11691986, rs11755689, rs118147862, rs12151108, rs12208357, rs12239737, rs12327715, rs12462473, rs12546944, rs1260326, rs12740374, rs12916, rs12983316, rs1337247, rs13702, rs139100794, rs139995984, rs140824606, rs141989097, rs142213240, rs143843429, rs144261139, rs144545816, rs145400326, rs145790091, rs146390218, rs146576912, rs148333241, rs148356565, rs148601586, rs149450221, rs1499279, rs1500188, rs150262789, rs150856817, rs151000110, rs151330717, rs17035665, rs17249001, rs1800777, rs181765708, rs1836278, rs1883711, rs190712692, rs199717562, rs200149999, rs200756447, rs201269048, rs203711, rs204480, rs204540, rs2107449, rs2326077, rs247616, rs2570344, rs261290, rs261334, rs2618566, rs28399637, rs28399664, rs28550459, rs28597716, rs28607776, rs2965157, rs2980874, rs35057129, rs35836101, rs372642577, rs377122620, rs3794695, rs3798167, rs41279684, rs4263041, rs429358, rs4299376, rs4426495, rs45518133, rs4665698, rs4704262, rs4704834, rs472495, rs4803743, rs4803748, rs4803787, rs4803791, rs4804510, rs4876611, rs4927206, rs4927214, rs4970835, rs4971548, rs507766, rs519113, rs533617, rs553427, rs55634260, rs55714927, rs56113850, rs562338, rs56325564, rs58542926, rs59379014, rs60049679, rs6016505, rs6102034, rs611060, rs62116311, rs62118464, rs648253, rs6511721, rs651649, rs6601299, rs6602909, rs661278, rs662138, rs67616858, rs6859, rs6920309, rs693, rs6938647, rs71364511, rs71435586, rs7203984, rs7246100, rs7246666, rs7247937, rs7249244, rs7251161, rs72655677, rs72660539, rs72694391, rs72703210, rs72902594, rs72929768, rs73013176, rs73048351, rs7343130, rs73765973, rs740516, rs74607435, rs7523141, rs75237799, rs76186504, rs76670936, rs7700719, rs77241309, rs77278227, rs77303550, rs7737496, rs7750288, rs77542162, rs7757696, rs7773004, rs79220007, rs79429216, rs79915079, rs8042174, rs8107530, rs8113311, rs9295128, rs9302635, rs9616847, rs964184, rs9682783, rs9973228 |  |
| Total cholesterol | rs1002687, rs102275, rs10401176, rs1057558, rs10838724, rs11102964, rs111278137, rs111279811, rs112027066, rs11206513, rs11208056, rs112172017, rs112875651, rs1135062, rs114863007, rs115445558, rs115478735, rs115740542, rs11591147, rs11621792, rs11666995, rs11691986, rs117026536, rs12022410, rs12067569, rs12151108, rs12208357, rs12327715, rs12448528, rs1260326, rs12740374, rs12974200, rs12979820, rs12981080, rs1320700, rs1337247, rs139995984, rs140824606, rs142213240, rs144261139, rs144545816, rs145790091, rs1461729, rs146390218, rs146576912, rs147841808, rs148333241, rs149450221, rs1500188, rs150342704, rs150856817, rs151000110, rs151330717, rs1634791, rs16940126, rs17035665, rs174467, rs1800769, rs1800777, rs1800961, rs181479770, rs185221561, rs1864163, rs188099946, rs1883711, rs190712692, rs1917754, rs199717562, rs200149999, rs200756447, rs201269048, rs2015257, rs204480, rs219546, rs2326077, rs261290, rs261334, rs2618566, rs2642438, rs2740488, rs2762335, rs2792735, rs28399637, rs28399664, rs28550459, rs28607776, rs28666039, rs28752534, rs289716, rs2927439, rs2943650, rs2965162, rs34449399, rs34514836, rs35138338, rs35449084, rs36018387, rs372642577, rs377122620, rs377290437, rs3777411, rs3794695, rs384653, rs3846662, rs4008004, rs402465, rs405509, rs41279684, rs41290120, rs4149307, rs4263041, rs429358, rs4299376, rs4307732, rs4507059, rs4703665, rs4704262, rs472495, rs4803743, rs4860948, rs4927206, rs4959063, rs496654, rs4970835, rs525028, rs533617, rs562338, rs56325564, rs57465754, rs58542926, rs60049679, rs6016505, rs602662, rs611060, rs61946991, rs62000866, rs62115552, rs6507945, rs6511721, rs651649, rs6587973, rs661278, rs662138, rs6728593, rs67616858, rs6859, rs6882345, rs6908402, rs693, rs6938647, rs7012814, rs7229377, rs7246666, rs7247937, rs7251161, rs72836561, rs72902594, rs72929768, rs73013176, rs73048351, rs7343130, rs73664373, rs740516, rs74607435, rs7523141, rs76186504, rs76498378, rs7700719, rs77278227, rs77303550, rs7750288, rs77542162, rs77960347, rs78921492, rs79429216, rs79890446, rs79915079, rs8042174, rs8113311, rs907866, rs9302635, rs9304381, rs9973228 |  |
| Docosahexaenoic acid | rs10102325, rs1077835, rs11075278, rs11122450, rs11230741, rs11230749, rs11230759, rs11230829, rs112687416, rs112875651, rs114863007, rs115428654, rs11591147, rs11603763, rs116262432, rs11681659, rs116962591, rs117026536, rs117110139, rs117186302, rs117414940, rs117486534, rs117876551, rs117901517, rs117983270, rs118078695, rs12362237, rs1260326, rs12786457, rs12799017, rs12805902, rs13424225, rs138012803, rs138110361, rs139957766, rs139974673, rs140699316, rs141502961, rs142158911, rs14415, rs145786300, rs145908530, rs146651998, rs147981159, rs150156331, rs150684478, rs1675090, rs16940126, rs16940904, rs17190510, rs17200189, rs17269250, rs1729377, rs174467, rs174480, rs174528, rs174569, rs174624, rs17821298, rs1791786, rs1791796, rs1794072, rs1800978, rs181479770, rs182611493, rs183130, rs183986993, rs185221561, rs188370817, rs188880086, rs190921611, rs191790331, rs198442, rs198476, rs1993598, rs200149999, rs2009875, rs2229738, rs2232143, rs2239370, rs2278426, rs2394976, rs2524292, rs259872, rs259873, rs261291, rs2727260, rs273912, rs2807967, rs2853968, rs28666039, rs28690720, rs289713, rs2924438, rs3132685, rs34663616, rs34707604, rs34780867, rs34793, rs35138338, rs35177659, rs35429473, rs35511894, rs35739561, rs35827276, rs36018387, rs36030967, rs370878539, rs387137, rs390888, rs4343027, rs4414241, rs445093, rs482548, rs4860309, rs4986970, rs5026246, rs525028, rs55891451*(rs9332220), rs55901398, rs55938136, rs562338, rs58542926, rs59752567, rs61896137, rs61897792, rs62000866, rs62036306, rs62037519, rs62039124, rs638714, rs6507945, rs660240, rs673335, rs67951111, rs6931604, rs694954, rs695867, rs7115739, rs7116318, rs7119809, rs71374758, rs7229377, rs72655677, rs72789541, rs72836561, rs72920193, rs72924290, rs73109460, rs73487492, rs740006, rs7412, rs7500839, rs75354047, rs7570971, rs75938339, rs76380214, rs76978219, rs77027049, rs77053629, rs77407800, rs77960347, rs78353689, rs78689694*(rs6590199), rs78893833, rs79126702, rs7924036, rs79787170, rs79969098, rs8042174, rs9304381, rs952275, rs9896243, rs9987289 |  |
| LDL cholesterol | rs102275, rs10260606, rs10407439, rs10504255, rs10888896, rs10953298, rs11102964, rs111278137, rs112027066, rs11206517, rs112172017, rs112481437, rs112875651, rs1135062, rs114863007, rs115445558, rs115478735, rs11571787, rs11591147, rs1160983, rs11621792, rs11666995, rs11679386, rs11691986, rs117261169, rs11755689, rs118147862, rs12022410, rs12151108, rs12208357, rs12239737, rs12327715, rs1260326, rs12740374, rs12981555, rs13278097, rs1337247, rs139100794, rs139995984, rs140824606, rs142213240, rs144261139, rs144545816, rs145790091, rs1461729, rs146576912, rs148356565, rs148601586, rs149450221, rs1499279, rs1500188, rs150262789, rs150856817, rs150956718, rs151000110, rs151330717, rs1661174, rs17035665, rs17111503, rs17249001, rs174467, rs181765708, rs1836278, rs1883711, rs200149999, rs200730299, rs200756447, rs2015257, rs204480, rs204540, rs2065397, rs2570344, rs261290, rs2618566, rs2740488, rs28399637, rs28399664, rs28550459, rs2927439, rs2965157, rs2965167, rs2986164, rs34449399, rs35057129, rs35823804, rs35836101, rs36018387, rs372642577, rs377122620, rs3794695, rs3798167, rs3846662, rs405509, rs41279684, rs4149307, rs4263041, rs429358, rs4299376, rs4507059, rs472495, rs4803743, rs4803791, rs4804510, rs4927206, rs4927214, rs4970835, rs519113, rs525028, rs533617, rs553427, rs55714927, rs562338, rs56325564, rs58542926, rs59379014, rs59950280, rs60049679, rs6016505, rs602662, rs6073958, rs60960031, rs6102034, rs611060, rs62115552, rs62116311, rs62116947, rs62118464, rs6511721, rs651649, rs661278, rs662138, rs67616858, rs6859, rs6882345, rs693, rs6938647, rs7246666, rs7247937, rs7251161, rs72655677, rs72660539, rs72703210, rs72843670, rs72902594, rs73013176, rs73048351, rs7343130, rs73664373, rs740516, rs74607435, rs75237799, rs76186504, rs76670936, rs7700719, rs77241309, rs77278227, rs77303550, rs7750288, rs77542162, rs7773004, rs78477739, rs79220007, rs79429216, rs79915079, rs9276826, rs9749236, rs9973228 |  |
| Glutamine | rs1010931, rs10757283, rs10965245, rs11189300, rs115961201, rs11597826, rs11675484, rs117643180, rs11889865, rs12325419, rs1260326, rs1274961, rs1275519, rs12881221, rs13234131, rs13245286, rs13396296, rs141428740, rs147139486, rs17096421, rs1750768, rs1958393, rs1998848, rs2039098, rs2083180, rs2141371, rs2168101, rs2184968, rs2395779, rs2608977, rs2720586, rs28505379, rs28929474, rs340882, rs34139736, rs35261542, rs35823586, rs3736951, rs3748346, rs3794014, rs3988, rs4309084, rs4666051, rs56335308, rs58673065, rs62140395, rs62182473, rs6486195, rs6714780, rs7078003, rs7147721, rs7240148, rs73186030, rs738408, rs74873433, rs75662315, rs76123274, rs78431863, rs78484886, rs7925445, rs79687284, rs8074974, rs838737, rs9482770, rs9687846 |  |
| Creatinine | rs10008637, rs10062079, rs10224002, rs1050816, rs1065853, rs10821907, rs10903343, rs10926982, rs11070458, rs11072563, rs11130203, rs111366116, rs1136201, rs113775984, rs11564722, rs117268564, rs11786896, rs118156848, rs1268353, rs12722725, rs13015552, rs1317983, rs1381798, rs138576244, rs138861398, rs146625690, rs1527649, rs17050272, rs1717200, rs17513184, rs17786744, rs1781221, rs184561571, rs187355703, rs200800380, rs2119690, rs2271386, rs2279463, rs2338801, rs2455357*(rs4990988), rs2467853, rs267738, rs288460, rs288766, rs2894186, rs3122720, rs3130304, rs3131643, rs316034, rs34760583, rs35709439, rs35950587, rs3974479, rs408459, rs41269026, rs4293567, rs445537, rs4722500, rs4744712, rs478308, rs4946935, rs5030873, rs55733296, rs55842281, rs56019566, rs56068447, rs5750561, rs6088559, rs6127099, rs62435145, rs659437, rs690428, rs702634, rs715, rs7649167, rs7779671, rs7782653, rs77924615, rs7824557, rs7831557, rs80282103, rs8071709, rs8074363, rs81205, rs847146, rs848476, rs9269354, rs9272116, rs9373056, rs963837, rs964184, rs9868257, rs9905274, rs9927390, rs9989901 |  |
| Glycoproteins | rs10083777, rs10208743, rs1047891, rs10739076, rs10739110, rs10815428, rs10932472, rs10975159, rs111841620, rs112085022, rs113071241, rs113363823, rs114210411, rs114338044, rs114808769, rs115671969, rs116181500, rs116649057, rs117182696, rs117825338, rs118096183, rs11889427, rs11901164, rs11902281, rs12338205, rs12694211, rs13007126, rs13009426, rs13026255, rs13298772, rs139728050, rs140360589, rs141705179, rs142033808, rs142807069, rs142843919, rs143238289, rs143955150, rs144035272, rs146169358, rs146851439, rs1470498, rs148041738, rs149877929, rs17748227, rs181052754, rs1864353, rs186757218, rs190126699, rs193220212, rs1992855, rs2169387, rs2282162, rs2287598, rs2302908, rs2371011, rs2542929, rs34023256, rs34454501, rs41272663, rs4673618, rs55663096, rs56212035, rs62177972, rs62201970, rs62203712, rs62203740, rs62203749, rs62568995, rs6435548, rs67225130, rs6752652, rs6756888, rs7012637, rs72930538, rs72935806, rs72949273, rs7425448, rs74753815, rs7570368, rs75764781, rs7582376, rs77039478, rs77598098, rs78600267, rs78815845 |  |
| Pyruvate | rs10751912, rs111941366, rs11251684, rs113312468, rs114192724, rs116789570, rs117921965, rs12134456, rs1260326, rs12923645, rs13024014, rs1354034, rs138373837, rs139531336, rs1427407, rs17285851, rs17753893, rs1800526, rs188469011, rs1920980, rs2073578, rs2388595, rs25190938*(rs115478735), rs2540916, rs2667735, rs2729389, rs371269975, rs3761393, rs41265007, rs4844599, rs4972840, rs60757417, rs62220377, rs6592965, rs6602022, rs68149176, rs6993770, rs7342119, rs74585025, rs7584089, rs760077, rs7901552, rs79037747, rs8049831, rs8053808, rs8061221, rs9952972, rs9985166 |  |
| Valine | rs10018448, rs112440541, rs1128249, rs114556806, rs11652753, rs117048185, rs117169693, rs11733230, rs117643180, rs12325419, rs1260326, rs12641788, rs12974412, rs139152498, rs143891741, rs145585828, rs1471740, rs149144856, rs17096421, rs17206700, rs185220479, rs189652270, rs193062548, rs201108974, rs202049562, rs222847, rs2238732, rs2274815, rs2422358, rs2725250, rs2869113, rs2943652, rs34849690, rs34894639, rs35230038, rs35332782, rs4671637, rs4985545, rs61587941, rs62309960, rs6499295, rs6509405, rs6821073, rs6941263, rs72839770, rs72879308, rs78169150, rs78948284, rs78948711, rs79606828, rs8071084, rs863277, rs9307048 |  |
| Histidine | rs1047891, rs10935742, rs11621792, rs117046983, rs117591301, rs12140995, rs13019835, rs13021675, rs138570705, rs143473440, rs150844304, rs187633915, rs1883711, rs1958029, rs1958393, rs1998848, rs2168101, rs2272662, rs2614813, rs28487964, rs34004251, rs3748348, rs4982399, rs6029178, rs60660268, rs709932, rs7588072, rs780094, rs78217698, rs9646847 |  |
| Glucose | rs10195252, rs114011597, rs114764002, rs11720108, rs12244654, rs12702068, rs13169290, rs13266634, rs1333051, rs13430620, rs16856252, rs1974, rs2284769, rs28675349, rs34872471, rs3778321, rs4325403, rs477224, rs55881843, rs560887, rs56100844, rs566372607*(rs11251714), rs62065453, rs6433088, rs6602022, rs6787208, rs6804915, rs6972516, rs7101069, rs730497, rs74648148, rs75739013, rs77317718, rs79687284 |  |
| Free cholesterol | rs10424477, rs1077835, rs10888908, rs113105517, rs113445611, rs113531395, rs115445558, rs115534052, rs11591147, rs11604424, rs116264584, rs116302332, rs12043403, rs13392272, rs142130958, rs144064722, rs146568567, rs174418, rs1825955, rs182695896, rs185567543, rs190934192, rs3005923, rs429358, rs4426495, rs4609471, rs4635554, rs479084, rs56028521, rs565436, rs61770425, rs629301, rs6511721, rs72664202, rs726815, rs7412, rs75679663, rs77021821, rs79225634 |  |
| 3-Hydroxybutyrate | rs10481445, rs10864726, rs117643180, rs2070666, rs2419604, rs2575876, rs2645430, rs28929474, rs2976866, rs35853021, rs4149307, rs429358, rs4788460, rs56177707, rs585188, rs588136, rs6889983, rs72702354, rs77234835, rs7831557, rs964184, rs9930957, rs9987289 |  |
| Butyrylcarnitine | rs1171617, rs2469211, rs274567 |  |
| Acetate | rs116013021, rs12005199, rs1260326, rs138319634, rs139097404, rs142237051, rs145679432, rs185281343, rs2424699, rs3859660, rs4813543, rs4988235, rs6036912, rs6050281, rs6057240, rs6106989, rs6138465, rs78845105, rs8123210 | rs12005199, rs1260326, rs138319634, rs139097404, rs145679432, rs185281343, rs2424699, rs3184504, rs3859660, rs4813543, rs4988235, rs6050281, rs6057240, rs6106989, rs6138465, rs78845105 |
| Carnitine | rs11620955, rs11620973, rs1171618, rs12356193, rs12709393, rs13182512, rs1466788, rs2114713, rs2279014, rs2396004, rs2492788, rs3736438, rs419291, rs4860022, rs6479648, rs6862024, rs7098081, rs735315, rs7737937, rs9842133 |  |
| Tryptophan | rs1016522, rs13122250, rs1373962, rs1559063, rs2111118, rs284191, rs38271, rs4306882, rs4615256, rs4958379, rs603446, rs6480970, rs6935961, rs710580, rs7463805, rs7584842, rs9511152, rs972459 |  |
| Isoleucine | rs10018448, rs10184004, rs117169693, rs117459045, rs117643180, rs12325419, rs1260326, rs139152498, rs141413744, rs185220479, rs189652270, rs2869113, rs2941456, rs35332782, rs371646176, rs545587, rs56011312, rs6840723, rs79983034 |  |
| Acetone | rs11216157, rs115701438, rs11745373, rs117643180, rs117651719, rs117810762, rs1536826, rs2238691, rs2246634, rs2582783, rs318020, rs35953019, rs62360541, rs6889983, rs72757003, rs72864795, rs9590637 |  |
| X-12092 | rs1061134, rs1061135, rs10748725, rs10883057, rs11189692, rs11595763, rs12415538, rs12780272, rs1365, rs17109581, rs1889459, rs4917817, rs4919221, rs542386, rs7096654, rs754586, rs8101881, rs942800, rs942812 |  |
| Lactate | rs10758666, rs12203816, rs12273368, rs1473698, rs3780359, rs385893, rs390387, rs4665972, rs572141340(rs35129023), rs59273177, rs61835136, rs6602022, rs6993770, rs7101069, rs9389268 |  |
| X-12063 | rs10242455, rs11762932, rs13230554, rs296396, rs4917639, rs6465760, rs6544713, rs7795644, rs7797978, rs952319 |  |
| X-11593--O-methylascorbate | rs10412803, rs11089325, rs16982844, rs2686184, rs3804043, rs438798, rs4597638, rs4680, rs7290062, rs7707010, rs887200, rs9318225, rs9606212 |  |
| X-12798 | rs10945673, rs1171615, rs1171650, rs13220412, rs1367211, rs316020, rs316032, rs316174, rs3799344, rs532440, rs628031, rs791174, rs9456505 |  |
| Succinylcarnitine | rs10988217, rs11637751, rs12899230, rs1472631, rs17806888, rs2686513, rs2729786, rs2729816, rs6703518, rs8060756, rs901273, rs924135 |  |
| Glutaroyl carnitine | rs11085835, rs12597880, rs12927959, rs13375749, rs17641971, rs1981524, rs2291449, rs246234, rs715, rs8012, rs8056893, rs896388 |  |
| Leucine | rs10923016, rs1440581, rs1871053, rs2713737, rs6735596, rs6737109, rs6820011, rs6820313, rs7584842, rs7647029, rs7740614, rs9541214 |  |
| Albumin | rs114822153, rs115534052, rs116302332, rs116329419, rs12500991, rs144064722, rs17270586, rs182695896, rs185567543, rs28436733, rs77021821, rs78906559 |  |
| N-acetylornithine | rs1036113, rs11679923, rs13415017, rs1653259, rs17348756, rs17349853, rs1881244, rs2043099, rs6546815, rs7573275 |  |
| X-11793--oxidized bilirubin | rs10175949, rs1115381, rs11563251, rs11683356, rs12479240, rs28899194, rs6760588, rs838705, rs887829 |  |
| X-12728 | rs12326998, rs12769714, rs12967460, rs13094553, rs16896783, rs1912320, rs2830801, rs8089543, rs9566930 |  |
| Biliverdin | rs10170160, rs17864661, rs2219067, rs28900371, rs6717546, rs7608713, rs838705, rs887829, rs988344 |  |
| X-11787 | rs10206899, rs17009399, rs17110031, rs17349853, rs17725971, rs1891071, rs1934963, rs2298037, rs869927 |  |
| Glycine | rs10932349, rs12328639, rs13011429, rs13021675, rs13401425, rs16845044, rs1990797, rs2719966, rs715 |  |
| Hexanoylcarnitine | rs11161521, rs1171615, rs17304141, rs2070630, rs272869, rs5745515, rs5745542, rs721204, rs7534288 |  |
| X-03056--N-[3-(2-Oxopyrrolidin-1-yl)propyl]acetamide | rs11161521, rs1171615, rs17304141, rs2070630, rs272869, rs5745515, rs5745542, rs721204, rs7534288 |  |
| Bilirubin (Z, Z) | rs12479240, rs28900385, rs3771342, rs3796092, rs6717546, rs6760588, rs838718, rs887829 |  |
| Androsterone sulfate | rs10278040, rs13222543, rs182420, rs2141160, rs4236541, rs474229, rs6465737, rs7778571 |  |
| X-11530 | rs17863762, rs2018609, rs28900385, rs4047189, rs6723936, rs7608713, rs838718, rs887829 |  |
| Octanoylcarnitine | rs17304141, rs5745515, rs5745542, rs6849801, rs721204, rs7552404, rs8396, rs924135 |  |
| Isobutyrylcarnitine | rs12202350, rs2404602, rs3798167, rs4886484, rs648253, rs662138, rs9456496, rs9457843 |  |
| X-13431--nonanoylcarnitine | rs12566232, rs1509821, rs2302539, rs2539861, rs3738934, rs4672574, rs992029 |  |
| X-12510--2-aminooctanoic acid | rs1165209, rs1318987, rs13538, rs1466245, rs17009399, rs17348756, rs6710726 |  |
| X-11440 | rs10418834 rs11670965 rs2932766 rs296381 rs3732218 rs4715354 rs4802397 |  |
| X-11442 | rs2219067, rs28899194, rs3796092, rs4148324, rs6723936, rs7608713, rs838705 |  |
| Epiandrosterone sulfate | rs10228787, rs11981478, rs13222543, rs2141160, rs474229, rs6465737, rs7778571 |  |
| Proline | rs11802885 rs2518802 rs5746636 rs5747934 rs715544 rs9605907 rs9680846 |  |
| Bilirubin (E, E) | rs28900371, rs3796092, rs6723936, rs730673, rs7608713, rs838718, rs887829 |  |
| N-acetylglycine | rs12328639 rs12468557 rs13021675 rs1367053 rs1990797 rs715 rs7948073 |  |
| Isovalerylcarnitine | rs11950562, rs17672041, rs2291449, rs2662314, rs4706020, rs6596022, rs9635324 |  |
| 1, 5-anhydroglucitol (1, 5-AG) | rs12465802, rs3800993, rs4954573, rs666614, rs6736412, rs7570971, rs9756306 |  |
| X-11444 | rs10199511, rs1346644, rs2273664, rs3820915, rs559555, rs6735065, rs7604035 |  |
| Betaine | rs16876394, rs185077, rs2851391, rs715, rs9982015 |  |
| Citrate | rs1509123, rs170149, rs6507399, rs807670, rs835154, rs868395, rs906749 |  |
| X-11441 | rs2219067, rs28900384, rs3796092, rs4148324, rs6717546, rs7608713 |  |
| 4-androsten-3beta, 17beta-diol disulfate 1 | rs11669552, rs11761528, rs296396, rs4149452, rs4802397, rs7259671 |  |
| X-08402 | rs10140289, rs1271565, rs12881815, rs4814176, rs7157785, rs8022917 |  |
| Mannose | rs1260326, rs2141371, rs6547811, rs7349418, rs7583698, rs937813 |  |
| 4-acetamidobutanoate | rs1041983, rs13271577, rs2029830, rs721399, rs7228099, rs9657488 |  |
| 5alpha-androstan-3beta, 17beta-diol disulfate | rs10267212, rs10278040, rs13222543, rs2547231, rs4149452 |  |
| X-12244--N-acetylcarnosine | rs2484984, rs282115, rs4148421, rs6804368, rs7775554, rs8002180 |  |
| Kynurenine | rs10085935, rs16924894, rs750950, rs8051149 |  |
| 3-dehydrocarnitine | rs10070034, rs17171585, rs2291428, rs273913, rs316019, rs7737937 |  |
| X-11261 | rs10070034, rs17171585, rs2291428, rs273913, rs316019, rs7737937 |  |
| X-12093 | rs10469966, rs2070581, rs4488133, rs4919209,  rs7600291 |  |
| Bradykinin, des-arg(9) | rs12511874, rs2731672, rs4253243, rs4253311,  rs5030062 |  |
| Urate | rs10939663, rs11731100, rs11737601,  rs4697910, rs938554 |  |
| 1-arachidonoylglycerophosphocholine | rs1692120, rs174535, rs3738544 rs7104849, rs748196 |  |
| Arachidonate (20:4n6) | rs174548, rs174602, rs17764324, rs412334,  rs526126 |  |
| Cis-4-decenoyl carnitine | rs11161521, rs12143716, rs17304141, rs721204, rs8396 |  |
| Decanoylcarnitine | rs11722868, rs17304141, rs2062541, rs7552404, rs8396 |  |
| X-12696 | rs12465802, rs13005339, rs3800993, rs7568884, rs7570971 |  |
| 1-arachidonoylglycerophosphoinositol | rs174578, rs8736, rs9326061 |  |
| Propionylcarnitine | rs10128501, rs12356193, rs2715311, rs662138, rs7727544 |  |
| Pyroglutamine | rs1600760, rs17279437, rs2159892, rs715 |  |
| X-03094 | rs1260326, rs2187375, rs6679002, rs964184, rs9855331 |  |
| X-11469 | rs10509673, rs11188148, rs11754288, rs2071426 |  |
| Gamma-glutamyltyrosine | rs12192718, rs1512342, rs875740, rs956966, rs9940990 |  |
| Bilirubin (E, Z or Z, E) | rs28898590, rs4281899, rs6723936, rs887829 |  |
| 10-undecenoate (11:1n1) | rs2494248, rs6077678, rs7556052, rs9333029 |  |
| Asparagine | rs2011069, rs3017098, rs8008020 |  |
| X-12556 | rs2235649, rs4129624, rs4675874, rs7590032 |  |
| X-08988 | rs10938307, rs13021675, rs715 |  |
| Tryptophan betaine | rs13184973, rs2405522, rs2548993, rs7733814 |  |
| X-02269 | rs11188148, rs1165196, rs2071426 |  |
| X-12844 | rs11666603, rs17614939, rs2035647, rs430200 |  |
| Tyrosine | rs12728678, rs172650, rs174408, rs9400467 |  |
| Citrulline | rs10164524, rs1509820, rs1935815, rs682103 |  |
| X-14205--alpha-glutamyltyrosine | rs13105073, rs2881913, rs4351 |  |
| X-11792 | rs2731672, rs4253252, rs698078 |  |
| X-13435 | rs2062541, rs6597030, rs9392556 |  |
| Serine | rs1163251, rs4947534, rs715 |  |
| X-10510 | rs3762660, rs4814176, rs4902243 |  |
| Alpha-hydroxyisovalerate | rs12141041, rs2403254, rs893971 |  |
| X-12850 | rs11019976, rs2547231, rs279941 |  |
| Dihomo-linolenate (20:3n3 or n6) | rs174455, rs6498540, rs968567 |  |
| X-11315 | rs1809148, rs3761097, rs4327428 |  |
| X-09789 | rs11185516, rs556339, rs7642243 |  |
| 3-methyl-2-oxovalerate | rs11084396, rs1260326, rs1440581 |  |
| Uridine | rs131794, rs2686796, rs532545 |  |
| Erythronate | rs10263766, rs2391217, rs4687717 |  |
| HWESASXX | rs2007084, rs7535263, rs893616 |  |
| X-11204 | rs10468017, rs2935461, rs8033940 |  |

Abbreviation: MR, Mendelian randomization; SNP, single nucleotide polymorphism.

* SNPs (proxy SNPs) that were not available in breast cancer dataset.

**Table S3. 679 diseases incorporated in the Phe-MR analysis.**

| **PheCode** | **Phenotype Description** | **Disease Category** | **Number of cases** | **Number of controls** | **Number of excluded controls** |
| --- | --- | --- | --- | --- | --- |
| 401 | Hypertension | circulatory system | 77,977 | 330,366 | 618 |
| 401.1 | Essential hypertension | circulatory system | 77,723 | 330,366 | 872 |
| 550 | Abdominal hernia | digestive | 47,344 | 361,617 | 0 |
| 785 | Abdominal pain | symptoms | 41,316 | 367,645 | 0 |
| 716 | Other arthropathies | musculoskeletal | 38,715 | 365,819 | 4,427 |
| 716.9 | Arthropathy NOS | musculoskeletal | 37,043 | 365,819 | 6,099 |
| 272 | Disorders of lipid metabolism | endocrine/metabolic | 35,927 | 373,034 | 0 |
| 530 | Diseases of esophagus | digestive | 35,852 | 369,275 | 3,834 |
| 272.1 | Hyperlipidemia | endocrine/metabolic | 35,844 | 373,034 | 83 |
| 272.11 | Hypercholesterolemia | endocrine/metabolic | 33,242 | 373,034 | 2,685 |
| 530.1 | Esophagitis, GERD and related diseases | digestive | 32,108 | 369,275 | 7,578 |
| 418 | Nonspecific chest pain | circulatory system | 31,429 | 377,532 | 0 |
| 411 | Ischemic Heart Disease | circulatory system | 31,355 | 377,103 | 503 |
| 535 | Gastritis and duodenitis | digestive | 28,941 | 378,124 | 1,896 |
| 306 | Other mental disorder | mental disorders | 28,791 | 365,476 | 14,694 |
| 740 | Osteoarthrosis | musculoskeletal | 28,439 | 380,522 | 0 |
| 562 | Diverticulosis and diverticulitis | digestive | 27,311 | 334,783 | 46,867 |
| 562.1 | Diverticulosis | digestive | 27,268 | 334,783 | 46,910 |
| 550.2 | Diaphragmatic hernia | digestive | 27,126 | 361,617 | 20,218 |
| 495 | Asthma | respiratory | 26,332 | 375,505 | 7,124 |
| 427 | Cardiac dysrhythmias | circulatory system | 24,681 | 380,919 | 3,361 |
| 599 | Other symptoms/disorders or the urinary system | genitourinary | 24,031 | 384,930 | 0 |
| 455 | Hemorrhoids | circulatory system | 23,896 | 369,592 | 15,473 |
| 564 | Functional digestive disorders | digestive | 22,138 | 334,783 | 52,040 |
| 197 | Chemotherapy | neoplasms | 21,798 | 370,604 | 16,559 |
| 578 | Gastrointestinal hemorrhage | digestive | 21,137 | 385,157 | 2,667 |
| 366 | Cataract | sense organs | 20,352 | 388,609 | 0 |
| 208 | Benign neoplasm of colon | neoplasms | 20,204 | 386,011 | 2,746 |
| 250 | Diabetes mellitus | endocrine/metabolic | 20,203 | 388,756 | 2 |
| 411.4 | Coronary atherosclerosis | circulatory system | 20,023 | 377,103 | 11,835 |
| 318 | Tobacco use disorder | mental disorders | 19,780 | 379,355 | 9,826 |
| 250.2 | Type 2 diabetes | endocrine/metabolic | 18,945 | 388,756 | 1,260 |
| 960 | Poisoning by antibiotics | injuries & poisonings | 18,430 | 381,797 | 8,734 |
| 740.1 | Osteoarthritis; localized | musculoskeletal | 17,691 | 380,522 | 10,748 |
| 195 | Cancer, suspected or other | neoplasms | 16,725 | 370,604 | 21,632 |
| 459 | Other disorders of circulatory system | circulatory system | 16,544 | 387,905 | 4,512 |
| 593 | Hematuria | genitourinary | 16,409 | 379,936 | 12,616 |
| 1019 | Other ill-defined and unknown causes of morbidity and mortality | symptoms | 16,398 | 392,563 | 0 |
| 459.9 | Circulatory disease NEC | circulatory system | 16,366 | 387,905 | 4,690 |
| 574 | Cholelithiasis and cholecystitis | digestive | 16,225 | 391,307 | 1,429 |
| 411.3 | Angina pectoris | circulatory system | 16,175 | 377,103 | 15,683 |
| 960.2 | Allergy/adverse effect of penicillin | injuries & poisonings | 16,090 | 381,797 | 11,074 |
| 550.1 | Inguinal hernia | digestive | 15,995 | 361,617 | 31,349 |
| 195.1 | Malignant neoplasm, other | neoplasms | 15,979 | 370,604 | 22,378 |
| 561 | Symptoms involving digestive system | digestive | 15,977 | 334,783 | 58,201 |
| 716.2 | Unspecified monoarthritis | musculoskeletal | 15,790 | 365,819 | 27,352 |
| 558 | Noninfectious gastroenteritis | digestive | 15,747 | 334,783 | 58,431 |
| 835 | Internal derangement of knee | injuries & poisonings | 15,430 | 391,457 | 2,074 |
| 564.9 | Personal history of diseases of digestive system | digestive | 15,392 | 334,783 | 58,786 |
| 565 | Anal and rectal conditions | digestive | 14,997 | 387,338 | 6,626 |
| 726 | Peripheral enthesopathies and allied syndromes | musculoskeletal | 14,983 | 378,711 | 15,267 |
| 411.8 | Other chronic ischemic heart disease, unspecified | circulatory system | 14,921 | 377,103 | 16,937 |
| 244 | Hypothyroidism | endocrine/metabolic | 14,871 | 391,429 | 2,661 |
| 427.2 | Atrial fibrillation and flutter | circulatory system | 14,820 | 380,919 | 13,222 |
| 530.11 | GERD | digestive | 14,223 | 369,275 | 25,463 |
| 244.4 | Hypothyroidism NOS | endocrine/metabolic | 14,171 | 391,429 | 3,361 |
| 574.1 | Cholelithiasis | digestive | 13,777 | 391,307 | 3,877 |
| 172 | Skin cancer | neoplasms | 13,752 | 395,071 | 138 |
| 578.8 | Hemorrhage of rectum and anus | digestive | 13,222 | 385,157 | 10,582 |
| 317 | Alcohol-related disorders | mental disorders | 12,922 | 379,355 | 16,684 |
| 174 | Breast cancer | neoplasms | 12,898 | 388,549 | 7,514 |
| 351 | Other peripheral nerve disorders | neurological | 12,592 | 394,067 | 2,302 |
| 296 | Mood disorders | mental disorders | 12,560 | 365,476 | 30,925 |
| 591 | Urinary tract infection | genitourinary | 12,491 | 379,936 | 16,534 |
| 740.9 | Osteoarthrosis NOS | musculoskeletal | 12,436 | 380,522 | 16,003 |
| 285 | Other anemias | hematopoietic | 12,256 | 390,026 | 6,679 |
| 41 | Bacterial infection NOS | infectious diseases | 12,187 | 393,897 | 2,877 |
| 454 | Varicose veins | circulatory system | 12,172 | 369,592 | 27,197 |
| 296.2 | Depression | mental disorders | 11,901 | 365,476 | 31,584 |
| 789 | Nausea and vomiting | symptoms | 11,706 | 397,255 | 0 |
| 411.2 | Myocardial infarction | circulatory system | 11,703 | 377,103 | 20,155 |
| 454.1 | Varicose veins of lower extremity | circulatory system | 11,697 | 369,592 | 27,672 |
| 760 | Back pain | symptoms | 11,274 | 397,687 | 0 |
| 172.2 | Other non-epithelial cancer of skin | neoplasms | 11,149 | 395,071 | 2,741 |
| 278 | Overweight, obesity and other hyperalimentation | endocrine/metabolic | 10,968 | 397,993 | 0 |
| 278.1 | Obesity | endocrine/metabolic | 10,799 | 397,993 | 169 |
| 530.14 | Reflux esophagitis | digestive | 10,551 | 369,275 | 29,135 |
| 496 | Chronic airway obstruction | respiratory | 10,502 | 375,505 | 22,954 |
| 563 | Constipation | digestive | 10,442 | 334,783 | 63,736 |
| 480 | Pneumonia | respiratory | 10,059 | 398,538 | 364 |
| 596 | Other disorders of bladder | genitourinary | 9,933 | 394,699 | 4,329 |
| 735 | Acquired foot deformities | musculoskeletal | 9,865 | 394,914 | 4,182 |
| 726.1 | Enthesopathy | musculoskeletal | 9,668 | 378,711 | 20,582 |
| 198 | Secondary malignant neoplasm | neoplasms | 9,483 | 370,604 | 28,874 |
| 519 | Other diseases of respiratory system, not elsewhere classified | respiratory | 9,436 | 399,525 | 0 |
| 722 | Intervertebral disc disorders | musculoskeletal | 9,241 | 391,917 | 7,803 |
| 788 | Syncope and collapse | symptoms | 9,163 | 399,798 | 0 |
| 1011 | Complications of surgical and medical procedures | injuries & poisonings | 9,140 | 399,821 | 0 |
| 512 | Other symptoms of respiratory system | respiratory | 9,128 | 399,833 | 0 |
| 740.11 | Osteoarthrosis, localized, primary | musculoskeletal | 9,069 | 380,522 | 19,370 |
| 8 | Intestinal infection | infectious diseases | 8,991 | 399,970 | 0 |
| 317.1 | Alcoholism | mental disorders | 8,968 | 379,355 | 20,638 |
| 706 | Diseases of sebaceous glands | dermatologic | 8,948 | 399,255 | 758 |
| 706.2 | Sebaceous cyst | dermatologic | 8,876 | 399,255 | 830 |
| 599.4 | Urinary incontinence | genitourinary | 8,856 | 384,930 | 15,175 |
| 519.8 | Other diseases of respiratory system, NEC | respiratory | 8,844 | 399,525 | 592 |
| 433 | Cerebrovascular disease | circulatory system | 8,742 | 399,017 | 1,202 |
| 803 | Fracture of upper limb | injuries & poisonings | 8,521 | 387,765 | 12,675 |
| 1009 | Injury, NOS | injuries & poisonings | 8,440 | 400,521 | 0 |
| 366.2 | Senile cataract | sense organs | 8,369 | 388,609 | 11,983 |
| 535.8 | Other specified gastritis | digestive | 8,147 | 378,124 | 22,690 |
| 745 | Pain in joint | musculoskeletal | 8,037 | 400,924 | 0 |
| 721 | Spondylosis and allied disorders | musculoskeletal | 7,930 | 391,917 | 9,114 |
| 339 | Other headache syndromes | neurological | 7,891 | 398,780 | 2,290 |
| 280 | Iron deficiency anemias | hematopoietic | 7,787 | 390,026 | 11,148 |
| 216 | Benign neoplasm of skin | neoplasms | 7,722 | 400,618 | 621 |
| 743 | Osteoporosis, osteopenia and pathological fracture | musculoskeletal | 7,682 | 401,279 | 0 |
| 535.6 | Duodenitis | digestive | 7,655 | 378,124 | 23,182 |
| 727 | Other disorders of synovium, tendon, and bursa | musculoskeletal | 7,629 | 378,711 | 22,621 |
| 276 | Disorders of fluid, electrolyte, and acid-base balance | endocrine/metabolic | 7,455 | 401,506 | 0 |
| 681 | Superficial cellulitis and abscess | dermatologic | 7,451 | 397,635 | 3,875 |
| 531 | Peptic ulcer (excl. esophageal) | digestive | 7,436 | 401,525 | 0 |
| 280.1 | Iron deficiency anemias, unspecified or not due to blood loss | hematopoietic | 7,414 | 390,026 | 11,521 |
| 565.1 | Anal and rectal polyp | digestive | 7,408 | 387,338 | 14,215 |
| 800 | Fracture of lower limb | injuries & poisonings | 7,251 | 387,765 | 13,945 |
| 585 | Renal failure | genitourinary | 6,985 | 397,602 | 4,374 |
| 426 | Cardiac conduction disorders | circulatory system | 6,959 | 380,919 | 21,083 |
| 300 | Anxiety disorders | mental disorders | 6,939 | 365,476 | 36,546 |
| 965 | Poisoning by analgesics, antipyretics, and antirheumatics | injuries & poisonings | 6,910 | 381,797 | 20,254 |
| 599.2 | Retention of urine | genitourinary | 6,755 | 384,930 | 17,276 |
| 480.1 | Bacterial pneumonia | respiratory | 6,710 | 398,538 | 3,713 |
| 735.3 | Hallux valgus (Bunion) | musculoskeletal | 6,699 | 394,914 | 7,348 |
| 594 | Urinary calculus | genitourinary | 6,643 | 401,005 | 1,313 |
| 790 | Nonspecific findings on examination of blood | symptoms | 6,619 | 402,342 | 0 |
| 743.1 | Osteoporosis | musculoskeletal | 6,484 | 401,279 | 1,198 |
| 532 | Dysphagia | digestive | 6,482 | 369,275 | 33,204 |
| 507 | Pleurisy; pleural effusion | respiratory | 6,448 | 397,411 | 5,102 |
| 790.6 | Other abnormal blood chemistry | symptoms | 6,413 | 402,342 | 206 |
| 300.1 | Anxiety disorder | mental disorders | 6,375 | 365,476 | 37,110 |
| 773 | Pain in limb | symptoms | 6,327 | 402,634 | 0 |
| 214 | Lipoma | neoplasms | 6,271 | 401,613 | 1,077 |
| 729 | Other disorders of soft tissues | musculoskeletal | 6,170 | 378,711 | 24,080 |
| 386 | Vertiginous syndromes and other disorders of vestibular system | sense organs | 6,134 | 402,827 | 0 |
| 1010 | Other tests | symptoms | 5,972 | 402,989 | 0 |
| 480.11 | Pneumococcal pneumonia | respiratory | 5,951 | 398,538 | 4,472 |
| 512.7 | Shortness of breath | respiratory | 5,884 | 399,833 | 3,244 |
| 573 | Other disorders of liver | digestive | 5,847 | 400,055 | 3,059 |
| 458 | Hypotension | circulatory system | 5,827 | 387,905 | 15,229 |
| 689 | Disorder of skin and subcutaneous tissue NOS | dermatologic | 5,782 | 403,179 | 0 |
| 374 | Other disorders of eyelids | sense organs | 5,726 | 399,306 | 3,929 |
| 743.11 | Osteoporosis NOS | musculoskeletal | 5,622 | 401,279 | 2,060 |
| 159 | Malignant neoplasm of other and ill-defined sites within the digestive organs and peritoneum | neoplasms | 5,584 | 393,372 | 10,005 |
| 687 | Symptoms affecting skin | dermatologic | 5,554 | 403,407 | 0 |
| 564.1 | Irritable Bowel Syndrome | digestive | 5,548 | 334,783 | 68,630 |
| 681.5 | Cellulitis and abscess of leg, except foot | dermatologic | 5,547 | 397,635 | 5,779 |
| 681.3 | Cellulitis and abscess of arm/hand | dermatologic | 5,539 | 397,635 | 5,787 |
| 702 | Degenerative skin conditions and other dermatoses | dermatologic | 5,522 | 398,746 | 4,693 |
| 1002 | Symptoms concerning nutrition, metabolism, and development | symptoms | 5,512 | 403,449 | 0 |
| 681.6 | Cellulitis and abscess of foot, toe | dermatologic | 5,502 | 397,635 | 5,824 |
| 771 | Musculoskeletal symptoms referable to limbs | symptoms | 5,493 | 403,468 | 0 |
| 574.12 | Cholelithiasis with other cholecystitis | digestive | 5,472 | 391,307 | 12,182 |
| 428 | Congestive heart failure; nonhypertensive | circulatory system | 5,415 | 402,834 | 712 |
| 198.1 | Secondary malignancy of lymph nodes | neoplasms | 5,379 | 370,604 | 32,978 |
| 704 | Diseases of hair and hair follicles | dermatologic | 5,344 | 402,357 | 1,260 |
| 850 | Hemorrhage or hematoma complicating a procedure | injuries & poisonings | 5,329 | 394,929 | 8,703 |
| 211 | Benign neoplasm of other parts of digestive system | neoplasms | 5,280 | 395,301 | 8,380 |
| 803.2 | Fracture of radius and ulna | injuries & poisonings | 5,246 | 387,765 | 15,950 |
| 530.12 | Ulcer of esophagus | digestive | 5,243 | 369,275 | 34,443 |
| 327 | Sleep disorders | neurological | 5,238 | 403,723 | 0 |
| 578.9 | Hemorrhage of gastrointestinal tract | digestive | 5,229 | 385,157 | 18,575 |
| 411.1 | Unstable angina (intermediate coronary syndrome) | circulatory system | 5,181 | 377,103 | 26,677 |
| 735.2 | Acquired toe deformities | musculoskeletal | 5,144 | 394,914 | 8,903 |
| 345 | Epilepsy, recurrent seizures, convulsions | neurological | 5,087 | 395,209 | 8,665 |
| 721.1 | Spondylosis without myelopathy | musculoskeletal | 5,077 | 391,917 | 11,967 |
| 470 | Septal Deviations/Turbinate Hypertrophy | respiratory | 4,939 | 390,045 | 13,977 |
| 394 | Rheumatic disease of the heart valves | circulatory system | 4,895 | 402,421 | 1,645 |
| 714 | Rheumatoid arthritis and other inflammatory polyarthropathies | musculoskeletal | 4,879 | 365,085 | 38,997 |
| 701 | Other hypertrophic and atrophic conditions of skin | dermatologic | 4,804 | 403,875 | 282 |
| 771.1 | Swelling of limb | symptoms | 4,663 | 403,468 | 830 |
| 292 | Neurological disorders | mental disorders | 4,655 | 402,383 | 1,923 |
| 214.1 | Lipoma of skin and subcutaneous tissue | neoplasms | 4,611 | 401,613 | 2,737 |
| 386.9 | Dizziness and giddiness (Light-headedness and vertigo) | sense organs | 4,611 | 402,827 | 1,523 |
| 153 | Colorectal cancer | neoplasms | 4,562 | 382,756 | 21,643 |
| 555 | Inflammatory bowel disease and other gastroenteritis and colitis | digestive | 4,528 | 334,783 | 69,650 |
| 585.1 | Acute renal failure | genitourinary | 4,521 | 397,602 | 6,838 |
| 686 | Other local infections of skin and subcutaneous tissue | dermatologic | 4,520 | 397,635 | 6,806 |
| 80 | Postoperative infection | infectious diseases | 4,489 | 402,343 | 2,129 |
| 728 | Disorders of muscle, ligament, and fascia | musculoskeletal | 4,488 | 378,711 | 25,762 |
| 327.3 | Sleep apnea | neurological | 4,471 | 403,723 | 767 |
| 365 | Glaucoma | sense organs | 4,462 | 397,761 | 6,738 |
| 722.9 | Other and unspecified disc disorder | musculoskeletal | 4,434 | 391,917 | 12,610 |
| 714.1 | Rheumatoid arthritis | musculoskeletal | 4,412 | 365,085 | 39,464 |
| 915 | Superficial injury without mention of infection | injuries & poisonings | 4,289 | 403,595 | 1,077 |
| 428.2 | Heart failure NOS | circulatory system | 4,269 | 402,834 | 1,858 |
| 415 | Pulmonary heart disease | circulatory system | 4,257 | 402,375 | 2,329 |
| 389 | Hearing loss | sense organs | 4,256 | 404,562 | 143 |
| 395 | Heart valve disorders | circulatory system | 4,239 | 402,421 | 2,301 |
| 1015 | Effects of other external causes | symptoms | 4,230 | 404,731 | 0 |
| 871 | Open wounds of extremities | injuries & poisonings | 4,219 | 400,426 | 4,316 |
| 569 | Other disorders of intestine | digestive | 4,200 | 387,338 | 17,423 |
| 289 | Other diseases of blood and blood-forming organs | hematopoietic | 4,177 | 401,375 | 3,409 |
| 189 | Cancer of urinary organs (incl. kidney and bladder) | neoplasms | 4,165 | 404,796 | 0 |
| 433.2 | Occlusion of cerebral arteries | circulatory system | 4,134 | 399,017 | 5,810 |
| 276.1 | Electrolyte imbalance | endocrine/metabolic | 4,123 | 401,506 | 3,332 |
| 531.2 | Gastric ulcer | digestive | 4,109 | 401,525 | 3,327 |
| 479 | Other upper respiratory disease | respiratory | 4,101 | 390,045 | 14,815 |
| 597 | Other disorders of urethra and urinary tract | genitourinary | 4,069 | 394,699 | 10,193 |
| 599.5 | Frequency of urination and polyuria | genitourinary | 4,037 | 384,930 | 19,994 |
| 38 | Septicemia | infectious diseases | 4,005 | 393,897 | 11,059 |
| 560 | Intestinal obstruction without mention of hernia | digestive | 3,994 | 334,783 | 70,184 |
| 783 | Fever of unknown origin | symptoms | 3,940 | 405,021 | 0 |
| 528 | Diseases of the oral soft tissues, excluding lesions specific for gingiva and tongue | digestive | 3,939 | 403,323 | 1,699 |
| 443 | Peripheral vascular disease | circulatory system | 3,927 | 400,595 | 4,439 |
| 379 | Other disorders of eye | sense organs | 3,910 | 401,245 | 3,806 |
| 451 | Phlebitis and thrombophlebitis | circulatory system | 3,900 | 369,592 | 35,469 |
| 575 | Other biliary tract disease | digestive | 3,892 | 391,307 | 13,762 |
| 362 | Other retinal disorders | sense organs | 3,867 | 396,859 | 8,235 |
| 728.7 | Fasciitis | musculoskeletal | 3,843 | 378,711 | 26,407 |
| 870 | Open wounds of head; neck; and trunk | injuries & poisonings | 3,837 | 400,426 | 4,698 |
| 427.9 | Palpitations | circulatory system | 3,832 | 380,919 | 24,210 |
| 288 | Diseases of white blood cells | hematopoietic | 3,788 | 401,375 | 3,798 |
| 733 | Other disorders of bone and cartilage | musculoskeletal | 3,773 | 391,041 | 14,147 |
| 274 | Gout and other crystal arthropathies | endocrine/metabolic | 3,763 | 405,198 | 0 |
| 720 | Spinal stenosis | musculoskeletal | 3,733 | 391,917 | 13,311 |
| 550.4 | Umbilical hernia | digestive | 3,727 | 361,617 | 43,617 |
| 741 | Symptoms and disorders of the joints | musculoskeletal | 3,634 | 402,633 | 2,694 |
| 451.2 | Phlebitis and thrombophlebitis of lower extremities | circulatory system | 3,587 | 369,592 | 35,782 |
| 418.1 | Precordial pain | circulatory system | 3,582 | 377,532 | 27,847 |
| 598 | Abnormal findings on examination of urine | genitourinary | 3,547 | 405,414 | 0 |
| 716.1 | Unspecified polyarthropathy or polyarthritis | musculoskeletal | 3,535 | 365,819 | 39,607 |
| 458.9 | Hypotension NOS | circulatory system | 3,518 | 387,905 | 17,538 |
| 728.71 | Contracture of palmar fascia [Dupuytren's disease] | musculoskeletal | 3,503 | 378,711 | 26,747 |
| 573.7 | Abnormal results of function study of liver | digestive | 3,479 | 400,055 | 5,427 |
| 709 | Diffuse diseases of connective tissue | dermatologic | 3,463 | 399,404 | 6,094 |
| 550.5 | Ventral hernia | digestive | 3,448 | 361,617 | 43,896 |
| 798 | Malaise and fatigue | symptoms | 3,429 | 405,532 | 0 |
| 540 | Appendiceal conditions | digestive | 3,409 | 405,552 | 0 |
| 537 | Other disorders of stomach and duodenum | digestive | 3,404 | 378,124 | 27,433 |
| 586 | Other disorders of the kidney and ureters | genitourinary | 3,362 | 397,602 | 7,997 |
| 804 | Fracture of hand or wrist | injuries & poisonings | 3,357 | 387,765 | 17,839 |
| 426.3 | Bundle branch block | circulatory system | 3,353 | 380,919 | 24,689 |
| 560.4 | Other intestinal obstruction | digestive | 3,346 | 334,783 | 70,832 |
| 597.1 | Urethral stricture (not specified as infectious) | genitourinary | 3,331 | 394,699 | 10,931 |
| 471 | Nasal polyps | respiratory | 3,311 | 390,045 | 15,605 |
| 568 | Other disorders of peritoneum | digestive | 3,308 | 387,338 | 18,315 |
| 368 | Visual disturbances | sense organs | 3,307 | 405,654 | 0 |
| 859 | Complication due to other implant and internal device | injuries & poisonings | 3,265 | 394,929 | 10,767 |
| 361 | Retinal detachments and defects | sense organs | 3,263 | 397,761 | 7,937 |
| 427.3 | Other specified cardiac dysrhythmias | circulatory system | 3,236 | 380,919 | 24,806 |
| 427.1 | Paroxysmal tachycardia, unspecified | circulatory system | 3,225 | 380,919 | 24,817 |
| 540.1 | Appendicitis | digestive | 3,217 | 405,552 | 192 |
| 274.1 | Gout | endocrine/metabolic | 3,195 | 405,198 | 568 |
| 555.2 | Ulcerative colitis | digestive | 3,195 | 334,783 | 70,983 |
| 579 | Other symptoms involving abdomen and pelvis | digestive | 3,195 | 385,157 | 20,609 |
| 594.1 | Calculus of kidney | genitourinary | 3,191 | 401,005 | 4,765 |
| 727.4 | Ganglion and cyst of synovium, tendon, and bursa | musculoskeletal | 3,185 | 378,711 | 27,065 |
| 288.1 | Decreased white blood cell count | hematopoietic | 3,184 | 401,375 | 4,402 |
| 288.11 | Neutropenia | hematopoietic | 3,184 | 401,375 | 4,402 |
| 371 | Inflammation of the eye | sense organs | 3,174 | 399,306 | 6,481 |
| 858 | Complication of internal orthopedic device | injuries & poisonings | 3,167 | 394,929 | 10,865 |
| 41.1 | Staphylococcus infections | infectious diseases | 3,149 | 393,897 | 11,915 |
| 990 | Effects radiation NOS | injuries & poisonings | 3,130 | 403,295 | 2,536 |
| 568.1 | Peritoneal adhesions (postoperative) (postinfection) | digestive | 3,108 | 387,338 | 18,515 |
| 702.2 | Seborrheic keratosis | dermatologic | 3,092 | 403,439 | 2,430 |
| 521 | Diseases of hard tissues of teeth | digestive | 3,091 | 398,136 | 7,734 |
| 592 | Cystitis and urethritis | genitourinary | 3,088 | 379,936 | 25,937 |
| 153.2 | Colon cancer | neoplasms | 3,051 | 382,756 | 23,154 |
| 521.1 | Dental caries | digestive | 3,051 | 398,136 | 7,774 |
| 531.3 | Duodenal ulcer | digestive | 3,002 | 401,525 | 4,434 |
| 293 | Symptoms involving head and neck | mental disorders | 2,986 | 405,975 | 0 |
| 394.2 | Mitral valve disease | circulatory system | 2,985 | 402,421 | 3,555 |
| 819 | Skull and face fracture and other intercranial injury | injuries & poisonings | 2,957 | 405,554 | 450 |
| 592.1 | Cystitis | genitourinary | 2,948 | 379,936 | 26,077 |
| 496.2 | Chronic bronchitis | respiratory | 2,934 | 375,505 | 30,522 |
| 433.3 | Cerebral ischemia | circulatory system | 2,920 | 399,017 | 7,024 |
| 870.3 | Other open wound of head and face | injuries & poisonings | 2,919 | 400,426 | 5,616 |
| 742 | Derangement of joint, nontraumatic | musculoskeletal | 2,913 | 402,633 | 3,415 |
| 687.4 | Disturbance of skin sensation | dermatologic | 2,900 | 403,407 | 2,654 |
| 571 | Chronic liver disease and cirrhosis | digestive | 2,895 | 400,055 | 6,011 |
| 395.1 | Nonrheumatic mitral valve disorders | circulatory system | 2,892 | 402,421 | 3,648 |
| 512.8 | Cough | respiratory | 2,884 | 399,833 | 6,244 |
| 340 | Migraine | neurological | 2,870 | 398,780 | 7,311 |
| 722.6 | Degeneration of intervertebral disc | musculoskeletal | 2,846 | 391,917 | 14,198 |
| 276.5 | Hypovolemia | endocrine/metabolic | 2,834 | 401,506 | 4,621 |
| 994 | Sepsis and SIRS | infectious diseases | 2,811 | 406,150 | 0 |
| 994.2 | Sepsis | infectious diseases | 2,811 | 406,150 | 0 |
| 79 | Viral infection | infectious diseases | 2,806 | 403,316 | 2,839 |
| 747 | Cardiac and circulatory congenital anomalies | congenital anomalies | 2,796 | 406,165 | 0 |
| 574.3 | Cholecystitis without cholelithiasis | digestive | 2,761 | 391,307 | 14,893 |
| 727.1 | Synovitis and tenosynovitis | musculoskeletal | 2,754 | 378,711 | 27,496 |
| 41.4 | E. coli | infectious diseases | 2,744 | 393,897 | 12,320 |
| 350 | Abnormal movement | neurological | 2,744 | 406,217 | 0 |
| 8.5 | Bacterial enteritis | infectious diseases | 2,737 | 399,970 | 6,254 |
| 709.7 | Unspecified diffuse connective tissue disease | dermatologic | 2,720 | 399,404 | 6,837 |
| 165 | Cancer within the respiratory system | neoplasms | 2,700 | 406,226 | 35 |
| 496.21 | Obstructive chronic bronchitis | respiratory | 2,698 | 375,505 | 30,758 |
| 172.1 | Melanomas of skin, dx or hx | neoplasms | 2,691 | 395,071 | 11,199 |
| 172.11 | Melanomas of skin | neoplasms | 2,691 | 395,071 | 11,199 |
| 577 | Diseases of pancreas | digestive | 2,690 | 406,271 | 0 |
| 525 | Other diseases of the teeth and supporting structures | digestive | 2,689 | 398,136 | 8,136 |
| 250.1 | Type 1 diabetes | endocrine/metabolic | 2,660 | 388,756 | 17,545 |
| 578.2 | Blood in stool | digestive | 2,639 | 385,157 | 21,165 |
| 198.4 | Secondary malignant neoplasm of liver | neoplasms | 2,638 | 370,604 | 35,719 |
| 574.2 | Calculus of bile duct | digestive | 2,634 | 391,307 | 15,020 |
| 473 | Diseases of the larynx and vocal cords | respiratory | 2,630 | 390,045 | 16,286 |
| 585.3 | Chronic renal failure [CKD] | genitourinary | 2,629 | 397,602 | 8,730 |
| 289.4 | Lymphadenitis | hematopoietic | 2,622 | 401,375 | 4,964 |
| 747.1 | Cardiac congenital anomalies | congenital anomalies | 2,618 | 406,165 | 178 |
| 540.11 | Acute appendicitis | digestive | 2,608 | 405,552 | 801 |
| 475 | Chronic sinusitis | respiratory | 2,602 | 390,045 | 16,314 |
| 702.1 | Actinic keratosis | dermatologic | 2,594 | 403,439 | 2,928 |
| 416 | Cardiomegaly | circulatory system | 2,573 | 402,375 | 4,013 |
| 443.9 | Peripheral vascular disease, unspecified | circulatory system | 2,566 | 400,595 | 5,800 |
| 509 | Respiratory failure, insufficiency, arrest | respiratory | 2,565 | 397,411 | 8,985 |
| 514 | Abnormal findings examination of lungs | respiratory | 2,500 | 406,461 | 0 |
| 426.9 | Cardiac pacemaker/device in situ | circulatory system | 2,487 | 380,919 | 25,555 |
| 81 | Infection/inflammation of internal prosthetic device; implant; and graft | infectious diseases | 2,485 | 402,343 | 4,133 |
| 477 | Epistaxis or throat hemorrhage | respiratory | 2,456 | 390,045 | 16,460 |
| 520 | Disorders of tooth development | digestive | 2,449 | 398,136 | 8,376 |
| 367 | Disorders of refraction and accommodation; blindness and low vision | sense organs | 2,431 | 406,530 | 0 |
| 189.2 | Cancer of bladder | neoplasms | 2,427 | 404,796 | 1,738 |
| 695 | Erythematous conditions | dermatologic | 2,420 | 402,672 | 3,869 |
| 594.3 | Calculus of ureter | genitourinary | 2,417 | 401,005 | 5,539 |
| 229 | Benign neoplasm of unspecified sites | neoplasms | 2,402 | 406,559 | 0 |
| 371.3 | Inflammation of eyelids | sense organs | 2,396 | 399,306 | 7,259 |
| 764 | Sciatica | symptoms | 2,383 | 405,481 | 1,097 |
| 520.2 | Disturbances in tooth eruption | digestive | 2,364 | 398,136 | 8,461 |
| 427.11 | Paroxysmal supraventricular tachycardia | circulatory system | 2,359 | 380,919 | 25,683 |
| 701.2 | Scar conditions and fibrosis of skin | dermatologic | 2,356 | 403,875 | 2,730 |
| 801 | Fracture of ankle and foot | injuries & poisonings | 2,339 | 387,765 | 18,857 |
| 465 | Acute upper respiratory infections of multiple or unspecified sites | respiratory | 2,335 | 406,447 | 179 |
| 686.1 | Carbuncle and furuncle | dermatologic | 2,302 | 397,635 | 9,024 |
| 696 | Psoriasis and related disorders | dermatologic | 2,293 | 398,199 | 8,469 |
| 426.91 | Cardiac pacemaker in situ | circulatory system | 2,283 | 380,919 | 25,759 |
| 202 | Cancer of other lymphoid, histiocytic tissue | neoplasms | 2,270 | 404,466 | 2,225 |
| 381 | Otitis media and Eustachian tube disorders | sense organs | 2,259 | 404,888 | 1,814 |
| 696.4 | Psoriasis | dermatologic | 2,237 | 398,199 | 8,525 |
| 345.3 | Convulsions | neurological | 2,232 | 395,209 | 11,520 |
| 375 | Disorders of lacrimal system | sense organs | 2,218 | 401,245 | 5,498 |
| 198.2 | Secondary malignancy of respiratory organs | neoplasms | 2,211 | 370,604 | 36,146 |
| 427.7 | Tachycardia NOS | circulatory system | 2,193 | 380,919 | 25,849 |
| 362.2 | Degeneration of macula and posterior pole of retina | sense organs | 2,191 | 396,859 | 9,911 |
| 292.4 | Altered mental status | mental disorders | 2,189 | 402,383 | 4,389 |
| 362.29 | Macular degeneration (senile) of retina NOS | sense organs | 2,188 | 396,859 | 9,914 |
| 516 | Abnormal sputum | respiratory | 2,167 | 406,794 | 0 |
| 800.3 | Fracture of tibia and fibula | injuries & poisonings | 2,162 | 387,765 | 19,034 |
| 687.1 | Rash and other nonspecific skin eruption | dermatologic | 2,157 | 403,407 | 3,397 |
| 198.6 | Secondary malignancy of bone | neoplasms | 2,151 | 370,604 | 36,206 |
| 189.21 | Malignant neoplasm of bladder | neoplasms | 2,146 | 404,796 | 2,019 |
| 433.31 | Transient cerebral ischemia | circulatory system | 2,146 | 399,017 | 7,798 |
| 112 | Candidiasis | infectious diseases | 2,134 | 406,301 | 526 |
| 275 | Disorders of mineral metabolism | endocrine/metabolic | 2,127 | 406,834 | 0 |
| 426.2 | Atrioventricular [AV] block | circulatory system | 2,125 | 380,919 | 25,917 |
| 939 | Atopic/contact dermatitis due to other or unspecified | dermatologic | 2,110 | 404,817 | 2,034 |
| 557 | Intestinal malabsorption (non-celiac) | digestive | 2,103 | 334,783 | 72,075 |
| 165.1 | Cancer of bronchus; lung | neoplasms | 2,101 | 406,226 | 634 |
| 530.9 | Heartburn | digestive | 2,100 | 369,275 | 37,586 |
| 153.3 | Malignant neoplasm of rectum, rectosigmoid junction, and anus | neoplasms | 2,095 | 382,756 | 24,110 |
| 379.3 | Aphakia and other disorders of lens | sense organs | 1,825 | 401,245 | 5,891 |
| 381.1 | Otitis media | sense organs | 1,824 | 404,888 | 2,249 |
| 782 | Symptoms involving skin and other integumentary tissue | symptoms | 1,816 | 407,145 | 0 |
| 800.1 | Fracture of neck of femur | injuries & poisonings | 1,814 | 387,765 | 19,382 |
| 442 | Other aneurysm | circulatory system | 1,808 | 400,595 | 6,558 |
| 747.13 | Congenital anomalies of great vessels | congenital anomalies | 1,799 | 406,165 | 997 |
| 414 | Other forms of chronic heart disease | circulatory system | 1,796 | 377,103 | 30,062 |
| 430 | Intracranial hemorrhage | circulatory system | 1,796 | 399,017 | 8,148 |
| 761 | Cervicalgia | symptoms | 1,796 | 407,165 | 0 |
| 522 | Diseases of pulp and periapical tissues | digestive | 1,795 | 398,136 | 9,030 |
| 202.2 | Non-Hodgkins lymphoma | neoplasms | 1,793 | 404,466 | 2,702 |
| 287 | Purpura and other hemorrhagic conditions | hematopoietic | 1,791 | 406,281 | 889 |
| 334 | Degenerative disease of the spinal cord | neurological | 1,789 | 395,209 | 11,963 |
| 854 | Complications of cardiac/vascular device, implant, and graft | injuries & poisonings | 1,789 | 394,929 | 12,243 |
| 426.32 | Left bundle branch block | circulatory system | 1,757 | 380,919 | 26,285 |
| 509.2 | Respiratory insufficiency | respiratory | 1,749 | 397,411 | 9,801 |
| 555.1 | Regional enteritis | digestive | 1,743 | 334,783 | 72,435 |
| 523 | Gingival and periodontal diseases | digestive | 1,742 | 398,136 | 9,083 |
| 496.1 | Emphysema | respiratory | 1,727 | 375,505 | 31,729 |
| 401.2 | Hypertensive heart and/or renal disease | circulatory system | 1,719 | 330,366 | 76,876 |
| 803.1 | Fracture of humerus | injuries & poisonings | 1,692 | 387,765 | 19,504 |
| 561.2 | Flatulence | digestive | 1,689 | 334,783 | 72,489 |
| 696.41 | Psoriasis vulgaris | dermatologic | 1,684 | 398,199 | 9,078 |
| 823 | Torus fracture | injuries & poisonings | 1,674 | 385,157 | 22,130 |
| 530.2 | Esophageal bleeding (varices/hemorrhage) | digestive | 1,672 | 369,275 | 38,014 |
| 715 | Other inflammatory spondylopathies | musculoskeletal | 1,671 | 365,085 | 42,205 |
| 571.5 | Other chronic nonalcoholic liver disease | digestive | 1,664 | 400,055 | 7,242 |
| 596.5 | Functional disorders of bladder | genitourinary | 1,664 | 394,699 | 12,598 |
| 204 | Leukemia | neoplasms | 1,661 | 404,466 | 2,834 |
| 559 | Ileostomy status | digestive | 1,660 | 334,783 | 72,518 |
| 579.8 | Nonspecific abnormal findings in stool contents | digestive | 1,659 | 385,157 | 22,145 |
| 564.8 | Abnormal findings on exam of gastrointestinal tract/ abdominal area | digestive | 1,650 | 334,783 | 72,528 |
| 736 | Other acquired deformities of limbs | musculoskeletal | 1,649 | 394,914 | 12,398 |
| 782.3 | Edema | symptoms | 1,648 | 407,145 | 168 |
| 41.2 | Streptococcus infection | infectious diseases | 1,644 | 393,897 | 13,420 |
| 724.9 | Other unspecified back disorders | musculoskeletal | 1,617 | 391,917 | 15,427 |
| 228 | Hemangioma and lymphangioma, any site | neoplasms | 1,603 | 407,358 | 0 |
| 350.2 | Abnormality of gait | neurological | 1,601 | 406,217 | 1,143 |
| 751 | Genitourinary congenital anomalies | congenital anomalies | 1,596 | 406,730 | 635 |
| 611 | Abnormal findings on mammogram or breast exam | genitourinary | 1,580 | 401,746 | 5,635 |
| 287.3 | Thrombocytopenia | hematopoietic | 1,563 | 406,281 | 1,117 |
| 735.23 | Hallux rigidus | musculoskeletal | 1,561 | 394,914 | 12,486 |
| 401.22 | Hypertensive chronic kidney disease | circulatory system | 1,548 | 330,366 | 77,047 |
| 572 | Ascites (non malignant) | digestive | 1,547 | 400,055 | 7,359 |
| 611.3 | Lump or mass in breast | genitourinary | 1,525 | 401,746 | 5,690 |
| 580 | Nephritis; nephrosis; renal sclerosis | genitourinary | 1,522 | 397,602 | 9,837 |
| 738.4 | Acquired spondylolisthesis | musculoskeletal | 1,521 | 394,914 | 12,526 |
| 803.3 | Fracture of clavicle or scapula | injuries & poisonings | 1,521 | 387,765 | 19,675 |
| 198.3 | Secondary malignant neoplasm of digestive systems | neoplasms | 1,519 | 370,604 | 36,838 |
| 292.1 | Aphasia/speech disturbance | mental disorders | 1,514 | 402,383 | 5,064 |
| 574.11 | Cholelithiasis with acute cholecystitis | digestive | 1,513 | 391,307 | 16,141 |
| 433.21 | Cerebral artery occlusion, with cerebral infarction | circulatory system | 1,501 | 399,017 | 8,443 |
| 342 | Hemiplegia | neurological | 1,500 | 395,209 | 12,252 |
| 395.6 | Heart valve replaced | circulatory system | 1,499 | 402,421 | 5,041 |
| 426.31 | Right bundle branch block | circulatory system | 1,498 | 380,919 | 26,544 |
| 402 | Elevated blood pressure reading without diagnosis of hypertension | circulatory system | 1,487 | 330,366 | 77,108 |
| 916 | Contusion | injuries & poisonings | 1,486 | 407,475 | 0 |
| 380 | Disorders of external ear | sense organs | 1,451 | 407,510 | 0 |
| 378 | Strabismus and other disorders of binocular eye movements | sense organs | 1,442 | 401,245 | 6,274 |
| 276.14 | Hypopotassemia | endocrine/metabolic | 1,430 | 401,506 | 6,025 |
| 348 | Other conditions of brain | neurological | 1,426 | 395,209 | 12,326 |
| 741.4 | Joint effusions | musculoskeletal | 1,425 | 402,633 | 4,903 |
| 277 | Other disorders of metabolism | endocrine/metabolic | 1,424 | 407,537 | 0 |
| 199 | Neoplasm of uncertain behavior | neoplasms | 1,412 | 370,604 | 36,945 |
| 575.7 | Other disorders of gallbladder | digestive | 1,412 | 391,307 | 16,242 |
| 585.2 | Renal failure NOS | genitourinary | 1,412 | 397,602 | 9,947 |
| 352 | Disorders of other cranial nerves | neurological | 1,393 | 394,067 | 13,501 |
| 361.1 | Retinal detachment with retinal defect | sense organs | 1,392 | 397,761 | 9,808 |
| 442.1 | Aortic aneurysm | circulatory system | 1,374 | 400,595 | 6,992 |
| 379.2 | Disorders of vitreous body | sense organs | 1,372 | 401,245 | 6,344 |
| 357 | Inflammatory and toxic neuropathy | neurological | 1,368 | 406,852 | 741 |
| 384 | Other disorders of tympanic membrane | sense organs | 1,364 | 404,888 | 2,709 |
| 335 | Multiple sclerosis | neurological | 1,356 | 395,209 | 12,396 |
| 805 | Fracture of vertebral column without mention of spinal cord injury | injuries & poisonings | 1,352 | 387,765 | 19,844 |
| 458.1 | Orthostatic hypotension | circulatory system | 1,347 | 387,905 | 19,709 |
| 250.7 | Diabetic retinopathy | endocrine/metabolic | 1,339 | 396,859 | 10,763 |
| 447 | Other disorders of arteries and arterioles | circulatory system | 1,333 | 400,595 | 7,033 |
| 440 | Atherosclerosis | circulatory system | 1,324 | 400,595 | 7,042 |
| 590 | Pyelonephritis | genitourinary | 1,324 | 379,936 | 27,701 |
| 907 | Injuries to the nervous system | injuries & poisonings | 1,315 | 407,496 | 150 |
| 809 | Fracture of unspecified bones | injuries & poisonings | 1,304 | 387,765 | 19,892 |
| 250.23 | Type 2 diabetes with ophthalmic manifestations | endocrine/metabolic | 1,298 | 388,756 | 18,907 |
| 703 | Diseases of nail, NOS | dermatologic | 1,287 | 402,357 | 5,317 |
| 420.2 | Pericarditis | circulatory system | 1,273 | 405,779 | 1,909 |
| 733.8 | Malunion and nonunion of fracture | musculoskeletal | 1,268 | 391,041 | 16,652 |
| 433.8 | Late effects of cerebrovascular disease | circulatory system | 1,263 | 399,017 | 8,681 |
| 394.3 | Aortic valve disease | circulatory system | 1,260 | 402,421 | 5,280 |
| 586.2 | Cyst of kidney, acquired | genitourinary | 1,260 | 397,602 | 10,099 |
| 367.1 | Myopia | sense organs | 1,257 | 406,530 | 1,174 |
| 961 | Poisoning by other antiinfectives | injuries & poisonings | 1,256 | 381,797 | 25,908 |
| 425 | Cardiomyopathy | circulatory system | 1,247 | 405,779 | 1,935 |
| 857 | Mechanical complication of unspecified genitourinary device, implant, and graft | injuries & poisonings | 1,241 | 394,929 | 12,791 |
| 529 | Diseases and other conditions of the tongue | digestive | 1,236 | 403,323 | 4,402 |
| 1005 | Other symptoms | symptoms | 1,222 | 407,739 | 0 |
| 523.3 | Periodontitis (acute or chronic) | digestive | 1,222 | 398,136 | 9,603 |
| 70 | Viral hepatitis | infectious diseases | 1,215 | 403,316 | 4,430 |
| 1001 | Foreign body injury | injuries & poisonings | 1,210 | 407,751 | 0 |
| 261 | Vitamin deficiency | endocrine/metabolic | 1,208 | 406,492 | 1,261 |
| 425.1 | Primary/intrinsic cardiomyopathies | circulatory system | 1,208 | 405,779 | 1,974 |
| 275.5 | Disorders of calcium/phosphorus metabolism | endocrine/metabolic | 1,204 | 406,834 | 923 |
| 599.3 | Dysuria | genitourinary | 1,199 | 384,930 | 22,832 |
| 433.1 | Occlusion and stenosis of precerebral arteries | circulatory system | 1,185 | 399,017 | 8,759 |
| 535.1 | Acute gastritis | digestive | 1,184 | 378,124 | 29,653 |
| 766 | Neuralgia, neuritis, and radiculitis NOS | symptoms | 1,181 | 405,481 | 2,299 |
| 522.5 | Periapical abscess | digestive | 1,177 | 398,136 | 9,648 |
| 506 | Empyema and pneumothorax | respiratory | 1,174 | 397,411 | 10,376 |
| 411.9 | Other acute and subacute forms of ischemic heart disease | circulatory system | 1,169 | 377,103 | 30,689 |
| 276.4 | Acid-base balance disorder | endocrine/metabolic | 1,161 | 401,506 | 6,294 |
| 717 | Polymyalgia Rheumatica | musculoskeletal | 1,152 | 407,809 | 0 |
| 965.1 | Opiates and related narcotics causing adverse effects in therapeutic use | injuries & poisonings | 1,150 | 381,797 | 26,014 |
| 443.1 | Raynaud's syndrome | circulatory system | 1,148 | 400,595 | 7,218 |
| 241 | Nontoxic nodular goiter | endocrine/metabolic | 1,143 | 391,429 | 16,389 |
| 353 | Nerve root and plexus disorders | neurological | 1,139 | 394,067 | 13,755 |
| 427.4 | Cardiac arrest and ventricular fibrillation | circulatory system | 1,137 | 380,919 | 26,905 |
| 737 | Curvature of spine | musculoskeletal | 1,134 | 394,914 | 12,913 |
| 281 | Other deficiency anemia | hematopoietic | 1,133 | 390,026 | 17,802 |
| 332 | Parkinson's disease | neurological | 1,127 | 395,209 | 12,625 |
| 474.2 | Chronic tonsillitis and adenoiditis | respiratory | 1,126 | 390,045 | 17,790 |
| 244.1 | Secondary hypothyroidism | endocrine/metabolic | 1,117 | 391,429 | 16,415 |
| 331 | Other cerebral degenerations | neurological | 1,112 | 395,209 | 12,640 |
| 215 | Other benign neoplasm of connective and other soft tissue | neoplasms | 1,110 | 401,613 | 6,238 |
| 473.4 | Voice disturbance | respiratory | 1,086 | 390,045 | 17,830 |
| 281.1 | Megaloblastic anemia | hematopoietic | 1,076 | 390,026 | 17,859 |
| 374.1 | Ectropion or entropion | sense organs | 1,068 | 399,306 | 8,587 |
| 296.1 | Bipolar | mental disorders | 1,064 | 365,476 | 42,421 |
| 556 | Ulceration of the lower GI tract | digestive | 1,063 | 334,783 | 73,115 |
| 737.3 | Kyphoscoliosis and scoliosis | musculoskeletal | 1,063 | 394,914 | 12,984 |
| 476 | Allergic rhinitis | respiratory | 1,060 | 390,045 | 17,856 |
| 394.7 | Disease of tricuspid valve | circulatory system | 1,058 | 402,421 | 5,482 |
| 260 | Protein-calorie malnutrition | endocrine/metabolic | 1,057 | 406,492 | 1,412 |
| 158 | Neoplasm of unspecified nature of digestive system | neoplasms | 1,056 | 393,372 | 14,533 |
| 276.41 | Acidosis | endocrine/metabolic | 1,055 | 401,506 | 6,400 |
| 396 | Abnormal heart sounds | circulatory system | 1,049 | 402,421 | 5,491 |
| 189.1 | Cancer of kidney and renal pelvis | neoplasms | 1,045 | 404,796 | 3,120 |
| 384.4 | Perforation of tympanic membrane | sense organs | 1,044 | 404,888 | 3,029 |
| 365.1 | Open-angle glaucoma | sense organs | 1,043 | 397,761 | 10,157 |
| 575.8 | Other disorders of biliary tract | digestive | 1,040 | 391,307 | 16,614 |
| 365.11 | Primary open angle glaucoma | sense organs | 1,037 | 397,761 | 10,163 |
| 580.1 | Glomerulonephritis | genitourinary | 1,033 | 397,602 | 10,326 |
| 729.1 | Rheumatism, unspecified and fibrositis | musculoskeletal | 1,010 | 378,711 | 29,240 |
| 78 | Viral warts & HPV | infectious diseases | 1,007 | 403,316 | 4,638 |
| 1008 | Crushing or internal injury to organs | injuries & poisonings | 1,007 | 407,954 | 0 |
| 189.11 | Malignant neoplasm of kidney, except pelvis | neoplasms | 1,002 | 404,796 | 3,163 |
| 200 | Myeloproliferative disease | neoplasms | 995 | 404,466 | 3,500 |
| 426.21 | First degree AV block | circulatory system | 988 | 380,919 | 27,054 |
| 694 | Dyschromia and Vitiligo | dermatologic | 987 | 402,672 | 5,302 |
| 210 | Benign neoplasm of lip, oral cavity, and pharynx | neoplasms | 984 | 406,821 | 1,156 |
| 276.13 | Hyperpotassemia | endocrine/metabolic | 975 | 401,506 | 6,480 |
| 703.1 | Ingrowing nail | dermatologic | 970 | 402,357 | 5,634 |
| 472 | Chronic pharyngitis and nasopharyngitis | respiratory | 966 | 390,045 | 17,950 |
| 526 | Diseases of the jaws | digestive | 960 | 398,136 | 9,865 |
| 290.1 | Dementias | mental disorders | 956 | 402,383 | 5,622 |
| 251 | Other disorders of pancreatic internal secretion | endocrine/metabolic | 943 | 405,386 | 2,632 |
| 571.8 | Liver abscess and sequelae of chronic liver disease | digestive | 942 | 400,055 | 7,964 |
| 612 | Breast conditions, congenital or relating to hormones | genitourinary | 942 | 401,746 | 6,273 |
| 286 | Coagulation defects | hematopoietic | 941 | 406,281 | 1,739 |
| 251.1 | Hypoglycemia | endocrine/metabolic | 939 | 386,319 | 21,703 |
| 427.12 | Paroxysmal ventricular tachycardia | circulatory system | 938 | 380,919 | 27,104 |
| 427.42 | Cardiac arrest | circulatory system | 927 | 380,919 | 27,115 |
| 427.5 | Arrhythmia (cardiac) NOS | circulatory system | 922 | 380,919 | 27,120 |
| 444 | Arterial embolism and thrombosis | circulatory system | 921 | 400,595 | 7,445 |
| 378.1 | Strabismus (not specified as paralytic) | sense organs | 918 | 401,245 | 6,798 |
| 586.4 | Stricture/obstruction of ureter | genitourinary | 913 | 397,602 | 10,446 |
| 707.1 | Decubitus ulcer | dermatologic | 913 | 406,973 | 1,075 |
| 512.9 | Other dyspnea | respiratory | 912 | 399,833 | 8,216 |
| 293.1 | Swelling, mass, or lump in head and neck [Space occupying lesion, intracranial NOS] | mental disorders | 907 | 405,975 | 2,079 |
| 442.11 | Abdominal aortic aneurysm | circulatory system | 903 | 400,595 | 7,463 |
| 345.1 | Epilepsy | neurological | 901 | 395,209 | 12,851 |
| 375.2 | Epiphora | sense organs | 899 | 401,245 | 6,817 |
| 573.5 | Jaundice (not of newborn) | digestive | 897 | 400,055 | 8,009 |
| 592.12 | Chronic cystitis | genitourinary | 892 | 379,936 | 28,133 |
| 333 | Extrapyramidal disease and abnormal movement disorders | neurological | 891 | 395,209 | 12,861 |
| 350.1 | Abnormal involuntary movements | neurological | 888 | 406,217 | 1,856 |
| 502 | Postinflammatory pulmonary fibrosis | respiratory | 887 | 397,411 | 10,663 |
| 567 | Peritonitis and retroperitoneal infections | digestive | 887 | 387,338 | 20,736 |
| 352.2 | Facial nerve disorders [CN7] | neurological | 880 | 394,067 | 14,014 |
| 252 | Disorders of parathyroid gland | endocrine/metabolic | 877 | 405,386 | 2,698 |
| 227 | Benign neoplasm of other endocrine glands and related structures | neoplasms | 876 | 407,399 | 686 |
| 961.1 | Poisoning/allergy of sulfonamides | injuries & poisonings | 874 | 381,797 | 26,290 |
| 447.1 | Stricture of artery | circulatory system | 873 | 400,595 | 7,493 |
| 465.2 | Acute pharyngitis | respiratory | 870 | 406,447 | 1,644 |
| 8.6 | Viral Enteritis | infectious diseases | 862 | 399,970 | 8,129 |
| 726.3 | Bursitis | musculoskeletal | 858 | 378,711 | 29,392 |
| 381.11 | Suppurative and unspecified otitis media | sense organs | 856 | 404,888 | 3,217 |
| 751.2 | Congenital anomalies of urinary system | congenital anomalies | 855 | 406,730 | 1,376 |
| 612.2 | Hypertrophy of breast (Gynecomastia) | genitourinary | 854 | 401,746 | 6,361 |
| 295 | Schizophrenia and other psychotic disorders | mental disorders | 850 | 365,476 | 42,635 |
| 362.4 | Retinal vascular changes and abnomalities | sense organs | 849 | 396,859 | 11,253 |
| 580.14 | Chronic glomerulonephritis, NOS | genitourinary | 845 | 397,602 | 10,514 |
| 260.6 | Anorexia | endocrine/metabolic | 835 | 406,492 | 1,634 |
| 38.1 | Gram negative septicemia | infectious diseases | 832 | 393,897 | 14,232 |
| 560.3 | Peritoneal or intestinal adhesions | digestive | 832 | 334,783 | 73,346 |
| 225 | Benign neoplasm of brain and other parts of nervous system | neoplasms | 828 | 407,239 | 894 |
| 385 | Other disorders of middle ear and mastoid | sense organs | 828 | 404,888 | 3,245 |
| 446 | Polyarteritis nodosa and allied conditions | circulatory system | 828 | 400,595 | 7,538 |
| 743.9 | Osteopenia or other disorder of bone and cartilage | musculoskeletal | 820 | 401,279 | 6,862 |
| 705 | Disorders of sweat glands | dermatologic | 817 | 399,255 | 8,889 |
| 430.1 | Subarachnoid hemorrhage | circulatory system | 812 | 399,017 | 9,132 |
| 440.2 | Atherosclerosis of the extremities | circulatory system | 811 | 400,595 | 7,555 |
| 198.5 | Secondary malignancy of brain/spine | neoplasms | 806 | 370,604 | 37,551 |
| 317.11 | Alcoholic liver damage | mental disorders | 802 | 379,355 | 28,804 |
| 429 | Ill-defined descriptions and complications of heart disease | circulatory system | 796 | 402,834 | 5,331 |
| 694.2 | Other dyschromia | dermatologic | 783 | 402,672 | 5,506 |
| 698 | Pruritus and related conditions | dermatologic | 783 | 408,178 | 0 |
| 252.1 | Hyperparathyroidism | endocrine/metabolic | 781 | 405,386 | 2,794 |
| 510 | Other diseases of lung | respiratory | 781 | 408,180 | 0 |
| 695.7 | Prurigo and Lichen | dermatologic | 779 | 402,672 | 5,510 |
| 594.2 | Calculus of lower urinary tract | genitourinary | 778 | 401,005 | 7,178 |
| 575.2 | Obstruction of bile duct | digestive | 777 | 391,307 | 16,877 |
| 807 | Fracture of ribs | injuries & poisonings | 775 | 387,765 | 20,421 |
| 225.1 | Benign neoplasm of brain, cranial nerves, meninges | neoplasms | 774 | 407,239 | 948 |
| 338 | Pain | symptoms | 771 | 408,190 | 0 |
| 800.4 | Fracture of patella | injuries & poisonings | 768 | 387,765 | 20,428 |
| 386.3 | Labyrinthitis | sense organs | 767 | 402,827 | 5,367 |
| 372 | Disorders of conjunctiva | sense organs | 765 | 399,306 | 8,890 |
| 261.2 | Vitamin B-complex deficiencies | endocrine/metabolic | 754 | 406,492 | 1,715 |
| 281.11 | Pernicious anemia | hematopoietic | 754 | 390,026 | 18,181 |
| 364 | Corneal opacity and other disorders of cornea | sense organs | 732 | 397,761 | 10,468 |
| 368.2 | Diplopia and disorders of binocular vision | sense organs | 732 | 405,654 | 2,575 |
| 751.1 | Congenital anomalies of genital organs | congenital anomalies | 732 | 406,730 | 1,499 |
| 619.1 | Noninflammatory disorders of ovary, fallopian tube, and broad ligament | genitourinary | 730 | 399,629 | 8,602 |
| 367.9 | Blindness and low vision | sense organs | 723 | 406,530 | 1,708 |
| 613.1 | Inflammatory disease of breast | genitourinary | 723 | 406,978 | 1,260 |
| 802 | Fracture of pelvis | injuries & poisonings | 722 | 387,765 | 20,474 |
| 150 | Cancer of esophagus | neoplasms | 720 | 393,372 | 14,869 |
| 527 | Diseases of the salivary glands | digestive | 710 | 403,323 | 4,928 |
| 300.12 | Agorophobia, social phobia, and panic disorder | mental disorders | 709 | 365,476 | 42,776 |
| 696.42 | Psoriatic arthropathy | dermatologic | 708 | 398,199 | 10,054 |
| 365.2 | Primary angle-closure glaucoma | sense organs | 705 | 397,761 | 10,495 |
| 450 | Noninfectious disorders of lymphatic channels | circulatory system | 705 | 408,256 | 0 |
| 613.7 | Other signs and symptoms in breast | genitourinary | 704 | 406,978 | 1,279 |
| 750 | Digestive congenital anomalies | congenital anomalies | 703 | 406,730 | 1,528 |
| 285.2 | Anemia of chronic disease | hematopoietic | 702 | 390,026 | 18,233 |
| 292.3 | Memory loss | mental disorders | 700 | 402,383 | 5,878 |
| 430.2 | Intracerebral hemorrhage | circulatory system | 700 | 399,017 | 9,244 |
| 411.41 | Aneurysm and dissection of heart | circulatory system | 698 | 377,103 | 31,160 |
| 772 | Symptoms of the muscles | symptoms | 698 | 408,263 | 0 |
| 793 | Nonspecific abnormal findings on radiological and other examination of musculoskeletal system | injuries & poisonings | 698 | 408,263 | 0 |
| 979 | Adverse drug events and drug allergies | injuries & poisonings | 695 | 381,797 | 26,469 |
| 253 | Disorders of the pituitary gland and its hypothalamic control | endocrine/metabolic | 693 | 405,386 | 2,882 |
| 420.3 | Endocarditis | circulatory system | 686 | 405,779 | 2,496 |
| 250.4 | Abnormal glucose | endocrine/metabolic | 685 | 388,756 | 19,520 |
| 528.5 | Diseases of lips | digestive | 684 | 403,323 | 4,954 |
| 241.2 | Nontoxic multinodular goiter | endocrine/metabolic | 680 | 391,429 | 16,852 |
| 556.1 | Ulceration of intestine | digestive | 674 | 334,783 | 73,504 |
| 275.1 | Disorders of iron metabolism | hematopoietic | 669 | 406,834 | 1,458 |
| 523.31 | Acute periodontitis | digestive | 668 | 398,136 | 10,157 |
| 172.3 | Carcinoma in situ of skin | neoplasms | 667 | 395,071 | 13,223 |
| 344 | Other paralytic syndromes | neurological | 660 | 395,209 | 13,092 |
| 191 | Manlignant and unknown neoplasms of brain and nervous system | neoplasms | 655 | 407,239 | 1,067 |
| 290.2 | Delirium due to conditions classified elsewhere | mental disorders | 654 | 402,383 | 5,924 |
| 550.3 | Femoral hernia | digestive | 651 | 361,617 | 46,693 |
| 8.52 | Intestinal infection due to C. difficile | infectious diseases | 650 | 399,970 | 8,341 |
| 217 | Vascular hamartomas and non-neoplastic nevi | neoplasms | 649 | 400,618 | 7,694 |
| 145 | Cancer of mouth | neoplasms | 643 | 406,821 | 1,497 |
| 255 | Disorders of adrenal glands | endocrine/metabolic | 642 | 405,386 | 2,933 |
| 770 | Myalgia and myositis unspecified | symptoms | 642 | 408,319 | 0 |
| 291 | Other specified nonpsychotic and/or transient mental disorders | mental disorders | 641 | 402,383 | 5,937 |
| 530.5 | Disorders of esophageal motility | digestive | 639 | 369,275 | 39,047 |
| 454.11 | Varicose veins of lower extremity, symptomtic | circulatory system | 633 | 369,592 | 38,736 |
| 497 | Bronchitis | respiratory | 631 | 375,505 | 32,825 |
| 149 | Cancer of larynx, pharynx, nasal cavities | neoplasms | 628 | 406,821 | 1,512 |
| 715.2 | Ankylosing spondylitis | musculoskeletal | 620 | 365,085 | 43,256 |
| 531.1 | Hemorrhage from gastrointestinal ulcer | digestive | 617 | 401,525 | 6,819 |
| 274.2 | Crystal arthropathies | endocrine/metabolic | 616 | 405,198 | 3,147 |
| 368.9 | Subjective visual disturbances | sense organs | 615 | 405,654 | 2,692 |
| 741.2 | Stiffness of joint | musculoskeletal | 615 | 402,633 | 5,713 |
| 474.1 | Acute tonsillitis | respiratory | 614 | 390,045 | 18,302 |
| 710 | Osteomyelitis, periostitis, and other infections involving bone | musculoskeletal | 612 | 365,819 | 42,530 |
| 513 | Respiratory abnormalities | respiratory | 611 | 408,350 | 0 |
| 385.3 | Cholesteatoma | sense organs | 608 | 404,888 | 3,465 |
| 240 | Simple and unspecified goiter | endocrine/metabolic | 602 | 391,429 | 16,930 |
| 681.7 | Cellulitis and abscess of trunk | dermatologic | 602 | 397,635 | 10,724 |
| 740.2 | Osteoarthrosis, generalized | musculoskeletal | 599 | 380,522 | 27,840 |
| 217.1 | Nevus, non-neoplastic | neoplasms | 597 | 400,618 | 7,746 |
| 756 | Other congenital musculoskeletal anomalies | congenital anomalies | 594 | 407,831 | 536 |
| 727.5 | Rupture of synovium | musculoskeletal | 593 | 378,711 | 29,657 |
| 798.1 | Chronic fatigue syndrome | symptoms | 593 | 405,532 | 2,836 |
| 705.8 | Hyperhidrosis | dermatologic | 592 | 399,255 | 9,114 |
| 415.2 | Chronic pulmonary heart disease | circulatory system | 590 | 402,375 | 5,996 |
| 157 | Pancreatic cancer | neoplasms | 589 | 393,372 | 15,000 |
| 967 | Adverse effects of sedatives or other central nervous system depressants and anesthetics | injuries & poisonings | 588 | 381,797 | 26,576 |
| 426.24 | Atrioventricular block, complete | circulatory system | 587 | 380,919 | 27,455 |
| 686.3 | Pilonidal cyst | dermatologic | 587 | 397,635 | 10,739 |
| 501 | Pneumonitis due to inhalation of food or vomitus | respiratory | 586 | 397,411 | 10,964 |
| 747.11 | Cardiac shunt/ heart septal defect | congenital anomalies | 586 | 406,165 | 2,210 |
| 1000 | Burns | injuries & poisonings | 579 | 408,382 | 0 |
| 204.1 | Lymphoid leukemia | neoplasms | 578 | 404,466 | 3,917 |
| 386.1 | Meniere's disease | sense organs | 578 | 402,827 | 5,556 |
| 441 | Vascular insufficiency of intestine | circulatory system | 576 | 400,595 | 7,790 |
| 250.24 | Type 2 diabetes with neurological manifestations | endocrine/metabolic | 575 | 388,756 | 19,630 |
| 202.24 | Large cell lymphoma | neoplasms | 573 | 404,466 | 3,922 |
| 295.1 | Schizophrenia | mental disorders | 571 | 365,476 | 42,914 |
| 793.2 | Nonspecific abnormal findings on radiological and other examination of other intrathoracic organs (echocardiogram, etc) | circulatory system | 567 | 408,263 | 131 |
| 681.1 | Cellulitis and abscess of fingers/toes | dermatologic | 566 | 397,635 | 10,760 |
| 710.1 | Osteomyelitis | musculoskeletal | 565 | 365,819 | 42,577 |
| 523.32 | Chronic periodontitis | digestive | 561 | 398,136 | 10,264 |
| 274.21 | Chondrocalcinosis | endocrine/metabolic | 560 | 405,198 | 3,203 |
| 369 | Infection of the eye | sense organs | 560 | 399,306 | 9,095 |
| 452 | Other venous embolism and thrombosis | circulatory system | 558 | 369,592 | 38,811 |
| 444.1 | Arterial embolism and thrombosis of lower extremity artery | circulatory system | 557 | 400,595 | 7,809 |
| 151 | Cancer of stomach | neoplasms | 554 | 393,372 | 15,035 |
| 204.4 | Multiple myeloma | neoplasms | 552 | 404,466 | 3,943 |
| 791 | Gangrene | symptoms | 550 | 408,411 | 0 |
| 697 | Sarcoidosis | dermatologic | 548 | 402,672 | 5,741 |
| 946 | Anaphylactic shock NOS | injuries & poisonings | 548 | 404,817 | 3,596 |
| 733.4 | Aseptic necrosis of bone | musculoskeletal | 542 | 391,041 | 17,378 |
| 555.21 | Ulcerative colitis (chronic) | digestive | 539 | 334,783 | 73,639 |
| 368.1 | Amblyopia | sense organs | 538 | 405,654 | 2,769 |
| 427.6 | Premature beats | circulatory system | 536 | 380,919 | 27,506 |
| 743.13 | Other specified osteoporosis | musculoskeletal | 532 | 401,279 | 7,150 |
| 191.1 | Cancer of brain and nervous system | neoplasms | 531 | 407,239 | 1,191 |
| 303 | Psychogenic and somatoform disorders | mental disorders | 529 | 365,476 | 42,956 |
| 571.81 | Portal hypertension | digestive | 529 | 400,055 | 8,377 |
| 836 | Traumatic arthropathy | injuries & poisonings | 528 | 391,457 | 16,976 |
| 380.1 | Otitis externa | sense organs | 524 | 407,510 | 927 |
| 429.2 | Abnormal function study of cardiovascular system | circulatory system | 524 | 402,834 | 5,603 |
| 681.2 | Cellulitis and abscess of face/neck | dermatologic | 524 | 397,635 | 10,802 |
| 560.1 | Paralytic ileus | digestive | 522 | 334,783 | 73,656 |
| 289.5 | Diseases of spleen | hematopoietic | 515 | 401,375 | 7,071 |
| 389.4 | Tinnitus | sense organs | 515 | 404,562 | 3,884 |
| 577.2 | Chronic pancreatitis | digestive | 514 | 406,271 | 2,176 |
| 743.2 | Pathologic fracture | musculoskeletal | 514 | 401,279 | 7,168 |
| 709.2 | Sicca syndrome | dermatologic | 513 | 399,404 | 9,044 |
| 722.1 | Displacement of intervertebral disc | musculoskeletal | 513 | 391,917 | 16,531 |
| 966 | Poisoning by anticonvulsants and anti Parkinsonism drugs | injuries & poisonings | 509 | 381,797 | 26,655 |
| 818 | Intracranial hemorrhage (injury) | injuries & poisonings | 508 | 405,554 | 2,899 |
| 204.12 | Lymphoid leukemia, chronic | neoplasms | 506 | 404,466 | 3,989 |
| 300.13 | Phobia | mental disorders | 503 | 365,476 | 42,982 |
| 853 | Complication of colostomy or enterostomy | injuries & poisonings | 502 | 394,929 | 13,530 |
| 742.8 | Articular cartilage disorder | musculoskeletal | 500 | 402,633 | 5,828 |

Abbreviation: Phe-MR, phenome-wide Mendelian randomization.

**Table S4. Inverse-variance weighted MR analysis for the associations between blood metabolites and breast cancer.**

| **Metabolite** | **SNPs** | **OR (95% CI)** | ***P* value** |
| --- | --- | --- | --- |
| HDL-C | 273 | 1.09(1.06﹣1.12） | 9.67×10^-10^ |
| Total triglycerides | 261 | 0.95(0.92﹣0.98） | 5.23×10^-4^ |
| Apolipoprotein A1 | 243 | 1.08(1.04﹣1.11） | 1.16×10^-5^ |
| Glycoprotein acetyls | 205 | 1.02(0.98﹣1.05） | 0.32 |
| VLDL cholesterol | 202 | 0.98(0.95﹣1.01） | 0.22 |
| Linoleic acid | 197 | 1.02(0.98﹣1.06） | 0.3 |
| Sphingomyelins | 202 | 1.03(1.00﹣1.06） | 0.05 |
| Phosphatidylcholines | 195 | 1.03(1.00﹣1.07） | 0.06 |
| Phosphoglycerides | 187 | 1.03(1.00﹣1.07） | 0.07 |
| Apolipoprotein B | 183 | 0.98(0.96﹣1.01） | 0.24 |
| Total cholesterol | 183 | 1.00(0.97﹣1.04） | 0.82 |
| Docosahexaenoic acid | 174 | 1.03(0.99﹣1.07） | 0.13 |
| LDL cholesterol | 167 | 0.99(0.96﹣1.02） | 0.48 |
| Free cholesterol | 34 | 1.01(0.89﹣1.14） | 0.9 |
| 3-Hydroxybutyrate | 23 | 1.02(0.87﹣1.19） | 0.82 |
| Butyrylcarnitine | 3 | 0.90(0.42﹣1.96） | 0.8 |
| Carnitine | 20 | 1.58(0.90﹣2.75） | 0.11 |
| 4-androsten-3beta,17beta-diol disulfate 1 | 6 | 0.97(0.90﹣1.04） | 0.38 |
| Hexanoylcarnitine | 9 | 1.08(0.88﹣1.32） | 0.48 |
| Octanoylcarnitine | 8 | 1.09(0.98﹣1.23） | 0.12 |
| Androsterone sulfate | 8 | 0.98(0.89﹣1.08） | 0.72 |
| Epiandrosterone sulfate | 7 | 0.99(0.86﹣1.14） | 0.9 |
| 5alpha-androstan-3beta,17beta-diol disulfate | 5 | 0.99(0.84﹣1.15） | 0.86 |
| 3-dehydrocarnitine | 6 | 0.92(0.46﹣1.84） | 0.81 |
| 1-arachidonoylglycerophosphocholine | 5 | 1.1(0.91﹣1.34） | 0.31 |
| Arachidonate (20:4n6) | 5 | 1.04(0.84﹣1.28） | 0.72 |
| Cis-4-decenoyl carnitine | 5 | 1.10(0.94﹣1.29） | 0.25 |
| Decanoylcarnitine | 5 | 1.07(0.92﹣1.24） | 0.37 |
| 1-arachidonoylglycerophosphoinositol | 3 | 1.46(1.04﹣2.06） | 0.03 |
| Propionylcarnitine | 5 | 0.92(0.45﹣1.90） | 0.83 |
| Dihomo-linolenate (20:3n3 or n6) | 3 | 0.98(0.66﹣1.45） | 0.91 |
| 10-undecenoate (11:1n1) | 4 | 0.83(0.67﹣1.02） | 0.08 |
| Glycoproteins | 86 | 1.02(1.00﹣1.03） | 0.02 |
| Albumin | 12 | 0.98(0.93﹣1.04） | 0.56 |
| N-acetylornithine | 10 | 1.00(0.94﹣1.06） | 0.92 |
| Bradykinin, des-arg(9) | 5 | 0.97(0.91﹣1.03） | 0.29 |
| Gamma-glutamyltyrosine | 5 | 1.80(0.63﹣5.09） | 0.27 |
| HWESASXX | 3 | 0.85(0.69﹣1.03） | 0.1 |
| Urate | 5 | 1.74(1.24﹣2.44） | 1.33×10^-3^ |
| Uridine | 3 | 0.62(0.30﹣1.27） | 0.19 |
| X-12092 | 19 | 0.99(0.95﹣1.03） | 0.59 |
| X-12063 | 10 | 0.92(0.82﹣1.03） | 0.14 |
| X-11593--O-methylascorbate | 13 | 0.98(0.82﹣1.17） | 0.83 |
| X-12798 | 13 | 0.91(0.80﹣1.05） | 0.2 |
| X-11793--oxidized bilirubin | 9 | 1.00(0.90﹣1.12） | 0.98 |
| X-12728 | 9 | 1.00(0.98﹣1.01） | 0.52 |
| X-11787 | 9 | 1.39(1.08﹣1.79） | 0.01 |
| X-03056--N-[3-(2-Oxopyrrolidin-1-yl) propyl] acetamide | 8 | 1.13(0.93﹣1.39） | 0.22 |
| X-11530 | 8 | 0.98(0.90﹣1.08） | 0.73 |
| X-13431--nonanoylcarnitine | 7 | 1.11(1.01﹣1.22） | 0.03 |
| X-12510--2-aminooctanoic acid | 7 | 1.09(0.98﹣1.21） | 0.13 |
| X-11440 | 7 | 0.94(0.87﹣1.02） | 0.16 |
| X-11442 | 7 | 0.99(0.90﹣1.08） | 0.76 |
| X-11444 | 5 | 1.00(0.68﹣1.49） | 0.99 |
| X-11441 | 6 | 0.97(0.88﹣1.06） | 0.48 |
| X-08402 | 6 | 1.33(1.09﹣1.62） | 4.72×10^-3^ |
| X-12244--N-acetylcarnosine | 6 | 1.00(0.76﹣1.32） | 0.98 |
| X-11261 | 6 | 1.11(0.96﹣1.30） | 0.17 |
| X-12093 | 5 | 0.96(0.87﹣1.05） | 0.33 |
| X-12696 | 5 | 1.04(0.78﹣1.38） | 0.8 |
| X-03094 | 5 | 0.7(0.49﹣1.02） | 0.07 |
| X-11469 | 4 | 1.12(0.97﹣1.30） | 0.13 |
| X-12556 | 4 | 0.81(0.57﹣1.16） | 0.25 |
| X-08988 | 3 | 1.22(0.84﹣1.76） | 0.29 |
| X-02269 | 3 | 1.11(0.95﹣1.31） | 0.2 |
| X-12844 | 4 | 1.03(0.75﹣1.42） | 0.85 |
| X-14205--alpha-glutamyltyrosine | 3 | 1.05(0.87﹣1.27） | 0.63 |
| X-11792 | 3 | 0.96(0.88﹣1.04） | 0.32 |
| X-13435 | 3 | 1.04(0.79﹣1.38） | 0.78 |
| X-10510 | 3 | 1.40(1.01﹣1.94） | 0.04 |
| X-12850 | 3 | 0.94(0.65﹣1.37） | 0.74 |
| X-11315 | 3 | 0.53(0.26﹣1.05） | 0.07 |
| X-09789 | 3 | 0.82(0.67﹣1.00） | 0.05 |
| X-11204 | 3 | 1.71(0.80﹣3.66） | 0.17 |
| Acetone | 17 | 0.98(0.78﹣1.23） | 0.84 |
| Succinylcarnitine | 12 | 0.96(0.77﹣1.20） | 0.73 |
| Citrate | 7 | 1.10(0.70﹣1.73） | 0.67 |
| Acetate | 19 | 1.24(1.13﹣1.37） | 1.35×10^-5^ |
| Biliverdin | 9 | 1.00(0.91﹣1.09） | 0.97 |
| Bilirubin (Z,Z) | 8 | 0.99(0.93﹣1.05） | 0.74 |
| Bilirubin (E,E) | 7 | 0.99(0.92﹣1.08） | 0.9 |
| Bilirubin (E,Z or Z,E) | 4 | 0.93(0.81﹣1.06） | 0.26 |
| Pyruvate | 48 | 0.99(0.89﹣1.11） | 0.92 |
| Glucose | 34 | 1.01(0.89﹣1.14） | 0.9 |
| Lactate | 15 | 1.10(0.98﹣1.22） | 0.09 |
| 1,5-anhydroglucitol (1,5-AG) | 6 | 1.13(0.91﹣1.40） | 0.28 |
| Mannose | 6 | 1.14(0.92﹣1.42） | 0.23 |
| Erythronate | 3 | 1.15(0.58﹣2.27） | 0.69 |
| Glutamine | 65 | 1.01(0.92﹣1.10） | 0.86 |
| Creatinine | 95 | 1.03(0.95﹣1.11） | 0.52 |
| Valine | 52 | 1.05(0.97﹣1.13） | 0.22 |
| Histidine | 30 | 0.99(0.89﹣1.09） | 0.77 |
| Tryptophan | 18 | 2.05(0.95﹣4.45） | 0.07 |
| Isoleucine | 19 | 1.03(0.88﹣1.20） | 0.73 |
| Glutaroyl carnitine | 12 | 0.96(0.71﹣1.29） | 0.78 |
| Leucine | 12 | 1.35(0.69﹣2.63） | 0.38 |
| Glycine | 9 | 1.13(0.99﹣1.29） | 0.08 |
| Isobutyrylcarnitine | 8 | 1.09(0.97﹣1.23） | 0.16 |
| Proline | 7 | 0.88(0.69﹣1.11） | 0.28 |
| N-acetylglycine | 7 | 1.06(0.95﹣1.18） | 0.32 |
| Isovalerylcarnitine | 7 | 0.63(0.41﹣0.97） | 0.03 |
| Betaine | 5 | 0.75(0.54﹣1.05） | 0.09 |
| 4-acetamidobutanoate | 6 | 1.14(0.92﹣1.42） | 0.23 |
| Kynurenine | 4 | 1.10(0.79﹣1.51） | 0.58 |
| Pyroglutamine | 4 | 0.82(0.65﹣1.02） | 0.08 |
| Asparagine | 3 | 0.64(0.42﹣0.99） | 0.04 |
| Tryptophan betaine | 4 | 1.22(1.03﹣1.45） | 0.02 |
| Serine | 3 | 1.61(1.09﹣2.39） | 0.02 |
| Alpha-hydroxyisovalerate | 3 | 1.24(0.97﹣1.58） | 0.08 |
| 3-methyl-2-oxovalerate | 3 | 1.14(0.29﹣4.42） | 0.85 |
| Tyrosine | 4 | 2.77(0.66﹣11.69） | 0.17 |
| Citrulline | 4 | 0.72(0.38﹣1.37） | 0.32 |

Odds ratios (ORs) with their 95% confidence intervals (CIs) represent the association estimates with the risk of breast cancer of per 1-SD increase in blood metabolite levels.

Significant threshold in stage 1 was set at *P* <0.05/112=4.46×10^-4^ (Bonferroni-corrected significance threshold calculated as 0.05 divided by 112 [for 112 blood metabolites]).

Abbreviations: MR, Mendelian randomization; SNPs, single nucleotide polymorphisms.

**Table S5.** **Sensitivity analyses for significant blood metabolites.**

| **Metabolite** | **SNPs** | **Weighted median** | | **MR-RAPS** | | **MR-Egger Intercept** |
| --- | --- | --- | --- | --- | --- | --- |
|  |  | **OR (95% CI)** | ***P* value** | **OR (95% CI)** | ***P* value** | ***P* value** |
| HDL-C | 273 | 1.09(1.05-1.13) | 2.07×10^-5^ | 1.09(1.07-1.11) | 3.77×10^-15^ | 0.18 |
| Apolipoprotein A1 | 243 | 1.07(1.03-1.12) | 9.73×10^-4^ | 1.08(1.05-1.10) | 4.39×10^-10^ | 0.65 |
| Acetate | 19 | 1.33(1.15-1.52) | 7.54×10^-5^ | 1.25(1.13-1.38) | 1.45×10^-5^ | 0.13 |

Odds ratios (ORs) with their 95% confidence intervals (CIs) represent the association estimates with the risk of breast cancer of per 1-SD increase in HDL-C, Apolipoprotein A1 and acetate levels, respectively.

Significant threshold in stage 1 was set at *P* <0.05/112=4.46×10^-4^ (Bonferroni-corrected significance threshold calculated as 0.05 divided by 112 [for 112 blood metabolites]).

Abbreviations: MR-RAPS, Mendelian randomization robust adjusted profile score; SNPs, single nucleotide polymorphisms.

**Table S6. Phe-MR analysis for the associations between HDL-C and 679 diseases using the inverse-variance weighted method.**

| **PheCode** | **Phenotype Description** | **Disease Category** | **SNPs** | **OR (95% CI)** | ***P* value** |
| --- | --- | --- | --- | --- | --- |
| 411 | Ischemic Heart Disease | circulatory system | 272 | 0.83 (0.79-0.87) | 3.50×10^-13^ |
| 411.4 | Coronary atherosclerosis | circulatory system | 272 | 0.81 (0.76-0.86) | 4.13×10^-11^ |
| 411.3 | Angina pectoris | circulatory system | 272 | 0.83 (0.78-0.88) | 5.63×10^-11^ |
| 1001 | Foreign body injury | injuries & poisonings | 272 | 0.65 (0.57-0.74) | 1.79×10^-10^ |
| 250.2 | Type 2 diabetes | endocrine/metabolic | 272 | 0.83 (0.79-0.88) | 6.66×10^-10^ |
| 444 | Arterial embolism and thrombosis | circulatory system | 272 | 0.59 (0.50-0.70) | 7.01×10^-10^ |
| 411.2 | Myocardial infarction | circulatory system | 272 | 0.81 (0.75-0.86) | 9.30×10^-10^ |
| 442.11 | Abdominal aortic aneurysm | circulatory system | 272 | 0.62 (0.53-0.72) | 1.04×10^-9^ |
| 426 | Cardiac conduction disorders | circulatory system | 272 | 0.83 (0.78-0.88) | 4.16×10^-9^ |
| 250 | Diabetes mellitus | endocrine/metabolic | 272 | 0.85 (0.80-0.90) | 1.05×10^-8^ |
| 440.2 | Atherosclerosis of the extremities | circulatory system | 272 | 0.62 (0.52-0.74) | 1.17×10^-7^ |
| 444.1 | Arterial embolism and thrombosis of lower extremity artery | circulatory system | 272 | 0.59 (0.48-0.72) | 1.38×10^-7^ |
| 411.1 | Unstable angina (intermediate coronary syndrome) | circulatory system | 272 | 0.81 (0.74-0.87) | 1.73×10^-7^ |
| 440 | Atherosclerosis | circulatory system | 272 | 0.71 (0.62-0.81) | 1.84×10^-7^ |
| 555 | Inflammatory bowel disease and other gastroenteritis and colitis | digestive | 272 | 0.83 (0.78-0.89) | 3.96×10^-7^ |
| 442.1 | Aortic aneurysm | circulatory system | 272 | 0.71 (0.62-0.81) | 7.11×10^-7^ |
| 401.1 | Essential hypertension | circulatory system | 272 | 0.90 (0.86-0.94) | 7.42×10^-7^ |
| 411.8 | Other chronic ischemic heart disease, unspecified | circulatory system | 272 | 0.86 (0.81-0.91) | 7.80×10^-7^ |
| 381.1 | Otitis media | sense organs | 272 | 0.77 (0.69-0.85) | 1.77×10^-6^ |
| 572 | Ascites (non malignant) | digestive | 272 | 0.75 (0.67-0.84) | 1.83×10^-6^ |
| 204 | Leukemia | neoplasms | 272 | 1.35 (1.19-1.53) | 3.15×10^-6^ |
| 599.4 | Urinary incontinence | genitourinary | 272 | 0.89 (0.84-0.93) | 4.24×10^-6^ |
| 381.11 | Suppurative and unspecified otitis media | sense organs | 272 | 0.69 (0.59-0.81) | 5.27×10^-6^ |
| 850 | Hemorrhage or hematoma complicating a procedure | injuries & poisonings | 272 | 0.85 (0.79-0.91) | 6.85×10^-6^ |
| 225 | Benign neoplasm of brain and other parts of nervous system | neoplasms | 272 | 1.45 (1.23-1.70) | 7.45×10^-6^ |
| 225.1 | Benign neoplasm of brain, cranial nerves, meninges | neoplasms | 272 | 1.46 (1.24-1.73) | 8.71×10^-6^ |
| 381 | Otitis media and Eustachian tube disorders | sense organs | 272 | 0.80 (0.73-0.89) | 1.14×10^-5^ |
| 681.5 | Cellulitis and abscess of leg, except foot | dermatologic | 272 | 0.87 (0.82-0.93) | 1.43×10^-5^ |
| 585 | Renal failure | genitourinary | 272 | 0.88 (0.82-0.93) | 1.69×10^-5^ |
| 443.9 | Peripheral vascular disease, unspecified | circulatory system | 272 | 0.80 (0.72-0.88) | 1.83×10^-5^ |
| 447 | Other disorders of arteries and arterioles | circulatory system | 272 | 0.76 (0.67-0.86) | 2.23×10^-5^ |
| 1010 | Other tests | symptoms | 272 | 0.88 (0.83-0.93) | 2.42×10^-5^ |
| 474.1 | Acute tonsillitis | respiratory | 272 | 0.68 (0.56-0.82) | 4.27×10^-5^ |
| 477 | Epistaxis or throat hemorrhage | respiratory | 272 | 0.82 (0.75-0.90) | 4.29×10^-5^ |
| 611 | Abnormal findings on mammogram or breast exam | genitourinary | 272 | 1.28 (1.13-1.44) | 4.87×10^-5^ |
| 340 | Migraine | neurological | 272 | 0.82 (0.74-0.90) | 4.99×10^-5^ |
| 611.3 | Lump or mass in breast | genitourinary | 272 | 1.28 (1.13-1.44) | 5.56×10^-5^ |
| 443 | Peripheral vascular disease | circulatory system | 272 | 0.84 (0.77-0.92) | 5.65×10^-5^ |
| 681.3 | Cellulitis and abscess of arm/hand | dermatologic | 272 | 0.88 (0.82-0.94) | 5.88×10^-5^ |
| 681.6 | Cellulitis and abscess of foot, toe | dermatologic | 272 | 0.88 (0.82-0.94) | 6.61×10^-5^ |
| 523.31 | Acute periodontitis | digestive | 272 | 1.44 (1.20-1.73) | 6.94×10^-5^ |
| 722.9 | Other and unspecified disc disorder | musculoskeletal | 272 | 0.86 (0.79-0.92) | 7.63×10^-5^ |
| 555.2 | Ulcerative colitis | digestive | 272 | 0.85 (0.78-0.92) | 7.64×10^-5^ |
| 442 | Other aneurysm | circulatory system | 272 | 0.79 (0.70-0.89) | 1.14×10^-4^ |
| 333 | Extrapyramidal disease and abnormal movement disorders | neurological | 272 | 0.74 (0.63-0.86) | 1.17×10^-4^ |
| 426.9 | Cardiac pacemaker/device in situ | circulatory system | 272 | 0.82 (0.74-0.91) | 1.17×10^-4^ |
| 250.7 | Diabetic retinopathy | endocrine/metabolic | 272 | 0.75 (0.65-0.87) | 1.28×10^-4^ |
| 433.1 | Occlusion and stenosis of precerebral arteries | circulatory system | 272 | 0.77 (0.67-0.88) | 1.36×10^-4^ |
| 686.1 | Carbuncle and furuncle | dermatologic | 272 | 0.83 (0.75-0.91) | 1.37×10^-4^ |
| 426.3 | Bundle branch block | circulatory system | 272 | 0.86 (0.79-0.93) | 1.82×10^-4^ |
| 509 | Respiratory failure, insufficiency, arrest | respiratory | 272 | 0.84 (0.76-0.92) | 2.03×10^-4^ |
| 335 | Multiple sclerosis | neurological | 272 | 1.27 (1.12-1.44) | 2.26×10^-4^ |
| 430.2 | Intracerebral hemorrhage | circulatory system | 272 | 0.72 (0.60-0.86) | 2.37×10^-4^ |
| 585.1 | Acute renal failure | genitourinary | 272 | 0.88 (0.82-0.94) | 3.29×10^-4^ |
| 306 | Other mental disorder | mental disorders | 272 | 0.94 (0.90-0.97) | 3.32×10^-4^ |
| 558 | Noninfectious gastroenteritis | digestive | 272 | 0.92 (0.89-0.97) | 3.55×10^-4^ |
| 1002 | Symptoms concerning nutrition, metabolism, and development | symptoms | 272 | 0.89 (0.84-0.95) | 4.20×10^-4^ |
| 681 | Superficial cellulitis and abscess | dermatologic | 272 | 0.91 (0.86-0.96) | 4.56×10^-4^ |
| 555.1 | Regional enteritis | digestive | 272 | 0.82 (0.73-0.92) | 4.81×10^-4^ |
| 550.5 | Ventral hernia | digestive | 272 | 0.87 (0.80-0.94) | 4.85×10^-4^ |
| 418 | Nonspecific chest pain | circulatory system | 272 | 0.95 (0.92-0.98) | 5.65×10^-4^ |
| 961 | Poisoning by other antifinfectives | injuries & poisonings | 272 | 1.26 (1.10-1.43) | 6.54×10^-4^ |
| 459.9 | Circulatory disease NEC | circulatory system | 272 | 0.93 (0.89-0.97) | 6.72×10^-4^ |
| 428 | Congestive heart failure; nonhypertensive | circulatory system | 272 | 0.89 (0.83-0.95) | 6.76×10^-4^ |
| 447.1 | Stricture of artery | circulatory system | 272 | 0.76 (0.65-0.89) | 6.95×10^-4^ |
| 1019 | Other ill-defined and unknown causes of morbidity and mortality | symptoms | 272 | 0.94 (0.90-0.97) | 7.14×10^-4^ |
| 426.32 | Left bundle branch block | circulatory system | 272 | 0.83 (0.74-0.92) | 7.45×10^-4^ |
| 596.5 | Functional disorders of bladder | genitourinary | 272 | 0.82 (0.73-0.92) | 7.52×10^-4^ |
| 510 | Other diseases of lung | respiratory | 272 | 0.75 (0.64-0.89) | 8.19×10^-4^ |
| 204.12 | Lymphoid leukemia, chronic | neoplasms | 272 | 1.48 (1.17-1.86) | 8.32×10^-4^ |
| 459 | Other disorders of circulatory system | circulatory system | 272 | 0.93 (0.89-0.97) | 8.60×10^-4^ |
| 368 | Visual disturbances | sense organs | 272 | 0.87 (0.80-0.95) | 8.97×10^-4^ |
| 556.1 | Ulceration of intestine | digestive | 272 | 0.74 (0.62-0.89) | 9.98×10^-4^ |
| 441 | Vascular insufficiency of intestine | circulatory system | 272 | 0.72 (0.59-0.88) | 1.01×10^-3^ |
| 272.1 | Hyperlipidemia | endocrine/metabolic | 272 | 0.90 (0.84-0.96) | 1.07×10^-3^ |
| 272 | Disorders of lipid metabolism | endocrine/metabolic | 272 | 0.90 (0.84-0.96) | 1.07×10^-3^ |
| 560.3 | Peritoneal or intestinal adhesions | digestive | 272 | 0.76 (0.65-0.90) | 1.11×10^-3^ |
| 411.9 | Other acute and subacute forms of ischemic heart disease | circulatory system | 272 | 0.80 (0.70-0.91) | 1.20×10^-3^ |
| 495 | Asthma | respiratory | 272 | 0.94 (0.90-0.97) | 1.25×10^-3^ |
| 394 | Rheumatic disease of the heart valves | circulatory system | 272 | 0.88 (0.82-0.95) | 1.31×10^-3^ |
| 819 | Skull and face fracture and other intercranial injury | injuries & poisonings | 272 | 1.15 (1.06-1.25) | 1.35×10^-3^ |
| 285 | Other anemias | hematopoietic | 272 | 0.93 (0.89-0.97) | 1.38×10^-3^ |
| 597.1 | Urethral stricture (not specified as infectious) | genitourinary | 272 | 0.88 (0.81-0.95) | 1.44×10^-3^ |
| 385 | Other disorders of middle ear and mastoid | sense organs | 272 | 0.77 (0.65-0.90) | 1.51×10^-3^ |
| 426.2 | Atrioventricular [AV] block | circulatory system | 272 | 0.85 (0.77-0.94) | 1.53×10^-3^ |
| 112 | Candidiasis | infectious diseases | 272 | 0.85 (0.77-0.94) | 1.54×10^-3^ |
| 531.3 | Duodenal ulcer | digestive | 272 | 1.15 (1.05-1.25) | 1.55×10^-3^ |
| 571 | Chronic liver disease and cirrhosis | digestive | 272 | 0.86 (0.78-0.94) | 1.56×10^-3^ |
| 276.14 | Hypopotassemia | endocrine/metabolic | 272 | 0.82 (0.73-0.93) | 1.58×10^-3^ |
| 520 | Disorders of tooth development | digestive | 272 | 1.16 (1.06-1.28) | 1.67×10^-3^ |
| 172.1 | Melanomas of skin, dx or hx | neoplasms | 272 | 0.87 (0.79-0.95) | 1.75×10^-3^ |
| 172.11 | Melanomas of skin | neoplasms | 272 | 0.87 (0.79-0.95) | 1.75×10^-3^ |
| 458 | Hypotension | circulatory system | 272 | 0.91 (0.85-0.96) | 1.78×10^-3^ |
| 80 | Postoperative infection | infectious diseases | 272 | 0.89 (0.82-0.96) | 1.92×10^-3^ |
| 272.11 | Hypercholesterolemia | endocrine/metabolic | 272 | 0.90 (0.84-0.96) | 1.93×10^-3^ |
| 411.41 | Aneurysm and dissection of heart | circulatory system | 272 | 0.76 (0.64-0.90) | 1.96×10^-3^ |
| 204.1 | Lymphoid leukemia | neoplasms | 272 | 1.42 (1.14-1.77) | 1.96×10^-3^ |
| 745 | Pain in joint | musculoskeletal | 272 | 0.91 (0.86-0.97) | 2.16×10^-3^ |
| 771 | Musculoskeletal symptoms referable to limbs | symptoms | 272 | 0.91 (0.85-0.97) | 2.27×10^-3^ |
| 519 | Other diseases of respiratory system, not elsewhere classified | respiratory | 272 | 0.93 (0.88-0.97) | 2.38×10^-3^ |
| 597 | Other disorders of urethra and urinary tract | genitourinary | 272 | 0.88 (0.82-0.96) | 2.40×10^-3^ |
| 216 | Benign neoplasm of skin | neoplasms | 272 | 1.09 (1.03-1.15) | 2.48×10^-3^ |
| 426.91 | Cardiac pacemaker in situ | circulatory system | 272 | 0.85 (0.76-0.94) | 2.55×10^-3^ |
| 709.2 | Sicca syndrome | dermatologic | 272 | 0.70 (0.56-0.88) | 2.56×10^-3^ |
| 979 | Adverse drug events and drug allergies | injuries & poisonings | 272 | 1.31 (1.10-1.56) | 2.70×10^-3^ |
| 585.3 | Chronic renal failure [CKD] | genitourinary | 272 | 0.87 (0.79-0.95) | 2.70×10^-3^ |
| 573.7 | Abnormal results of function study of liver | digestive | 272 | 0.89 (0.82-0.96) | 2.71×10^-3^ |
| 204.4 | Multiple myeloma | neoplasms | 272 | 1.35 (1.11-1.65) | 2.83×10^-3^ |
| 521 | Diseases of hard tissues of teeth | digestive | 272 | 1.14 (1.04-1.24) | 2.89×10^-3^ |
| 709 | Diffuse diseases of connective tissue | dermatologic | 272 | 0.88 (0.81-0.96) | 2.94×10^-3^ |
| 743.2 | Pathologic fracture | musculoskeletal | 272 | 1.36 (1.11-1.67) | 3.33×10^-3^ |
| 854 | Complications of cardiac/vascular device, implant, and graft | injuries & poisonings | 272 | 0.82 (0.72-0.94) | 3.33×10^-3^ |
| 520.2 | Disturbances in tooth eruption | digestive | 272 | 1.15 (1.05-1.27) | 3.44×10^-3^ |
| 785 | Abdominal pain | symptoms | 272 | 0.96 (0.94-0.99) | 3.45×10^-3^ |
| 368.1 | Amblyopia | sense organs | 272 | 0.74 (0.61-0.91) | 3.47×10^-3^ |
| 523.3 | Periodontitis (acute or chronic) | digestive | 272 | 1.22 (1.07-1.39) | 3.47×10^-3^ |
| 990 | Effects radiation NOS | injuries & poisonings | 272 | 1.13 (1.04-1.23) | 3.51×10^-3^ |
| 251.1 | Hypoglycemia | endocrine/metabolic | 272 | 0.80 (0.68-0.93) | 3.59×10^-3^ |
| 695.7 | Prurigo and Lichen | dermatologic | 272 | 1.28 (1.08-1.51) | 3.73×10^-3^ |
| 727 | Other disorders of synovium, tendon, and bursa | musculoskeletal | 272 | 0.92 (0.87-0.97) | 3.75×10^-3^ |
| 720 | Spinal stenosis | musculoskeletal | 272 | 0.88 (0.81-0.96) | 3.83×10^-3^ |
| 401.2 | Hypertensive heart and/or renal disease | circulatory system | 272 | 0.85 (0.76-0.95) | 4.03×10^-3^ |
| 686 | Other local infections of skin and subcutaneous tissue | dermatologic | 272 | 0.90 (0.84-0.97) | 4.48×10^-3^ |
| 550.4 | Umbilical hernia | digestive | 272 | 0.89 (0.83-0.97) | 4.58×10^-3^ |
| 345.1 | Epilepsy | neurological | 272 | 0.78 (0.66-0.93) | 5.11×10^-3^ |
| 747 | Cardiac and circulatory congenital anomalies | congenital anomalies | 272 | 0.88 (0.81-0.96) | 5.14×10^-3^ |
| 521.1 | Dental caries | digestive | 272 | 1.13 (1.04-1.23) | 5.18×10^-3^ |
| 426.21 | First degree AV block | circulatory system | 272 | 0.81 (0.70-0.94) | 5.34×10^-3^ |
| 519.8 | Other diseases of respiratory system, NEC | respiratory | 272 | 0.93 (0.89-0.98) | 5.60×10^-3^ |
| 580 | Nephritis; nephrosis; renal sclerosis | genitourinary | 272 | 0.84 (0.75-0.95) | 5.88×10^-3^ |
| 496 | Chronic airway obstruction | respiratory | 272 | 0.92 (0.87-0.98) | 6.03×10^-3^ |
| 8 | Intestinal infection | infectious diseases | 272 | 0.93 (0.89-0.98) | 6.49×10^-3^ |
| 803.1 | Fracture of humerus | injuries & poisonings | 272 | 1.17 (1.04-1.31) | 6.58×10^-3^ |
| 250.24 | Type 2 diabetes with neurological manifestations | endocrine/metabolic | 272 | 0.76 (0.63-0.93) | 6.59×10^-3^ |
| 395 | Heart valve disorders | circulatory system | 272 | 0.90 (0.84-0.97) | 6.71×10^-3^ |
| 1009 | Injury, NOS | injuries & poisonings | 272 | 0.93 (0.88-0.98) | 6.79×10^-3^ |
| 728.7 | Fasciitis | musculoskeletal | 272 | 1.11 (1.03-1.20) | 7.49×10^-3^ |
| 573 | Other disorders of liver | digestive | 272 | 0.92 (0.86-0.98) | 7.51×10^-3^ |
| 782 | Symptoms involving skin and other integumentary tissue | symptoms | 272 | 0.85 (0.76-0.96) | 7.61×10^-3^ |
| 580.14 | Chronic glomerulonephritis, NOS | genitourinary | 272 | 0.80 (0.69-0.94) | 7.82×10^-3^ |
| 401 | Hypertension | circulatory system | 272 | 1.06 (1.02-1.11) | 7.91×10^-3^ |
| 575.7 | Other disorders of gallbladder | digestive | 272 | 0.85 (0.75-0.96) | 8.11×10^-3^ |
| 807 | Fracture of ribs | injuries & poisonings | 272 | 0.80 (0.68-0.94) | 8.71×10^-3^ |
| 260 | Protein-calorie malnutrition | endocrine/metabolic | 272 | 0.83 (0.72-0.95) | 8.71×10^-3^ |
| 251 | Other disorders of pancreatic internal secretion | endocrine/metabolic | 272 | 0.82 (0.70-0.95) | 8.84×10^-3^ |
| 728.71 | Contracture of palmar fascia [Dupuytren's disease] | musculoskeletal | 272 | 1.11 (1.03-1.21) | 9.03×10^-3^ |
| 250.23 | Type 2 diabetes with ophthalmic manifestations | endocrine/metabolic | 272 | 0.82 (0.70-0.95) | 9.27×10^-3^ |
| 380 | Disorders of external ear | sense organs | 272 | 0.85 (0.75-0.96) | 9.35×10^-3^ |
| 292 | Neurological disorders | mental disorders | 272 | 0.91 (0.85-0.98) | 9.38×10^-3^ |
| 479 | Other upper respiratory disease | respiratory | 272 | 0.91 (0.84-0.98) | 9.61×10^-3^ |
| 153 | Colorectal cancer | neoplasms | 272 | 1.10 (1.02-1.18) | 9.66×10^-3^ |
| 285.2 | Anemia of chronic disease | hematopoietic | 272 | 0.79 (0.67-0.95) | 9.99×10^-3^ |
| 159 | Malignant neoplasm of other and ill-defined sites within the digestive organs and peritoneum | neoplasms | 272 | 1.09 (1.02-1.17) | 0.01 |
| 384 | Other disorders of tympanic membrane | sense organs | 272 | 0.85 (0.75-0.96) | 0.01 |
| 473 | Diseases of the larynx and vocal cords | respiratory | 272 | 0.89 (0.81-0.97) | 0.01 |
| 599 | Other symptoms/disorders or the urinary system | genitourinary | 272 | 0.96 (0.93-0.99) | 0.01 |
| 153.3 | Malignant neoplasm of rectum, rectosigmoid junction, and anus | neoplasms | 272 | 1.14 (1.03-1.26) | 0.01 |
| 496.21 | Obstructive chronic bronchitis | respiratory | 272 | 0.88 (0.79-0.97) | 0.01 |
| 569 | Other disorders of intestine | digestive | 272 | 0.90 (0.83-0.98) | 0.01 |
| 296 | Mood disorders | mental disorders | 272 | 0.95 (0.91-0.99) | 0.01 |
| 157 | Pancreatic cancer | neoplasms | 272 | 0.78 (0.65-0.95) | 0.01 |
| 474.2 | Chronic tonsillitis and adenoiditis | respiratory | 272 | 0.84 (0.73-0.96) | 0.01 |
| 575 | Other biliary tract disease | digestive | 272 | 0.90 (0.83-0.98) | 0.01 |
| 747.1 | Cardiac congenital anomalies | congenital anomalies | 272 | 0.89 (0.81-0.98) | 0.01 |
| 724.9 | Other unspecified back disorders | musculoskeletal | 272 | 0.86 (0.77-0.97) | 0.01 |
| 709.7 | Unspecified diffuse connective tissue disease | dermatologic | 272 | 0.89 (0.82-0.98) | 0.01 |
| 532 | Dysphagia | digestive | 272 | 0.93 (0.88-0.99) | 0.01 |
| 350 | Abnormal movement | neurological | 272 | 0.89 (0.82-0.98) | 0.01 |
| 291 | Other specified nonpsychotic and/or transient mental disorders | mental disorders | 272 | 1.26 (1.05-1.51) | 0.02 |
| 79 | Viral infection | infectious diseases | 272 | 0.90 (0.82-0.98) | 0.02 |
| 509.2 | Respiratory insufficiency | respiratory | 272 | 0.87 (0.78-0.98) | 0.02 |
| 38 | Septicemia | infectious diseases | 272 | 0.91 (0.85-0.98) | 0.02 |
| 529 | Diseases and other conditions of the tongue | digestive | 272 | 0.85 (0.75-0.97) | 0.02 |
| 296.2 | Depression | mental disorders | 272 | 0.95 (0.91-0.99) | 0.02 |
| 575.2 | Obstruction of bile duct | digestive | 272 | 0.82 (0.69-0.97) | 0.02 |
| 401.22 | Hypertensive chronic kidney disease | circulatory system | 272 | 0.87 (0.77-0.98) | 0.02 |
| 782.3 | Edema | symptoms | 272 | 0.87 (0.78-0.98) | 0.02 |
| 202.2 | Non-Hodgkins lymphoma | neoplasms | 272 | 1.14 (1.02-1.27) | 0.02 |
| 722 | Intervertebral disc disorders | musculoskeletal | 272 | 0.94 (0.88-0.99) | 0.02 |
| 290.2 | Delirium due to conditions classified elsewhere | mental disorders | 272 | 0.81 (0.67-0.97) | 0.02 |
| 367.9 | Blindness and low vision | sense organs | 272 | 0.82 (0.69-0.97) | 0.02 |
| 458.1 | Orthostatic hypotension | circulatory system | 272 | 0.86 (0.76-0.98) | 0.02 |
| 564.8 | Abnormal findings on exam of gastrointestinal tract/ abdominal area | digestive | 272 | 0.88 (0.78-0.98) | 0.02 |
| 741.4 | Joint effusions | musculoskeletal | 272 | 0.87 (0.77-0.98) | 0.02 |
| 338 | Pain | symptoms | 272 | 0.83 (0.70-0.98) | 0.02 |
| 613.1 | Inflammatory disease of breast | genitourinary | 272 | 0.82 (0.69-0.97) | 0.02 |
| 472 | Chronic pharyngitis and nasopharyngitis | respiratory | 272 | 1.19 (1.02-1.38) | 0.02 |
| 276.5 | Hypovolemia | endocrine/metabolic | 272 | 0.90 (0.83-0.99) | 0.03 |
| 733.8 | Malunion and nonunion of fracture | musculoskeletal | 272 | 1.16 (1.02-1.32) | 0.03 |
| 8.5 | Bacterial enteritis | infectious diseases | 272 | 0.91 (0.83-0.99) | 0.03 |
| 296.1 | Bipolar | mental disorders | 272 | 0.84 (0.71-0.98) | 0.03 |
| 742.8 | Articular cartilage disorder | musculoskeletal | 272 | 0.78 (0.62-0.98) | 0.03 |
| 802 | Fracture of pelvis | injuries & poisonings | 272 | 1.21 (1.02-1.44) | 0.03 |
| 420.3 | Endocarditis | circulatory system | 272 | 0.82 (0.69-0.98) | 0.03 |
| 729.1 | Rheumatism, unspecified and fibrositis | musculoskeletal | 272 | 1.20 (1.02-1.41) | 0.03 |
| 764 | Sciatica | symptoms | 272 | 0.90 (0.82-0.99) | 0.03 |
| 244.4 | Hypothyroidism NOS | endocrine/metabolic | 272 | 0.92 (0.86-0.99) | 0.03 |
| 202 | Cancer of other lymphoid, histiocytic tissue | neoplasms | 272 | 1.11 (1.01-1.23) | 0.03 |
| 560 | Intestinal obstruction without mention of hernia | digestive | 272 | 0.92 (0.86-0.99) | 0.03 |
| 560.4 | Other intestinal obstruction | digestive | 272 | 0.92 (0.85-0.99) | 0.03 |
| 525 | Other diseases of the teeth and supporting structures | digestive | 272 | 0.90 (0.82-0.99) | 0.03 |
| 704 | Diseases of hair and hair follicles | dermatologic | 272 | 0.93 (0.87-0.99) | 0.03 |
| 791 | Gangrene | symptoms | 272 | 0.81 (0.66-0.98) | 0.03 |
| 241 | Nontoxic nodular goiter | endocrine/metabolic | 272 | 1.16 (1.01-1.33) | 0.04 |
| 339 | Other headache syndromes | neurological | 272 | 0.94 (0.90-1.00) | 0.04 |
| 574 | Cholelithiasis and cholecystitis | digestive | 272 | 0.94 (0.88-1.00) | 0.04 |
| 274 | Gout and other crystal arthropathies | endocrine/metabolic | 272 | 0.91 (0.83-0.99) | 0.04 |
| 496.2 | Chronic bronchitis | respiratory | 272 | 0.91 (0.82-0.99) | 0.04 |
| 292.1 | Aphasia/speech disturbance | mental disorders | 272 | 0.88 (0.78-0.99) | 0.04 |
| 385.3 | Cholesteatoma | sense organs | 272 | 0.82 (0.68-0.99) | 0.04 |
| 394.2 | Mitral valve disease | circulatory system | 272 | 0.91 (0.84-1.00) | 0.04 |
| 252.1 | Hyperparathyroidism | endocrine/metabolic | 272 | 0.84 (0.71-0.99) | 0.04 |
| 362 | Other retinal disorders | sense organs | 272 | 0.92 (0.86-1.00) | 0.04 |
| 594.2 | Calculus of lower urinary tract | genitourinary | 272 | 0.84 (0.71-0.99) | 0.04 |
| 681.7 | Cellulitis and abscess of trunk | dermatologic | 272 | 0.80 (0.65-0.99) | 0.04 |
| 771.1 | Swelling of limb | symptoms | 272 | 0.93 (0.86-1.00) | 0.04 |
| 502 | Postinflammatory pulmonary fibrosis | respiratory | 272 | 1.17 (1.00-1.37) | 0.04 |
| 574.2 | Calculus of bile duct | digestive | 272 | 0.90 (0.81-1.00) | 0.05 |
| 596 | Other disorders of bladder | genitourinary | 272 | 0.95 (0.91-1.00) | 0.05 |
| 255 | Disorders of adrenal glands | endocrine/metabolic | 272 | 1.21 (1.00-1.45) | 0.05 |
| 429.2 | Abnormal function study of cardiovascular system | circulatory system | 272 | 0.81 (0.66-1.00) | 0.05 |
| 244.1 | Secondary hypothyroidism | endocrine/metabolic | 272 | 1.15 (1.00-1.32) | 0.05 |
| 740.2 | Osteoarthrosis, generalized | musculoskeletal | 272 | 0.83 (0.68-1.00) | 0.05 |
| 580.1 | Glomerulonephritis | genitourinary | 272 | 0.86 (0.75-1.00) | 0.05 |
| 276.1 | Electrolyte imbalance | endocrine/metabolic | 272 | 0.93 (0.86-1.00) | 0.05 |
| 172.3 | Carcinoma in situ of skin | neoplasms | 272 | 0.83 (0.70-1.00) | 0.05 |
| 241.2 | Nontoxic multinodular goiter | endocrine/metabolic | 272 | 0.83 (0.70-1.00) | 0.05 |
| 281.11 | Pernicious anemia | hematopoietic | 272 | 0.83 (0.70-1.00) | 0.05 |
| 577 | Diseases of pancreas | digestive | 272 | 0.91 (0.83-1.00) | 0.05 |
| 278 | Overweight, obesity and other hyperalimentation | endocrine/metabolic | 272 | 0.94 (0.89-1.00) | 0.05 |
| 574.1 | Cholelithiasis | digestive | 272 | 0.94 (0.88-1.00) | 0.05 |
| 276 | Disorders of fluid, electrolyte, and acid-base balance | endocrine/metabolic | 272 | 0.95 (0.90-1.00) | 0.05 |
| 578.9 | Hemorrhage of gastrointestinal tract | digestive | 272 | 0.93 (0.87-1.00) | 0.05 |
| 274.1 | Gout | endocrine/metabolic | 272 | 0.91 (0.82-1.00) | 0.05 |
| 350.1 | Abnormal involuntary movements | neurological | 272 | 0.86 (0.73-1.00) | 0.05 |
| 211 | Benign neoplasm of other parts of digestive system | neoplasms | 272 | 0.94 (0.88-1.00) | 0.05 |
| 506 | Empyema and pneumothorax | respiratory | 272 | 1.14 (1.00-1.31) | 0.05 |
| 471 | Nasal polyps | respiratory | 272 | 1.09 (1.00-1.20) | 0.05 |
| 451 | Phlebitis and thrombophlebitis | circulatory system | 272 | 0.92 (0.84-1.00) | 0.05 |
| 565.1 | Anal and rectal polyp | digestive | 272 | 0.95 (0.90-1.00) | 0.05 |
| 994 | Sepsis and SIRS | infectious diseases | 272 | 0.92 (0.84-1.00) | 0.05 |
| 994.2 | Sepsis | infectious diseases | 272 | 0.92 (0.84-1.00) | 0.05 |
| 528 | Diseases of the oral soft tissues, excluding lesions specific for gingiva and tongue | digestive | 272 | 0.93 (0.86-1.00) | 0.05 |
| 574.3 | Cholecystitis without cholelithiasis | digestive | 272 | 0.90 (0.81-1.00) | 0.05 |
| 594.3 | Calculus of ureter | genitourinary | 272 | 1.10 (1.00-1.21) | 0.05 |
| 803 | Fracture of upper limb | injuries & poisonings | 272 | 1.06 (1.00-1.12) | 0.06 |
| 395.1 | Nonrheumatic mitral valve disorders | circulatory system | 272 | 0.92 (0.84-1.00) | 0.06 |
| 418.1 | Precordial pain | circulatory system | 272 | 0.93 (0.86-1.00) | 0.06 |
| 386 | Vertiginous syndromes and other disorders of vestibular system | sense organs | 272 | 0.94 (0.89-1.00) | 0.06 |
| 531 | Peptic ulcer (excl. esophageal) | digestive | 272 | 1.05 (1.00-1.11) | 0.06 |
| 451.2 | Phlebitis and thrombophlebitis of lower extremities | circulatory system | 272 | 0.92 (0.84-1.00) | 0.06 |
| 564 | Functional digestive disorders | digestive | 272 | 0.97 (0.93-1.00) | 0.06 |
| 706.2 | Sebaceous cyst | dermatologic | 272 | 0.95 (0.91-1.00) | 0.06 |
| 591 | Urinary tract infection | genitourinary | 272 | 0.96 (0.92-1.00) | 0.06 |
| 331 | Other cerebral degenerations | neurological | 272 | 0.88 (0.76-1.01) | 0.06 |
| 332 | Parkinson's disease | neurological | 272 | 0.84 (0.70-1.01) | 0.06 |
| 727.5 | Rupture of synovium | musculoskeletal | 272 | 0.83 (0.69-1.01) | 0.06 |
| 289.4 | Lymphadenitis | hematopoietic | 272 | 0.92 (0.84-1.00) | 0.06 |
| 427.4 | Cardiac arrest and ventricular fibrillation | circulatory system | 272 | 0.88 (0.77-1.01) | 0.07 |
| 789 | Nausea and vomiting | symptoms | 272 | 0.96 (0.92-1.00) | 0.07 |
| 244 | Hypothyroidism | endocrine/metabolic | 272 | 0.94 (0.87-1.00) | 0.07 |
| 345.3 | Convulsions | neurological | 272 | 0.91 (0.81-1.01) | 0.07 |
| 428.2 | Heart failure NOS | circulatory system | 272 | 0.93 (0.86-1.01) | 0.07 |
| 586.2 | Cyst of kidney, acquired | genitourinary | 272 | 1.13 (0.99-1.29) | 0.07 |
| 198.4 | Secondary malignant neoplasm of liver | neoplasms | 272 | 0.92 (0.84-1.01) | 0.07 |
| 706 | Diseases of sebaceous glands | dermatologic | 272 | 0.95 (0.91-1.00) | 0.07 |
| 530.1 | Esophagitis, GERD and related diseases | digestive | 272 | 0.97 (0.94-1.00) | 0.07 |
| 378.1 | Strabismus (not specified as paralytic) | sense organs | 272 | 0.87 (0.74-1.01) | 0.07 |
| 760 | Back pain | symptoms | 272 | 1.05 (1.00-1.10) | 0.07 |
| 694.2 | Other dyschromia | dermatologic | 272 | 1.16 (0.99-1.37) | 0.07 |
| 300 | Anxiety disorders | mental disorders | 272 | 0.95 (0.89-1.01) | 0.07 |
| 414 | Other forms of chronic heart disease | circulatory system | 272 | 0.90 (0.81-1.01) | 0.07 |
| 480 | Pneumonia | respiratory | 272 | 0.96 (0.91-1.00) | 0.08 |
| 728 | Disorders of muscle, ligament, and fascia | musculoskeletal | 272 | 1.07 (0.99-1.14) | 0.08 |
| 303 | Psychogenic and somatoform disorders | mental disorders | 272 | 1.20 (0.98-1.47) | 0.08 |
| 701.2 | Scar conditions and fibrosis of skin | dermatologic | 272 | 1.09 (0.99-1.20) | 0.08 |
| 858 | Complication of internal orthopedic device | injuries & poisonings | 272 | 0.93 (0.85-1.01) | 0.08 |
| 384.4 | Perforation of tympanic membrane | sense organs | 272 | 0.88 (0.76-1.02) | 0.08 |
| 465.2 | Acute pharyngitis | respiratory | 272 | 1.15 (0.98-1.35) | 0.08 |
| 598 | Abnormal findings on examination of urine | genitourinary | 272 | 0.93 (0.86-1.01) | 0.08 |
| 427 | Cardiac dysrhythmias | circulatory system | 272 | 0.97 (0.93-1.00) | 0.08 |
| 380.1 | Otitis externa | sense organs | 272 | 0.84 (0.68-1.03) | 0.09 |
| 260.6 | Anorexia | endocrine/metabolic | 272 | 0.86 (0.72-1.02) | 0.09 |
| 530 | Diseases of esophagus | digestive | 272 | 0.97 (0.94-1.00) | 0.09 |
| 793.2 | Nonspecific abnormal findings on radiological and other examination of other intrathoracic organs (echocardiogram, etc) | circulatory system | 272 | 1.18 (0.97-1.44) | 0.09 |
| 292.4 | Altered mental status | mental disorders | 272 | 0.92 (0.83-1.01) | 0.09 |
| 433 | Cerebrovascular disease | circulatory system | 272 | 0.95 (0.90-1.01) | 0.09 |
| 158 | Neoplasm of unspecified nature of digestive system | neoplasms | 272 | 0.88 (0.77-1.02) | 0.09 |
| 278.1 | Obesity | endocrine/metabolic | 272 | 0.95 (0.89-1.01) | 0.09 |
| 433.3 | Cerebral ischemia | circulatory system | 272 | 0.93 (0.85-1.01) | 0.09 |
| 965.1 | Opiates and related narcotics causing adverse effects in therapeutic use | injuries & poisonings | 272 | 1.12 (0.98-1.29) | 0.09 |
| 540 | Appendiceal conditions | digestive | 272 | 1.07 (0.99-1.16) | 0.10 |
| 198.2 | Secondary malignancy of respiratory organs | neoplasms | 272 | 1.09 (0.99-1.20) | 0.10 |
| 465 | Acute upper respiratory infections of multiple or unspecified sites | respiratory | 272 | 1.08 (0.99-1.19) | 0.10 |
| 702.2 | Seborrheic keratosis | dermatologic | 272 | 1.08 (0.99-1.19) | 0.10 |
| 394.3 | Aortic valve disease | circulatory system | 272 | 0.88 (0.76-1.02) | 0.10 |
| 348 | Other conditions of brain | neurological | 272 | 0.90 (0.80-1.02) | 0.10 |
| 530.9 | Heartburn | digestive | 272 | 0.92 (0.83-1.02) | 0.10 |
| 300.13 | Phobia | mental disorders | 272 | 1.21 (0.96-1.53) | 0.10 |
| 716.9 | Arthropathy NOS | musculoskeletal | 272 | 0.97 (0.93-1.01) | 0.10 |
| 496.1 | Emphysema | respiratory | 272 | 0.91 (0.81-1.02) | 0.10 |
| 250.1 | Type 1 diabetes | endocrine/metabolic | 272 | 0.91 (0.81-1.02) | 0.11 |
| 804 | Fracture of hand or wrist | injuries & poisonings | 272 | 1.07 (0.99-1.16) | 0.11 |
| 716.2 | Unspecified monoarthritis | musculoskeletal | 272 | 0.96 (0.92-1.01) | 0.11 |
| 742 | Derangement of joint, nontraumatic | musculoskeletal | 272 | 0.93 (0.84-1.02) | 0.11 |
| 562.1 | Diverticulosis | digestive | 272 | 0.97 (0.93-1.01) | 0.11 |
| 427.12 | Paroxysmal ventricular tachycardia | circulatory system | 272 | 0.88 (0.76-1.03) | 0.11 |
| 395.6 | Heart valve replaced | circulatory system | 272 | 0.90 (0.78-1.03) | 0.12 |
| 198.1 | Secondary malignancy of lymph nodes | neoplasms | 272 | 1.06 (0.99-1.14) | 0.12 |
| 946 | Anaphylactic shock NOS | injuries & poisonings | 272 | 0.84 (0.68-1.04) | 0.12 |
| 556 | Ulceration of the lower GI tract | digestive | 272 | 0.89 (0.77-1.03) | 0.12 |
| 562 | Diverticulosis and diverticulitis | digestive | 272 | 0.97 (0.93-1.01) | 0.12 |
| 372 | Disorders of conjunctiva | sense organs | 272 | 0.88 (0.74-1.04) | 0.12 |
| 574.12 | Cholelithiasis with other cholecystitis | digestive | 272 | 0.94 (0.87-1.02) | 0.12 |
| 1005 | Other symptoms | symptoms | 272 | 0.90 (0.79-1.03) | 0.13 |
| 252 | Disorders of parathyroid gland | endocrine/metabolic | 272 | 0.88 (0.76-1.04) | 0.13 |
| 550.1 | Inguinal hernia | digestive | 272 | 1.04 (0.99-1.09) | 0.13 |
| 368.2 | Diplopia and disorders of binocular vision | sense organs | 272 | 0.88 (0.74-1.04) | 0.13 |
| 727.4 | Ganglion and cyst of synovium, tendon, and bursa | musculoskeletal | 272 | 0.94 (0.86-1.02) | 0.13 |
| 41 | Bacterial infection NOS | infectious diseases | 272 | 0.97 (0.93-1.01) | 0.13 |
| 78 | Viral warts & HPV | infectious diseases | 272 | 0.89 (0.77-1.04) | 0.13 |
| 374 | Other disorders of eyelids | sense organs | 272 | 1.05 (0.99-1.12) | 0.14 |
| 198.3 | Secondary malignant neoplasm of digestive systems | neoplasms | 272 | 0.91 (0.81-1.03) | 0.14 |
| 458.9 | Hypotension NOS | circulatory system | 272 | 0.94 (0.87-1.02) | 0.14 |
| 386.9 | Dizziness and giddiness (Light-headedness and vertigo) | sense organs | 272 | 0.95 (0.89-1.02) | 0.14 |
| 318 | Tobacco use disorder | mental disorders | 272 | 0.97 (0.94-1.01) | 0.14 |
| 573.5 | Jaundice (not of newborn) | digestive | 272 | 0.88 (0.74-1.04) | 0.14 |
| 915 | Superficial injury without mention of infection | injuries & poisonings | 272 | 0.95 (0.88-1.02) | 0.14 |
| 540.1 | Appendicitis | digestive | 272 | 1.06 (0.98-1.16) | 0.14 |
| 756 | Other congenital musculoskeletal anomalies | congenital anomalies | 272 | 0.87 (0.72-1.05) | 0.15 |
| 859 | Complication due to other implant and internal device | injuries & poisonings | 272 | 0.94 (0.87-1.02) | 0.15 |
| 761 | Cervicalgia | symptoms | 272 | 0.92 (0.83-1.03) | 0.15 |
| 687.4 | Disturbance of skin sensation | dermatologic | 272 | 0.94 (0.86-1.02) | 0.15 |
| 228 | Hemangioma and lymphangioma, any site | neoplasms | 272 | 0.92 (0.82-1.03) | 0.15 |
| 426.31 | Right bundle branch block | circulatory system | 272 | 0.92 (0.81-1.03) | 0.15 |
| 426.24 | Atrioventricular block, complete | circulatory system | 272 | 0.87 (0.72-1.05) | 0.15 |
| 571.5 | Other chronic nonalcoholic liver disease | digestive | 272 | 0.91 (0.81-1.04) | 0.16 |
| 531.1 | Hemorrhage from gastrointestinal ulcer | digestive | 272 | 0.87 (0.72-1.05) | 0.16 |
| 366.2 | Senile cataract | sense organs | 272 | 0.96 (0.91-1.01) | 0.16 |
| 352 | Disorders of other cranial nerves | neurological | 272 | 0.91 (0.81-1.04) | 0.16 |
| 430 | Intracranial hemorrhage | circulatory system | 272 | 0.92 (0.83-1.03) | 0.16 |
| 473.4 | Voice disturbance | respiratory | 272 | 0.90 (0.77-1.04) | 0.16 |
| 735.3 | Hallux valgus (Bunion) | musculoskeletal | 272 | 0.96 (0.90-1.02) | 0.16 |
| 705 | Disorders of sweat glands | dermatologic | 272 | 0.88 (0.73-1.05) | 0.16 |
| 530.12 | Ulcer of esophagus | digestive | 272 | 1.05 (0.98-1.12) | 0.17 |
| 564.9 | Personal history of diseases of digestive system | digestive | 272 | 0.97 (0.94-1.01) | 0.17 |
| 823 | Torus fracture | injuries & poisonings | 272 | 1.08 (0.97-1.22) | 0.17 |
| 1011 | Complications of surgical and medical procedures | injuries & poisonings | 272 | 0.97 (0.92-1.01) | 0.17 |
| 512.9 | Other dyspnea | respiratory | 272 | 1.11 (0.95-1.30) | 0.17 |
| 857 | Mechanical complication of unspecified genitourinary device, implant, and graft | injuries & poisonings | 272 | 1.10 (0.96-1.25) | 0.17 |
| 717 | Polymyalgia Rheumatica | musculoskeletal | 272 | 0.91 (0.79-1.04) | 0.17 |
| 592.1 | Cystitis | genitourinary | 272 | 0.94 (0.86-1.03) | 0.18 |
| 803.2 | Fracture of radius and ulna | injuries & poisonings | 272 | 1.06 (0.98-1.14) | 0.18 |
| 716.1 | Unspecified polyarthropathy or polyarthritis | musculoskeletal | 272 | 1.06 (0.97-1.16) | 0.18 |
| 703 | Diseases of nail, NOS | dermatologic | 272 | 0.91 (0.80-1.04) | 0.18 |
| 229 | Benign neoplasm of unspecified sites | neoplasms | 272 | 1.07 (0.97-1.17) | 0.18 |
| 747.13 | Congenital anomalies of great vessels | congenital anomalies | 272 | 0.93 (0.83-1.04) | 0.18 |
| 420.2 | Pericarditis | circulatory system | 272 | 0.91 (0.80-1.04) | 0.18 |
| 189.21 | Malignant neoplasm of bladder | neoplasms | 272 | 0.93 (0.84-1.03) | 0.18 |
| 286 | Coagulation defects | hematopoietic | 272 | 0.90 (0.77-1.05) | 0.18 |
| 353 | Nerve root and plexus disorders | neurological | 272 | 0.91 (0.79-1.05) | 0.18 |
| 531.2 | Gastric ulcer | digestive | 272 | 1.05 (0.98-1.13) | 0.19 |
| 208 | Benign neoplasm of colon | neoplasms | 272 | 0.97 (0.93-1.01) | 0.19 |
| 703.1 | Ingrowing nail | dermatologic | 272 | 0.90 (0.78-1.05) | 0.19 |
| 240 | Simple and unspecified goiter | endocrine/metabolic | 272 | 1.13 (0.94-1.37) | 0.19 |
| 379.2 | Disorders of vitreous body | sense organs | 272 | 1.09 (0.96-1.23) | 0.19 |
| 961.1 | Poisoning/allergy of sulfonamides | injuries & poisonings | 272 | 1.11 (0.95-1.30) | 0.19 |
| 726.3 | Bursitis | musculoskeletal | 272 | 0.90 (0.77-1.06) | 0.20 |
| 526 | Diseases of the jaws | digestive | 272 | 1.10 (0.95-1.28) | 0.20 |
| 367.1 | Myopia | sense organs | 272 | 1.09 (0.96-1.24) | 0.20 |
| 793 | Nonspecific abnormal findings on radiological and other examination of musculoskeletal system | injuries & poisonings | 272 | 1.12 (0.94-1.34) | 0.20 |
| 374.1 | Ectropion or entropion | sense organs | 272 | 1.10 (0.95-1.27) | 0.20 |
| 716 | Other arthropathies | musculoskeletal | 272 | 0.98 (0.94-1.01) | 0.21 |
| 300.1 | Anxiety disorder | mental disorders | 272 | 0.96 (0.90-1.02) | 0.21 |
| 287 | Purpura and other hemorrhagic conditions | hematopoietic | 272 | 1.07 (0.96-1.20) | 0.21 |
| 366 | Cataract | sense organs | 272 | 0.98 (0.94-1.01) | 0.21 |
| 427.42 | Cardiac arrest | circulatory system | 272 | 0.91 (0.78-1.06) | 0.22 |
| 530.11 | GERD | digestive | 272 | 0.97 (0.93-1.02) | 0.23 |
| 512.8 | Cough | respiratory | 272 | 1.06 (0.97-1.15) | 0.23 |
| 772 | Symptoms of the muscles | symptoms | 272 | 1.11 (0.93-1.33) | 0.23 |
| 280 | Iron deficiency anemias | hematopoietic | 272 | 0.97 (0.92-1.02) | 0.23 |
| 433.8 | Late effects of cerebrovascular disease | circulatory system | 272 | 0.92 (0.81-1.05) | 0.23 |
| 389 | Hearing loss | sense organs | 272 | 0.96 (0.89-1.03) | 0.24 |
| 696.41 | Psoriasis vulgaris | dermatologic | 272 | 0.92 (0.80-1.06) | 0.24 |
| 174 | Breast cancer | neoplasms | 272 | 1.03 (0.98-1.08) | 0.24 |
| 523 | Gingival and periodontal diseases | digestive | 272 | 1.07 (0.96-1.19) | 0.24 |
| 394.7 | Disease of tricuspid valve | circulatory system | 272 | 0.92 (0.80-1.06) | 0.25 |
| 702 | Degenerative skin conditions and other dermatoses | dermatologic | 272 | 1.04 (0.97-1.11) | 0.25 |
| 575.8 | Other disorders of biliary tract | digestive | 272 | 0.92 (0.79-1.06) | 0.25 |
| 727.1 | Synovitis and tenosynovitis | musculoskeletal | 272 | 0.95 (0.86-1.04) | 0.25 |
| 559 | Ileostomy status | digestive | 272 | 1.07 (0.95-1.20) | 0.26 |
| 350.2 | Abnormality of gait | neurological | 272 | 0.93 (0.83-1.05) | 0.26 |
| 277 | Other disorders of metabolism | endocrine/metabolic | 272 | 1.08 (0.94-1.25) | 0.26 |
| 721.1 | Spondylosis without myelopathy | musculoskeletal | 272 | 0.96 (0.89-1.03) | 0.26 |
| 429 | Ill-defined descriptions and complications of heart disease | circulatory system | 272 | 0.91 (0.77-1.07) | 0.26 |
| 427.5 | Arrhythmia (cardiac) NOS | circulatory system | 272 | 1.09 (0.94-1.27) | 0.27 |
| 379 | Other disorders of eye | sense organs | 272 | 1.04 (0.97-1.12) | 0.27 |
| 195 | Cancer, suspected or other | neoplasms | 272 | 0.98 (0.94-1.02) | 0.28 |
| 592 | Cystitis and urethritis | genitourinary | 272 | 0.95 (0.88-1.04) | 0.28 |
| 788 | Syncope and collapse | symptoms | 272 | 0.97 (0.92-1.02) | 0.28 |
| 351 | Other peripheral nerve disorders | neurological | 272 | 0.97 (0.93-1.02) | 0.28 |
| 375.2 | Epiphora | sense organs | 272 | 1.09 (0.93-1.27) | 0.28 |
| 818 | Intracranial hemorrhage (injury) | injuries & poisonings | 272 | 0.89 (0.73-1.10) | 0.28 |
| 740.9 | Osteoarthrosis NOS | musculoskeletal | 272 | 0.97 (0.92-1.02) | 0.29 |
| 805 | Fracture of vertebral column without mention of spinal cord injury | injuries & poisonings | 272 | 0.93 (0.82-1.06) | 0.29 |
| 455 | Hemorrhoids | circulatory system | 272 | 0.98 (0.95-1.02) | 0.29 |
| 317 | Alcohol-related disorders | mental disorders | 272 | 0.98 (0.94-1.02) | 0.30 |
| 189.2 | Cancer of bladder | neoplasms | 272 | 0.95 (0.86-1.05) | 0.30 |
| 695 | Erythematous conditions | dermatologic | 272 | 1.05 (0.96-1.16) | 0.30 |
| 276.13 | Hyperpotassemia | endocrine/metabolic | 272 | 0.92 (0.80-1.07) | 0.30 |
| 476 | Allergic rhinitis | respiratory | 272 | 0.93 (0.81-1.07) | 0.31 |
| 578.8 | Hemorrhage of rectum and anus | digestive | 272 | 0.98 (0.94-1.02) | 0.32 |
| 960.2 | Allergy/adverse effect of penicillin | injuries & poisonings | 272 | 0.98 (0.94-1.02) | 0.32 |
| 687 | Symptoms affecting skin | dermatologic | 272 | 0.97 (0.91-1.03) | 0.32 |
| 736 | Other acquired deformities of limbs | musculoskeletal | 272 | 1.06 (0.94-1.20) | 0.32 |
| 722.6 | Degeneration of intervertebral disc | musculoskeletal | 272 | 0.95 (0.86-1.05) | 0.32 |
| 585.2 | Renal failure NOS | genitourinary | 272 | 0.94 (0.83-1.06) | 0.33 |
| 290.1 | Dementias | mental disorders | 272 | 0.89 (0.71-1.12) | 0.33 |
| 415.2 | Chronic pulmonary heart disease | circulatory system | 272 | 0.91 (0.75-1.10) | 0.33 |
| 189 | Cancer of urinary organs (incl. kidney and bladder) | neoplasms | 272 | 0.96 (0.89-1.04) | 0.33 |
| 801 | Fracture of ankle and foot | injuries & poisonings | 272 | 0.95 (0.87-1.05) | 0.33 |
| 530.14 | Reflux esophagitis | digestive | 272 | 0.97 (0.92-1.03) | 0.34 |
| 342 | Hemiplegia | neurological | 272 | 0.94 (0.84-1.06) | 0.34 |
| 317.11 | Alcoholic liver damage | mental disorders | 272 | 0.92 (0.78-1.09) | 0.34 |
| 365.1 | Open-angle glaucoma | sense organs | 272 | 1.08 (0.92-1.26) | 0.34 |
| 275.5 | Disorders of calcium/phosphorus metabolism | endocrine/metabolic | 272 | 1.07 (0.93-1.24) | 0.34 |
| 365.2 | Primary angle-closure glaucoma | sense organs | 272 | 0.92 (0.77-1.10) | 0.35 |
| 172.2 | Other non-epithelial cancer of skin | neoplasms | 272 | 1.02 (0.97-1.08) | 0.35 |
| 365.11 | Primary open angle glaucoma | sense organs | 272 | 1.08 (0.92-1.27) | 0.35 |
| 361 | Retinal detachments and defects | sense organs | 272 | 0.96 (0.88-1.05) | 0.35 |
| 386.3 | Labyrinthitis | sense organs | 272 | 0.92 (0.78-1.09) | 0.35 |
| 214.1 | Lipoma of skin and subcutaneous tissue | neoplasms | 272 | 0.96 (0.89-1.04) | 0.36 |
| 530.2 | Esophageal bleeding (varices/hemorrhage) | digestive | 272 | 1.06 (0.94-1.18) | 0.36 |
| 705.8 | Hyperhidrosis | dermatologic | 272 | 1.10 (0.89-1.36) | 0.36 |
| 300.12 | Agorophobia, social phobia, and panic disorder | mental disorders | 272 | 1.09 (0.91-1.29) | 0.36 |
| 516 | Abnormal sputum | respiratory | 272 | 0.95 (0.86-1.06) | 0.36 |
| 574.11 | Cholelithiasis with acute cholecystitis | digestive | 272 | 0.94 (0.83-1.07) | 0.36 |
| 579 | Other symptoms involving abdomen and pelvis | digestive | 272 | 1.04 (0.96-1.13) | 0.37 |
| 367 | Disorders of refraction and accommodation; blindness and low vision | sense organs | 272 | 0.96 (0.87-1.05) | 0.37 |
| 195.1 | Malignant neoplasm, other | neoplasms | 272 | 0.98 (0.94-1.02) | 0.37 |
| 766 | Neuralgia, neuritis, and radiculitis NOS | symptoms | 272 | 0.94 (0.82-1.08) | 0.37 |
| 550 | Abdominal hernia | digestive | 272 | 0.99 (0.96-1.01) | 0.37 |
| 281 | Other deficiency anemia | hematopoietic | 272 | 1.07 (0.93-1.23) | 0.37 |
| 281.1 | Megaloblastic anemia | hematopoietic | 272 | 1.07 (0.93-1.23) | 0.37 |
| 261 | Vitamin deficiency | endocrine/metabolic | 272 | 0.94 (0.82-1.08) | 0.37 |
| 512.7 | Shortness of breath | respiratory | 272 | 0.97 (0.91-1.03) | 0.38 |
| 738.4 | Acquired spondylolisthesis | musculoskeletal | 272 | 1.05 (0.94-1.19) | 0.38 |
| 274.21 | Chondrocalcinosis | endocrine/metabolic | 272 | 0.92 (0.75-1.12) | 0.39 |
| 564.1 | Irritable Bowel Syndrome | digestive | 272 | 0.97 (0.91-1.04) | 0.39 |
| 8.6 | Viral Enteritis | infectious diseases | 272 | 0.93 (0.80-1.09) | 0.39 |
| 696 | Psoriasis and related disorders | dermatologic | 272 | 0.95 (0.84-1.07) | 0.39 |
| 701 | Other hypertrophic and atrophic conditions of skin | dermatologic | 272 | 1.03 (0.96-1.10) | 0.40 |
| 507 | Pleurisy; pleural effusion | respiratory | 272 | 0.98 (0.92-1.03) | 0.40 |
| 594 | Urinary calculus | genitourinary | 272 | 1.03 (0.96-1.10) | 0.40 |
| 540.11 | Acute appendicitis | digestive | 272 | 1.04 (0.95-1.14) | 0.40 |
| 696.4 | Psoriasis | dermatologic | 272 | 0.95 (0.84-1.07) | 0.41 |
| 191 | Manlignant and unknown neoplasms of brain and nervous system | neoplasms | 272 | 1.08 (0.90-1.29) | 0.41 |
| 427.3 | Other specified cardiac dysrhythmias | circulatory system | 272 | 0.97 (0.89-1.05) | 0.41 |
| 454.1 | Varicose veins of lower extremity | circulatory system | 272 | 1.02 (0.97-1.08) | 0.41 |
| 599.2 | Retention of urine | genitourinary | 272 | 1.02 (0.97-1.09) | 0.41 |
| 427.1 | Paroxysmal tachycardia, unspecified | circulatory system | 272 | 0.97 (0.89-1.05) | 0.41 |
| 402 | Elevated blood pressure reading without diagnosis of hypertension | circulatory system | 272 | 0.95 (0.83-1.08) | 0.42 |
| 227 | Benign neoplasm of other endocrine glands and related structures | neoplasms | 272 | 1.07 (0.91-1.25) | 0.42 |
| 357 | Inflammatory and toxic neuropathy | neurological | 272 | 0.95 (0.84-1.08) | 0.42 |
| 560.1 | Paralytic ileus | digestive | 272 | 0.92 (0.75-1.13) | 0.43 |
| 415 | Pulmonary heart disease | circulatory system | 272 | 0.97 (0.89-1.05) | 0.43 |
| 578 | Gastrointestinal hemorrhage | digestive | 272 | 0.99 (0.96-1.02) | 0.43 |
| 452 | Other venous embolism and thrombosis | circulatory system | 272 | 0.92 (0.76-1.13) | 0.43 |
| 740.1 | Osteoarthritis; localized | musculoskeletal | 272 | 1.02 (0.98-1.06) | 0.44 |
| 571.81 | Portal hypertension | digestive | 272 | 0.92 (0.75-1.13) | 0.44 |
| 386.1 | Meniere's disease | sense organs | 272 | 1.08 (0.89-1.31) | 0.44 |
| 433.2 | Occlusion of cerebral arteries | circulatory system | 272 | 1.03 (0.96-1.11) | 0.44 |
| 215 | Other benign neoplasm of connective and other soft tissue | neoplasms | 272 | 0.95 (0.82-1.09) | 0.44 |
| 198 | Secondary malignant neoplasm | neoplasms | 272 | 1.02 (0.97-1.07) | 0.44 |
| 681.1 | Cellulitis and abscess of fingers/toes | dermatologic | 272 | 1.08 (0.89-1.31) | 0.44 |
| 870 | Open wounds of head; neck; and trunk | injuries & poisonings | 272 | 1.03 (0.96-1.11) | 0.44 |
| 454 | Varicose veins | circulatory system | 272 | 1.02 (0.97-1.08) | 0.44 |
| 599.3 | Dysuria | genitourinary | 272 | 0.95 (0.83-1.09) | 0.45 |
| 697 | Sarcoidosis | dermatologic | 272 | 0.92 (0.74-1.14) | 0.45 |
| 735.23 | Hallux rigidus | musculoskeletal | 272 | 1.05 (0.93-1.18) | 0.45 |
| 427.2 | Atrial fibrillation and flutter | circulatory system | 272 | 0.98 (0.93-1.03) | 0.45 |
| 743 | Osteoporosis, osteopenia and pathological fracture | musculoskeletal | 272 | 1.02 (0.96-1.09) | 0.45 |
| 501 | Pneumonitis due to inhalation of food or vomitus | respiratory | 272 | 0.93 (0.77-1.13) | 0.45 |
| 751.2 | Congenital anomalies of urinary system | congenital anomalies | 272 | 1.06 (0.91-1.25) | 0.45 |
| 565 | Anal and rectal conditions | digestive | 272 | 0.99 (0.95-1.02) | 0.46 |
| 295 | Schizophrenia and other psychotic disorders | mental disorders | 272 | 1.06 (0.91-1.25) | 0.46 |
| 550.2 | Diaphragmatic hernia | digestive | 272 | 0.99 (0.95-1.02) | 0.46 |
| 379.3 | Aphakia and other disorders of lens | sense organs | 272 | 0.96 (0.86-1.07) | 0.46 |
| 153.2 | Colon cancer | neoplasms | 272 | 1.03 (0.94-1.14) | 0.47 |
| 416 | Cardiomegaly | circulatory system | 272 | 1.03 (0.94-1.13) | 0.47 |
| 253 | Disorders of the pituitary gland and its hypothalamic control | endocrine/metabolic | 272 | 1.07 (0.89-1.27) | 0.47 |
| 151 | Cancer of stomach | neoplasms | 272 | 1.07 (0.88-1.31) | 0.48 |
| 38.1 | Gram negative septicemia | infectious diseases | 272 | 0.94 (0.80-1.11) | 0.48 |
| 280.1 | Iron deficiency anemias, unspecified or not due to blood loss | hematopoietic | 272 | 0.98 (0.93-1.04) | 0.48 |
| 592.12 | Chronic cystitis | genitourinary | 272 | 0.95 (0.81-1.11) | 0.49 |
| 743.11 | Osteoporosis NOS | musculoskeletal | 272 | 1.03 (0.95-1.11) | 0.49 |
| 612.2 | Hypertrophy of breast (Gynecomastia) | genitourinary | 272 | 1.06 (0.90-1.24) | 0.49 |
| 368.9 | Subjective visual disturbances | sense organs | 272 | 0.93 (0.76-1.14) | 0.49 |
| 425 | Cardiomyopathy | circulatory system | 272 | 1.05 (0.92-1.20) | 0.49 |
| 197 | Chemotherapy | neoplasms | 272 | 1.01 (0.98-1.04) | 0.50 |
| 150 | Cancer of esophagus | neoplasms | 272 | 0.94 (0.79-1.12) | 0.50 |
| 550.3 | Femoral hernia | digestive | 272 | 0.94 (0.78-1.13) | 0.50 |
| 590 | Pyelonephritis | genitourinary | 272 | 0.96 (0.84-1.09) | 0.50 |
| 800 | Fracture of lower limb | injuries & poisonings | 272 | 0.98 (0.92-1.04) | 0.50 |
| 427.11 | Paroxysmal supraventricular tachycardia | circulatory system | 272 | 1.03 (0.94-1.14) | 0.50 |
| 733 | Other disorders of bone and cartilage | musculoskeletal | 272 | 0.97 (0.90-1.05) | 0.50 |
| 689 | Disorder of skin and subcutaneous tissue NOS | dermatologic | 272 | 0.98 (0.92-1.04) | 0.51 |
| 568 | Other disorders of peritoneum | digestive | 272 | 1.03 (0.94-1.13) | 0.51 |
| 561 | Symptoms involving digestive system | digestive | 272 | 1.01 (0.98-1.05) | 0.51 |
| 275 | Disorders of mineral metabolism | endocrine/metabolic | 272 | 1.04 (0.93-1.16) | 0.51 |
| 710 | Osteomyelitis, periostitis, and other infections involving bone | musculoskeletal | 272 | 1.06 (0.88-1.28) | 0.52 |
| 733.4 | Aseptic necrosis of bone | musculoskeletal | 272 | 1.07 (0.87-1.30) | 0.52 |
| 497 | Bronchitis | respiratory | 272 | 0.94 (0.78-1.13) | 0.52 |
| 578.2 | Blood in stool | digestive | 272 | 1.03 (0.94-1.13) | 0.52 |
| 721 | Spondylosis and allied disorders | musculoskeletal | 272 | 0.98 (0.93-1.04) | 0.52 |
| 514 | Abnormal findings examination of lungs | respiratory | 272 | 0.97 (0.88-1.06) | 0.52 |
| 287.3 | Thrombocytopenia | hematopoietic | 272 | 1.04 (0.92-1.17) | 0.52 |
| 722.1 | Displacement of intervertebral disc | musculoskeletal | 272 | 0.94 (0.76-1.15) | 0.53 |
| 289.5 | Diseases of spleen | hematopoietic | 272 | 1.07 (0.87-1.31) | 0.53 |
| 344 | Other paralytic syndromes | neurological | 272 | 0.94 (0.79-1.13) | 0.53 |
| 345 | Epilepsy, recurrent seizures, convulsions | neurological | 272 | 0.98 (0.92-1.05) | 0.54 |
| 217 | Vascular hamartomas and non-neoplastic nevi | neoplasms | 272 | 1.06 (0.88-1.27) | 0.54 |
| 599.5 | Frequency of urination and polyuria | genitourinary | 272 | 0.98 (0.91-1.05) | 0.54 |
| 292.3 | Memory loss | mental disorders | 272 | 1.06 (0.89-1.26) | 0.55 |
| 707.1 | Decubitus ulcer | dermatologic | 272 | 1.05 (0.90-1.22) | 0.55 |
| 750 | Digestive congenital anomalies | congenital anomalies | 272 | 1.06 (0.89-1.26) | 0.55 |
| 317.1 | Alcoholism | mental disorders | 272 | 0.98 (0.94-1.04) | 0.55 |
| 535.8 | Other specified gastritis | digestive | 272 | 0.98 (0.93-1.04) | 0.55 |
| 740 | Osteoarthrosis | musculoskeletal | 272 | 0.99 (0.95-1.03) | 0.57 |
| 735 | Acquired foot deformities | musculoskeletal | 272 | 0.99 (0.94-1.03) | 0.57 |
| 836 | Traumatic arthropathy | injuries & poisonings | 272 | 0.94 (0.77-1.15) | 0.57 |
| 686.3 | Pilonidal cyst | dermatologic | 272 | 0.95 (0.78-1.15) | 0.57 |
| 1000 | Burns | injuries & poisonings | 272 | 0.94 (0.76-1.16) | 0.57 |
| 798.1 | Chronic fatigue syndrome | symptoms | 272 | 0.95 (0.78-1.15) | 0.57 |
| 586.4 | Stricture/obstruction of ureter | genitourinary | 272 | 0.96 (0.82-1.12) | 0.58 |
| 835 | Internal derangement of knee | injuries & poisonings | 272 | 0.99 (0.95-1.03) | 0.58 |
| 687.1 | Rash and other nonspecific skin eruption | dermatologic | 272 | 1.03 (0.93-1.14) | 0.59 |
| 295.1 | Schizophrenia | mental disorders | 272 | 1.05 (0.87-1.28) | 0.59 |
| 743.1 | Osteoporosis | musculoskeletal | 272 | 1.02 (0.95-1.09) | 0.59 |
| 613.7 | Other signs and symptoms in breast | genitourinary | 272 | 0.95 (0.80-1.14) | 0.60 |
| 593 | Hematuria | genitourinary | 272 | 0.99 (0.94-1.03) | 0.60 |
| 568.1 | Peritoneal adhesions (postoperative) (postinfection) | digestive | 272 | 1.02 (0.94-1.11) | 0.60 |
| 378 | Strabismus and other disorders of binocular eye movements | sense organs | 272 | 0.97 (0.86-1.09) | 0.60 |
| 475 | Chronic sinusitis | respiratory | 272 | 0.97 (0.87-1.08) | 0.61 |
| 694 | Dyschromia and Vitiligo | dermatologic | 272 | 1.04 (0.90-1.21) | 0.61 |
| 537 | Other disorders of stomach and duodenum | digestive | 272 | 1.02 (0.94-1.11) | 0.61 |
| 276.41 | Acidosis | endocrine/metabolic | 272 | 1.04 (0.90-1.20) | 0.61 |
| 1008 | Crushing or internal injury to organs | injuries & poisonings | 272 | 1.04 (0.90-1.20) | 0.62 |
| 433.31 | Transient cerebral ischemia | circulatory system | 272 | 0.97 (0.88-1.08) | 0.62 |
| 751.1 | Congenital anomalies of genital organs | congenital anomalies | 272 | 0.96 (0.81-1.14) | 0.62 |
| 741.2 | Stiffness of joint | musculoskeletal | 272 | 1.05 (0.87-1.26) | 0.62 |
| 165 | Cancer within the respiratory system | neoplasms | 272 | 0.98 (0.89-1.07) | 0.62 |
| 800.3 | Fracture of tibia and fibula | injuries & poisonings | 272 | 1.03 (0.93-1.13) | 0.62 |
| 577.2 | Chronic pancreatitis | digestive | 272 | 0.95 (0.77-1.17) | 0.63 |
| 41.2 | Streptococcus infection | infectious diseases | 272 | 0.97 (0.87-1.09) | 0.63 |
| 365 | Glaucoma | sense organs | 272 | 1.02 (0.94-1.11) | 0.63 |
| 470 | Septal Deviations/Turbinate Hypertrophy | respiratory | 272 | 1.02 (0.95-1.09) | 0.63 |
| 41.1 | Staphylococcus infections | infectious diseases | 272 | 0.98 (0.90-1.07) | 0.64 |
| 362.4 | Retinal vascular changes and abnomalities | sense organs | 272 | 0.96 (0.82-1.13) | 0.65 |
| 81 | Infection/inflammation of internal prosthetic device; implant; and graft | infectious diseases | 272 | 0.98 (0.89-1.07) | 0.65 |
| 619.1 | Noninflammatory disorders of ovary, fallopian tube, and broad ligament | genitourinary | 272 | 1.04 (0.88-1.24) | 0.66 |
| 480.11 | Pneumococcal pneumonia | respiratory | 272 | 1.01 (0.95-1.08) | 0.66 |
| 563 | Constipation | digestive | 272 | 1.01 (0.96-1.06) | 0.66 |
| 513 | Respiratory abnormalities | respiratory | 272 | 1.04 (0.86-1.26) | 0.67 |
| 751 | Genitourinary congenital anomalies | congenital anomalies | 272 | 1.03 (0.91-1.15) | 0.67 |
| 8.52 | Intestinal infection due to C. difficile | infectious diseases | 272 | 1.04 (0.87-1.25) | 0.67 |
| 726 | Peripheral enthesopathies and allied syndromes | musculoskeletal | 272 | 0.99 (0.95-1.04) | 0.67 |
| 809 | Fracture of unspecified bones | injuries & poisonings | 272 | 1.03 (0.90-1.17) | 0.67 |
| 743.9 | Osteopenia or other disorder of bone and cartilage | musculoskeletal | 272 | 0.97 (0.82-1.14) | 0.68 |
| 202.24 | Large cell lymphoma | neoplasms | 272 | 0.96 (0.79-1.17) | 0.68 |
| 217.1 | Nevus, non-neoplastic | neoplasms | 272 | 1.04 (0.86-1.26) | 0.68 |
| 430.1 | Subarachnoid hemorrhage | circulatory system | 272 | 1.03 (0.88-1.22) | 0.68 |
| 530.5 | Disorders of esophageal motility | digestive | 272 | 0.96 (0.80-1.16) | 0.68 |
| 371.3 | Inflammation of eyelids | sense organs | 272 | 1.02 (0.92-1.13) | 0.68 |
| 274.2 | Crystal arthropathies | endocrine/metabolic | 272 | 0.96 (0.80-1.16) | 0.68 |
| 535.1 | Acute gastritis | digestive | 272 | 1.03 (0.90-1.18) | 0.69 |
| 800.1 | Fracture of neck of femur | injuries & poisonings | 272 | 0.98 (0.87-1.10) | 0.69 |
| 696.42 | Psoriatic arthropathy | dermatologic | 272 | 1.04 (0.87-1.23) | 0.69 |
| 288 | Diseases of white blood cells | hematopoietic | 272 | 1.02 (0.94-1.10) | 0.69 |
| 790.6 | Other abnormal blood chemistry | symptoms | 272 | 0.99 (0.93-1.05) | 0.69 |
| 710.1 | Osteomyelitis | musculoskeletal | 272 | 1.04 (0.86-1.26) | 0.69 |
| 371 | Inflammation of the eye | sense organs | 272 | 0.98 (0.91-1.07) | 0.70 |
| 198.5 | Secondary malignancy of brain/spine | neoplasms | 272 | 0.97 (0.82-1.14) | 0.70 |
| 965 | Poisoning by analgesics, antipyretics, and antirheumatics | injuries & poisonings | 272 | 1.01 (0.96-1.07) | 0.70 |
| 773 | Pain in limb | symptoms | 272 | 1.01 (0.95-1.07) | 0.70 |
| 427.7 | Tachycardia NOS | circulatory system | 272 | 1.02 (0.91-1.15) | 0.70 |
| 425.1 | Primary/intrinsic cardiomyopathies | circulatory system | 272 | 1.03 (0.90-1.17) | 0.71 |
| 871 | Open wounds of extremities | injuries & poisonings | 272 | 0.99 (0.92-1.06) | 0.71 |
| 960 | Poisoning by antibiotics | injuries & poisonings | 272 | 1.01 (0.97-1.05) | 0.71 |
| 289 | Other diseases of blood and blood-forming organs | hematopoietic | 272 | 0.99 (0.92-1.06) | 0.71 |
| 527 | Diseases of the salivary glands | digestive | 272 | 0.97 (0.81-1.15) | 0.72 |
| 586 | Other disorders of the kidney and ureters | genitourinary | 272 | 0.99 (0.91-1.07) | 0.72 |
| 352.2 | Facial nerve disorders [CN7] | neurological | 272 | 0.97 (0.83-1.14) | 0.72 |
| 939 | Atopic/contact dermatitis due to other or unspecified | dermatologic | 272 | 1.02 (0.92-1.13) | 0.72 |
| 594.1 | Calculus of kidney | genitourinary | 272 | 1.02 (0.93-1.11) | 0.72 |
| 364 | Corneal opacity and other disorders of cornea | sense organs | 272 | 1.03 (0.87-1.23) | 0.72 |
| 737.3 | Kyphoscoliosis and scoliosis | musculoskeletal | 272 | 0.97 (0.84-1.12) | 0.72 |
| 454.11 | Varicose veins of lower extremity, symptomtic | circulatory system | 272 | 1.04 (0.84-1.28) | 0.73 |
| 293.1 | Swelling, mass, or lump in head and neck [Space occupying lesion, intracranial NOS] | mental disorders | 272 | 0.97 (0.82-1.15) | 0.73 |
| 535.6 | Duodenitis | digestive | 272 | 0.99 (0.93-1.05) | 0.74 |
| 361.1 | Retinal detachment with retinal defect | sense organs | 272 | 1.02 (0.90-1.16) | 0.74 |
| 741 | Symptoms and disorders of the joints | musculoskeletal | 272 | 1.01 (0.94-1.09) | 0.74 |
| 327.3 | Sleep apnea | neurological | 272 | 0.99 (0.91-1.07) | 0.75 |
| 172 | Skin cancer | neoplasms | 272 | 0.99 (0.95-1.04) | 0.75 |
| 261.2 | Vitamin B-complex deficiencies | endocrine/metabolic | 272 | 0.97 (0.82-1.15) | 0.75 |
| 612 | Breast conditions, congenital or relating to hormones | genitourinary | 272 | 1.02 (0.88-1.19) | 0.75 |
| 790 | Nonspecific findings on examination of blood | symptoms | 272 | 0.99 (0.93-1.05) | 0.75 |
| 681.2 | Cellulitis and abscess of face/neck | dermatologic | 272 | 1.03 (0.84-1.27) | 0.76 |
| 698 | Pruritus and related conditions | dermatologic | 272 | 0.98 (0.83-1.15) | 0.77 |
| 198.6 | Secondary malignancy of bone | neoplasms | 272 | 1.01 (0.92-1.12) | 0.78 |
| 189.11 | Malignant neoplasm of kidney, except pelvis | neoplasms | 272 | 1.02 (0.88-1.18) | 0.78 |
| 369 | Infection of the eye | sense organs | 272 | 0.97 (0.80-1.18) | 0.78 |
| 798 | Malaise and fatigue | symptoms | 272 | 0.99 (0.91-1.07) | 0.79 |
| 916 | Contusion | injuries & poisonings | 272 | 0.98 (0.87-1.11) | 0.79 |
| 200 | Myeloproliferative disease | neoplasms | 272 | 1.02 (0.87-1.20) | 0.79 |
| 714.1 | Rheumatoid arthritis | musculoskeletal | 272 | 1.01 (0.93-1.09) | 0.80 |
| 443.1 | Raynaud's syndrome | circulatory system | 272 | 0.98 (0.86-1.13) | 0.81 |
| 214 | Lipoma | neoplasms | 272 | 0.99 (0.93-1.06) | 0.81 |
| 480.1 | Bacterial pneumonia | respiratory | 272 | 0.99 (0.94-1.05) | 0.82 |
| 293 | Symptoms involving head and neck | mental disorders | 272 | 1.01 (0.92-1.11) | 0.83 |
| 276.4 | Acid-base balance disorder | endocrine/metabolic | 272 | 0.99 (0.86-1.13) | 0.83 |
| 737 | Curvature of spine | musculoskeletal | 272 | 1.01 (0.88-1.17) | 0.84 |
| 522.5 | Periapical abscess | digestive | 272 | 0.99 (0.86-1.13) | 0.84 |
| 389.4 | Tinnitus | sense organs | 272 | 1.02 (0.83-1.25) | 0.85 |
| 450 | Noninfectious disorders of lymphatic channels | circulatory system | 272 | 1.02 (0.85-1.21) | 0.85 |
| 327 | Sleep disorders | neurological | 272 | 1.01 (0.94-1.08) | 0.85 |
| 770 | Myalgia and myositis unspecified | symptoms | 272 | 0.98 (0.82-1.18) | 0.85 |
| 783 | Fever of unknown origin | symptoms | 272 | 0.99 (0.92-1.07) | 0.86 |
| 714 | Rheumatoid arthritis and other inflammatory polyarthropathies | musculoskeletal | 272 | 1.01 (0.93-1.09) | 0.86 |
| 535 | Gastritis and duodenitis | digestive | 272 | 1.00 (0.97-1.03) | 0.87 |
| 567 | Peritonitis and retroperitoneal infections | digestive | 272 | 0.99 (0.84-1.15) | 0.87 |
| 571.8 | Liver abscess and sequelae of chronic liver disease | digestive | 272 | 0.99 (0.84-1.15) | 0.87 |
| 427.6 | Premature beats | circulatory system | 272 | 1.02 (0.83-1.24) | 0.87 |
| 362.29 | Macular degeneration (senile) of retina NOS | sense organs | 272 | 1.01 (0.91-1.11) | 0.88 |
| 362.2 | Degeneration of macula and posterior pole of retina | sense organs | 272 | 1.01 (0.91-1.11) | 0.88 |
| 334 | Degenerative disease of the spinal cord | neurological | 272 | 0.99 (0.89-1.11) | 0.88 |
| 275.1 | Disorders of iron metabolism | hematopoietic | 272 | 0.99 (0.83-1.17) | 0.89 |
| 149 | Cancer of larynx, pharynx, nasal cavities | neoplasms | 272 | 0.99 (0.82-1.19) | 0.89 |
| 1015 | Effects of other external causes | symptoms | 272 | 0.99 (0.93-1.07) | 0.89 |
| 579.8 | Nonspecific abnormal findings in stool contents | digestive | 272 | 1.01 (0.89-1.14) | 0.89 |
| 396 | Abnormal heart sounds | circulatory system | 272 | 1.01 (0.87-1.17) | 0.89 |
| 555.21 | Ulcerative colitis (chronic) | digestive | 272 | 0.99 (0.81-1.21) | 0.89 |
| 191.1 | Cancer of brain and nervous system | neoplasms | 272 | 0.99 (0.81-1.21) | 0.90 |
| 747.11 | Cardiac shunt/ heart septal defect | congenital anomalies | 272 | 1.01 (0.84-1.23) | 0.90 |
| 715.2 | Ankylosing spondylitis | musculoskeletal | 272 | 0.99 (0.82-1.19) | 0.90 |
| 250.4 | Abnormal glucose | endocrine/metabolic | 272 | 1.01 (0.85-1.21) | 0.90 |
| 907 | Injuries to the nervous system | injuries & poisonings | 272 | 0.99 (0.87-1.13) | 0.91 |
| 427.9 | Palpitations | circulatory system | 272 | 1.00 (0.92-1.07) | 0.92 |
| 165.1 | Cancer of bronchus; lung | neoplasms | 272 | 0.99 (0.90-1.10) | 0.92 |
| 870.3 | Other open wound of head and face | injuries & poisonings | 272 | 1.00 (0.92-1.10) | 0.92 |
| 803.3 | Fracture of clavicle or scapula | injuries & poisonings | 272 | 0.99 (0.88-1.12) | 0.92 |
| 512 | Other symptoms of respiratory system | respiratory | 272 | 1.00 (0.95-1.05) | 0.93 |
| 528.5 | Diseases of lips | digestive | 272 | 1.01 (0.84-1.20) | 0.93 |
| 800.4 | Fracture of patella | injuries & poisonings | 272 | 1.01 (0.85-1.19) | 0.93 |
| 210 | Benign neoplasm of lip, oral cavity, and pharynx | neoplasms | 272 | 0.99 (0.86-1.15) | 0.93 |
| 853 | Complication of colostomy or enterostomy | injuries & poisonings | 272 | 0.99 (0.81-1.22) | 0.93 |
| 523.32 | Chronic periodontitis | digestive | 272 | 1.01 (0.83-1.23) | 0.93 |
| 145 | Cancer of mouth | neoplasms | 272 | 0.99 (0.83-1.19) | 0.93 |
| 715 | Other inflammatory spondylopathies | musculoskeletal | 272 | 1.00 (0.90-1.13) | 0.93 |
| 735.2 | Acquired toe deformities | musculoskeletal | 272 | 1.00 (0.94-1.07) | 0.94 |
| 433.21 | Cerebral artery occlusion, with cerebral infarction | circulatory system | 272 | 1.00 (0.89-1.13) | 0.94 |
| 561.2 | Flatulence | digestive | 272 | 1.00 (0.89-1.12) | 0.94 |
| 446 | Polyarteritis nodosa and allied conditions | circulatory system | 272 | 1.01 (0.86-1.18) | 0.94 |
| 189.1 | Cancer of kidney and renal pelvis | neoplasms | 272 | 1.01 (0.87-1.16) | 0.94 |
| 557 | Intestinal malabsorption (non-celiac) | digestive | 272 | 1.01 (0.83-1.22) | 0.95 |
| 743.13 | Other specified osteoporosis | musculoskeletal | 272 | 0.99 (0.81-1.22) | 0.95 |
| 41.4 | E. coli | infectious diseases | 272 | 1.00 (0.91-1.10) | 0.96 |
| 702.1 | Actinic keratosis | dermatologic | 272 | 1.00 (0.90-1.12) | 0.96 |
| 522 | Diseases of pulp and periapical tissues | digestive | 272 | 1.00 (0.90-1.12) | 0.96 |
| 70 | Viral hepatitis | infectious diseases | 272 | 1.00 (0.87-1.14) | 0.97 |
| 729 | Other disorders of soft tissues | musculoskeletal | 272 | 1.00 (0.94-1.07) | 0.97 |
| 199 | Neoplasm of uncertain behavior | neoplasms | 272 | 1.00 (0.87-1.14) | 0.98 |
| 967 | Adverse effects of sedatives or other central nervous system depressants and anesthetics | injuries & poisonings | 272 | 1.00 (0.83-1.21) | 0.99 |
| 375 | Disorders of lacrimal system | sense organs | 272 | 1.00 (0.91-1.11) | 0.99 |
| 288.1 | Decreased white blood cell count | hematopoietic | 272 | 1.00 (0.92-1.09) | 0.99 |
| 288.11 | Neutropenia | hematopoietic | 272 | 1.00 (0.92-1.09) | 0.99 |
| 726.1 | Enthesopathy | musculoskeletal | 272 | 1.00 (0.95-1.05) | 0.99 |
| 966 | Poisoning by anticonvulsants and anti-Parkinsonism drugs | injuries & poisonings | 272 | 1.00 (0.81-1.23) | 1.00 |
| 740.11 | Osteoarthrosis, localized, primary | musculoskeletal | 272 | 1.00 (0.95-1.05) | 1.00 |

Odds ratios (ORs) with their 95% confidence intervals (CIs) represent the association estimates with the risks of non-breast cancer of per 1-SD increase of HDL-C, respectively.

Significant threshold in stage 2 was set at set at *P*<0.05/1358=3.68×10^-5^, which was corrected for multiple comparisons using the Bonferroni method (0.05/1358 [2 identified breast cancer metabolites in stage 1×679 diseases]).

Abbreviations: Phe-MR, phenome-wide Mendelian randomization; SNPs, single nucleotide polymorphisms.

**Table S7. Phe-MR analysis for the associations between acetate and 679 diseases using the inverse-variance weighted method.**

| **PheCode** | **Phenotype Description** | **Disease Category** | **SNPs** | **OR (95% CI)** | ***P* value** |
| --- | --- | --- | --- | --- | --- |
| 477 | Epistaxis or throat hemorrhage | respiratory | 16 | 0.24 (0.15-0.37) | 3.84×10^-10^ |
| 318 | Tobacco use disorder | mental disorders | 16 | 0.61 (0.51-0.72) | 6.87×10^-9^ |
| 394.3 | Aortic valve disease | circulatory system | 16 | 0.19 (0.10-0.36) | 3.13×10^-7^ |
| 433 | Cerebrovascular disease | circulatory system | 16 | 0.54 (0.42-0.69) | 8.46×10^-7^ |
| 365 | Glaucoma | sense organs | 16 | 2.29 (1.63-3.22) | 1.82×10^-6^ |
| 411.1 | Unstable angina (intermediate coronary syndrome) | circulatory system | 16 | 0.46 (0.34-0.64) | 2.22×10^-6^ |
| 854 | Complications of cardiac/vascular device, implant, and graft | injuries & poisonings | 16 | 0.28 (0.17-0.48) | 2.60×10^-6^ |
| 960 | Poisoning by antibiotics | injuries & poisonings | 16 | 0.68 (0.57-0.80) | 7.83×10^-6^ |
| 411.8 | Other chronic ischemic heart disease, unspecified | circulatory system | 16 | 0.55 (0.42-0.72) | 1.01×10^-5^ |
| 960.2 | Allergy/adverse effect of penicillin | injuries & poisonings | 16 | 0.67 (0.55-0.80) | 1.37×10^-5^ |
| 480 | Pneumonia | respiratory | 16 | 0.62 (0.49-0.78) | 3.38×10^-5^ |
| 735.3 | Hallux valgus (Bunion) | musculoskeletal | 16 | 1.80 (1.36-2.39) | 3.77×10^-5^ |
| 365.2 | Primary angle-closure glaucoma | sense organs | 16 | 5.58 (2.40-12.96) | 6.46×10^-5^ |
| 411 | Ischemic Heart Disease | circulatory system | 16 | 0.58 (0.45-0.76) | 8.25×10^-5^ |
| 442 | Other aneurysm | circulatory system | 16 | 0.35 (0.21-0.59) | 9.48×10^-5^ |
| 442.1 | Aortic aneurysm | circulatory system | 16 | 0.32 (0.17-0.58) | 1.78×10^-4^ |
| 585 | Renal failure | genitourinary | 16 | 0.60 (0.46-0.78) | 1.82×10^-4^ |
| 585.1 | Acute renal failure | genitourinary | 16 | 0.53 (0.38-0.74) | 1.84×10^-4^ |
| 427.4 | Cardiac arrest and ventricular fibrillation | circulatory system | 16 | 0.28 (0.15-0.55) | 1.96×10^-4^ |
| 342 | Hemiplegia | neurological | 16 | 0.34 (0.19-0.61) | 2.58×10^-4^ |
| 306 | Other mental disorder | mental disorders | 16 | 0.77 (0.67-0.89) | 3.10×10^-4^ |
| 447 | Other disorders of arteries and arterioles | circulatory system | 16 | 0.32 (0.18-0.60) | 3.11×10^-4^ |
| 480.11 | Pneumococcal pneumonia | respiratory | 16 | 0.58 (0.43-0.78) | 3.25×10^-4^ |
| 411.3 | Angina pectoris | circulatory system | 16 | 0.59 (0.44-0.79) | 3.93×10^-4^ |
| 760 | Back pain | symptoms | 16 | 0.68 (0.55-0.84) | 4.16×10^-4^ |
| 433.8 | Late effects of cerebrovascular disease | circulatory system | 16 | 0.32 (0.17-0.60) | 4.18×10^-4^ |
| 433.1 | Occlusion and stenosis of precerebral arteries | circulatory system | 16 | 0.32 (0.17-0.61) | 5.11×10^-4^ |
| 509 | Respiratory failure, insufficiency, arrest | respiratory | 16 | 0.46 (0.30-0.72) | 6.20×10^-4^ |
| 740 | Osteoarthrosis | musculoskeletal | 16 | 1.28 (1.11-1.48) | 6.28×10^-4^ |
| 550.3 | Femoral hernia | digestive | 16 | 0.14 (0.05-0.44) | 7.55×10^-4^ |
| 429.2 | Abnormal function study of cardiovascular system | circulatory system | 16 | 0.19 (0.07-0.51) | 9.67×10^-4^ |
| 1015 | Effects of other external causes | symptoms | 16 | 0.56 (0.40-0.79) | 1.06×10^-3^ |
| 1002 | Symptoms concerning nutrition, metabolism, and development | symptoms | 16 | 0.60 (0.44-0.82) | 1.10×10^-3^ |
| 735 | Acquired foot deformities | musculoskeletal | 16 | 1.47 (1.16-1.85) | 1.19×10^-3^ |
| 597 | Other disorders of urethra and urinary tract | genitourinary | 16 | 0.56 (0.39-0.79) | 1.21×10^-3^ |
| 198 | Secondary malignant neoplasm | neoplasms | 16 | 1.47 (1.16-1.85) | 1.34×10^-3^ |
| 433.2 | Occlusion of cerebral arteries | circulatory system | 16 | 0.57 (0.40-0.80) | 1.50×10^-3^ |
| 535.8 | Other specified gastritis | digestive | 16 | 1.50 (1.16-1.93) | 1.68×10^-3^ |
| 433.31 | Transient cerebral ischemia | circulatory system | 16 | 0.46 (0.28-0.75) | 1.75×10^-3^ |
| 333 | Extrapyramidal disease and abnormal movement disorders | neurological | 16 | 0.30 (0.14-0.64) | 1.78×10^-3^ |
| 145 | Cancer of mouth | neoplasms | 16 | 0.25 (0.10-0.60) | 1.84×10^-3^ |
| 709.7 | Unspecified diffuse connective tissue disease | dermatologic | 16 | 1.98 (1.29-3.04) | 1.92×10^-3^ |
| 459 | Other disorders of circulatory system | circulatory system | 16 | 0.62 (0.46-0.84) | 1.95×10^-3^ |
| 459.9 | Circulatory disease NEC | circulatory system | 16 | 0.63 (0.47-0.85) | 2.18×10^-3^ |
| 458.9 | Hypotension NOS | circulatory system | 16 | 0.56 (0.38-0.82) | 2.64×10^-3^ |
| 418 | Nonspecific chest pain | circulatory system | 16 | 0.81 (0.71-0.93) | 2.75×10^-3^ |
| 429 | Ill-defined descriptions and complications of heart disease | circulatory system | 16 | 0.30 (0.14-0.66) | 2.84×10^-3^ |
| 317.11 | Alcoholic liver damage | mental disorders | 16 | 0.30 (0.14-0.67) | 3.03×10^-3^ |
| 733.8 | Malunion and nonunion of fracture | musculoskeletal | 16 | 0.39 (0.21-0.73) | 3.16×10^-3^ |
| 411.4 | Coronary atherosclerosis | circulatory system | 16 | 0.61 (0.44-0.85) | 3.18×10^-3^ |
| 473 | Diseases of the larynx and vocal cords | respiratory | 16 | 0.52 (0.34-0.81) | 3.52×10^-3^ |
| 433.3 | Cerebral ischemia | circulatory system | 16 | 0.54 (0.36-0.82) | 3.57×10^-3^ |
| 427 | Cardiac dysrhythmias | circulatory system | 16 | 0.80 (0.69-0.93) | 4.02×10^-3^ |
| 197 | Chemotherapy | neoplasms | 16 | 1.25 (1.07-1.46) | 4.02×10^-3^ |
| 340 | Migraine | neurological | 16 | 0.54 (0.35-0.82) | 4.17×10^-3^ |
| 480.1 | Bacterial pneumonia | respiratory | 16 | 0.67 (0.51-0.88) | 4.43×10^-3^ |
| 443.9 | Peripheral vascular disease, unspecified | circulatory system | 16 | 0.52 (0.34-0.82) | 4.47×10^-3^ |
| 709 | Diffuse diseases of connective tissue | dermatologic | 16 | 1.74 (1.19-2.55) | 4.58×10^-3^ |
| 800.4 | Fracture of patella | injuries & poisonings | 16 | 3.21 (1.43-7.19) | 4.62×10^-3^ |
| 509.2 | Respiratory insufficiency | respiratory | 16 | 0.46 (0.27-0.79) | 4.71×10^-3^ |
| 835 | Internal derangement of knee | injuries & poisonings | 16 | 1.31 (1.09-1.58) | 4.72×10^-3^ |
| 427.42 | Cardiac arrest | circulatory system | 16 | 0.35 (0.17-0.73) | 4.86×10^-3^ |
| 411.2 | Myocardial infarction | circulatory system | 16 | 0.55 (0.36-0.84) | 4.97×10^-3^ |
| 198.1 | Secondary malignancy of lymph nodes | neoplasms | 16 | 1.80 (1.19-2.72) | 5.13×10^-3^ |
| 189.21 | Malignant neoplasm of bladder | neoplasms | 16 | 0.50 (0.31-0.81) | 5.36×10^-3^ |
| 870.3 | Other open wound of head and face | injuries & poisonings | 16 | 0.55 (0.37-0.84) | 5.39×10^-3^ |
| 428 | Congestive heart failure; nonhypertensive | circulatory system | 16 | 0.65 (0.47-0.88) | 5.49×10^-3^ |
| 516 | Abnormal sputum | respiratory | 16 | 0.51 (0.32-0.82) | 5.83×10^-3^ |
| 578.2 | Blood in stool | digestive | 16 | 0.54 (0.35-0.84) | 5.86×10^-3^ |
| 428.2 | Heart failure NOS | circulatory system | 16 | 0.61 (0.43-0.87) | 5.93×10^-3^ |
| 380.1 | Otitis externa | sense organs | 16 | 0.25 (0.10-0.68) | 5.96×10^-3^ |
| 967 | Adverse effects of sedatives or other central nervous system depressants and anesthetics | injuries & poisonings | 16 | 0.28 (0.11-0.69) | 6.09×10^-3^ |
| 772 | Symptoms of the muscles | symptoms | 16 | 3.26 (1.40-7.60) | 6.11×10^-3^ |
| 512.7 | Shortness of breath | respiratory | 16 | 0.56 (0.37-0.85) | 6.30×10^-3^ |
| 519.8 | Other diseases of respiratory system, NEC | respiratory | 16 | 0.72 (0.57-0.91) | 6.75×10^-3^ |
| 519 | Other diseases of respiratory system, not elsewhere classified | respiratory | 16 | 0.73 (0.58-0.92) | 7.13×10^-3^ |
| 433.21 | Cerebral artery occlusion, with cerebral infarction | circulatory system | 16 | 0.45 (0.25-0.81) | 7.36×10^-3^ |
| 597.1 | Urethral stricture (not specified as infectious) | genitourinary | 16 | 0.59 (0.40-0.87) | 7.47×10^-3^ |
| 702.1 | Actinic keratosis | dermatologic | 16 | 1.81 (1.17-2.82) | 8.18×10^-3^ |
| 380 | Disorders of external ear | sense organs | 16 | 0.45 (0.25-0.82) | 8.25×10^-3^ |
| 458 | Hypotension | circulatory system | 16 | 0.67 (0.50-0.90) | 8.40×10^-3^ |
| 721.1 | Spondylosis without myelopathy | musculoskeletal | 16 | 1.53 (1.11-2.11) | 8.57×10^-3^ |
| 426 | Cardiac conduction disorders | circulatory system | 16 | 0.69 (0.53-0.91) | 8.61×10^-3^ |
| 496.2 | Chronic bronchitis | respiratory | 16 | 0.57 (0.38-0.87) | 8.72×10^-3^ |
| 512 | Other symptoms of respiratory system | respiratory | 16 | 0.73 (0.57-0.92) | 8.93×10^-3^ |
| 613.7 | Other signs and symptoms in breast | genitourinary | 16 | 3.07 (1.32-7.14) | 8.99×10^-3^ |
| 368.2 | Diplopia and disorders of binocular vision | sense organs | 16 | 2.98 (1.30-6.82) | 9.67×10^-3^ |
| 372 | Disorders of conjunctiva | sense organs | 16 | 2.91 (1.29-6.53) | 9.77×10^-3^ |
| 427.1 | Paroxysmal tachycardia, unspecified | circulatory system | 16 | 0.59 (0.40-0.88) | 9.94×10^-3^ |
| 790.6 | Other abnormal blood chemistry | symptoms | 16 | 0.69 (0.52-0.92) | 0.01 |
| 375.2 | Epiphora | sense organs | 16 | 2.62 (1.24-5.50) | 0.01 |
| 352.2 | Facial nerve disorders [CN7] | neurological | 16 | 0.38 (0.18-0.80) | 0.01 |
| 870 | Open wounds of head; neck; and trunk | injuries & poisonings | 16 | 0.63 (0.44-0.90) | 0.01 |
| 550.1 | Inguinal hernia | digestive | 16 | 0.79 (0.65-0.95) | 0.01 |
| 495 | Asthma | respiratory | 16 | 0.83 (0.71-0.96) | 0.01 |
| 272.1 | Hyperlipidemia | endocrine/metabolic | 16 | 0.69 (0.51-0.92) | 0.01 |
| 803.1 | Fracture of humerus | injuries & poisonings | 16 | 0.50 (0.29-0.86) | 0.01 |
| 272 | Disorders of lipid metabolism | endocrine/metabolic | 16 | 0.69 (0.51-0.92) | 0.01 |
| 705 | Disorders of sweat glands | dermatologic | 16 | 0.37 (0.17-0.81) | 0.01 |
| 681 | Superficial cellulitis and abscess | dermatologic | 16 | 0.72 (0.55-0.93) | 0.01 |
| 798 | Malaise and fatigue | symptoms | 16 | 0.62 (0.42-0.91) | 0.01 |
| 375 | Disorders of lacrimal system | sense organs | 16 | 1.82 (1.13-2.93) | 0.01 |
| 345 | Epilepsy, recurrent seizures, convulsions | neurological | 16 | 0.60 (0.39-0.91) | 0.02 |
| 697 | Sarcoidosis | dermatologic | 16 | 0.31 (0.12-0.80) | 0.02 |
| 686.1 | Carbuncle and furuncle | dermatologic | 16 | 0.56 (0.35-0.90) | 0.02 |
| 612 | Breast conditions, congenital or relating to hormones | genitourinary | 16 | 0.41 (0.20-0.84) | 0.02 |
| 496 | Chronic airway obstruction | respiratory | 16 | 0.67 (0.48-0.93) | 0.02 |
| 562 | Diverticulosis and diverticulitis | digestive | 16 | 0.84 (0.72-0.97) | 0.02 |
| 473.4 | Voice disturbance | respiratory | 16 | 0.44 (0.22-0.87) | 0.02 |
| 189.2 | Cancer of bladder | neoplasms | 16 | 0.58 (0.37-0.92) | 0.02 |
| 442.11 | Abdominal aortic aneurysm | circulatory system | 16 | 0.41 (0.19-0.87) | 0.02 |
| 172 | Skin cancer | neoplasms | 16 | 1.26 (1.04-1.54) | 0.02 |
| 250.4 | Abnormal glucose | endocrine/metabolic | 16 | 0.36 (0.16-0.85) | 0.02 |
| 454.11 | Varicose veins of lower extremity, symptomtic | circulatory system | 16 | 2.85 (1.17-6.94) | 0.02 |
| 272.11 | Hypercholesterolemia | endocrine/metabolic | 16 | 0.70 (0.52-0.95) | 0.02 |
| 562.1 | Diverticulosis | digestive | 16 | 0.84 (0.73-0.98) | 0.02 |
| 228 | Hemangioma and lymphangioma, any site | neoplasms | 16 | 1.92 (1.10-3.36) | 0.02 |
| 474.1 | Acute tonsillitis | respiratory | 16 | 0.35 (0.14-0.86) | 0.02 |
| 702 | Degenerative skin conditions and other dermatoses | dermatologic | 16 | 1.42 (1.05-1.93) | 0.02 |
| 790 | Nonspecific findings on examination of blood | symptoms | 16 | 0.72 (0.55-0.96) | 0.02 |
| 704 | Diseases of hair and hair follicles | dermatologic | 16 | 0.70 (0.51-0.95) | 0.02 |
| 1010 | Other tests | symptoms | 16 | 1.40 (1.05-1.88) | 0.02 |
| 1001 | Foreign body injury | injuries & poisonings | 16 | 2.09 (1.10-3.98) | 0.02 |
| 274 | Gout and other crystal arthropathies | endocrine/metabolic | 16 | 0.48 (0.25-0.92) | 0.03 |
| 401.2 | Hypertensive heart and/or renal disease | circulatory system | 16 | 0.54 (0.32-0.93) | 0.03 |
| 793 | Nonspecific abnormal findings on radiological and other examination of musculoskeletal system | injuries & poisonings | 16 | 0.39 (0.17-0.90) | 0.03 |
| 990 | Effects radiation NOS | injuries & poisonings | 16 | 0.64 (0.43-0.95) | 0.03 |
| 374.1 | Ectropion or entropion | sense organs | 16 | 2.16 (1.09-4.29) | 0.03 |
| 38 | Septicemia | infectious diseases | 16 | 0.67 (0.47-0.96) | 0.03 |
| 447.1 | Stricture of artery | circulatory system | 16 | 0.43 (0.20-0.92) | 0.03 |
| 592.1 | Cystitis | genitourinary | 16 | 1.58 (1.05-2.40) | 0.03 |
| 567 | Peritonitis and retroperitoneal infections | digestive | 16 | 0.44 (0.21-0.92) | 0.03 |
| 571.8 | Liver abscess and sequelae of chronic liver disease | digestive | 16 | 0.44 (0.21-0.92) | 0.03 |
| 427.6 | Premature beats | circulatory system | 16 | 0.35 (0.13-0.91) | 0.03 |
| 961.1 | Poisoning/allergy of sulfonamides | injuries & poisonings | 16 | 0.44 (0.20-0.93) | 0.03 |
| 440.2 | Atherosclerosis of the extremities | circulatory system | 16 | 0.42 (0.19-0.93) | 0.03 |
| 612.2 | Hypertrophy of breast (Gynecomastia) | genitourinary | 16 | 0.43 (0.20-0.93) | 0.03 |
| 427.3 | Other specified cardiac dysrhythmias | circulatory system | 16 | 0.65 (0.44-0.97) | 0.03 |
| 740.1 | Osteoarthritis; localized | musculoskeletal | 16 | 1.21 (1.02-1.44) | 0.03 |
| 496.21 | Obstructive chronic bronchitis | respiratory | 16 | 0.62 (0.40-0.96) | 0.03 |
| 507 | Pleurisy; pleural effusion | respiratory | 16 | 0.68 (0.47-0.97) | 0.03 |
| 293 | Symptoms involving head and neck | mental disorders | 16 | 1.55 (1.03-2.34) | 0.03 |
| 172.1 | Melanomas of skin, dx or hx | neoplasms | 16 | 1.60 (1.03-2.46) | 0.03 |
| 172.11 | Melanomas of skin | neoplasms | 16 | 1.60 (1.03-2.46) | 0.03 |
| 575.2 | Obstruction of bile duct | digestive | 16 | 0.42 (0.19-0.94) | 0.04 |
| 574.3 | Cholecystitis without cholelithiasis | digestive | 16 | 0.63 (0.41-0.97) | 0.04 |
| 427.12 | Paroxysmal ventricular tachycardia | circulatory system | 16 | 0.46 (0.22-0.96) | 0.04 |
| 523.3 | Periodontitis (acute or chronic) | digestive | 16 | 1.97 (1.04-3.73) | 0.04 |
| 276.14 | Hypopotassemia | endocrine/metabolic | 16 | 0.53 (0.30-0.96) | 0.04 |
| 496.1 | Emphysema | respiratory | 16 | 0.57 (0.33-0.97) | 0.04 |
| 555.2 | Ulcerative colitis | digestive | 16 | 1.94 (1.03-3.67) | 0.04 |
| 857 | Mechanical complication of unspecified genitourinary device, implant, and graft | injuries & poisonings | 16 | 0.51 (0.27-0.97) | 0.04 |
| 747.13 | Congenital anomalies of great vessels | congenital anomalies | 16 | 0.58 (0.34-0.98) | 0.04 |
| 261 | Vitamin deficiency | endocrine/metabolic | 16 | 0.51 (0.27-0.98) | 0.04 |
| 401.22 | Hypertensive chronic kidney disease | circulatory system | 16 | 0.55 (0.31-0.98) | 0.04 |
| 441 | Vascular insufficiency of intestine | circulatory system | 16 | 0.38 (0.15-0.97) | 0.04 |
| 362 | Other retinal disorders | sense organs | 16 | 0.69 (0.48-0.99) | 0.04 |
| 244.1 | Secondary hypothyroidism | endocrine/metabolic | 16 | 1.98 (1.01-3.88) | 0.05 |
| 961 | Poisoning by other antiinfectives | injuries & poisonings | 16 | 0.52 (0.28-0.99) | 0.05 |
| 721 | Spondylosis and allied disorders | musculoskeletal | 16 | 1.30 (1.01-1.68) | 0.05 |
| 532 | Dysphagia | digestive | 16 | 0.75 (0.57-1.00) | 0.05 |
| 565.1 | Anal and rectal polyp | digestive | 16 | 1.30 (1.00-1.70) | 0.05 |
| 522.5 | Periapical abscess | digestive | 16 | 0.52 (0.27-0.99) | 0.05 |
| 585.3 | Chronic renal failure [CKD] | genitourinary | 16 | 0.64 (0.42-1.00) | 0.05 |
| 394 | Rheumatic disease of the heart valves | circulatory system | 16 | 0.72 (0.52-1.00) | 0.05 |
| 735.2 | Acquired toe deformities | musculoskeletal | 16 | 1.37 (1.00-1.89) | 0.05 |
| 296.2 | Depression | mental disorders | 16 | 0.73 (0.53-1.00) | 0.05 |
| 750 | Digestive congenital anomalies | congenital anomalies | 16 | 2.32 (1.00-5.38) | 0.05 |
| 530.14 | Reflux esophagitis | digestive | 16 | 0.80 (0.64-1.00) | 0.05 |
| 426.31 | Right bundle branch block | circulatory system | 16 | 0.56 (0.31-1.00) | 0.05 |
| 696 | Psoriasis and related disorders | dermatologic | 16 | 0.53 (0.28-1.00) | 0.05 |
| 440 | Atherosclerosis | circulatory system | 16 | 0.45 (0.20-1.00) | 0.05 |
| 535 | Gastritis and duodenitis | digestive | 16 | 1.15 (1.00-1.32) | 0.05 |
| 476 | Allergic rhinitis | respiratory | 16 | 0.51 (0.26-1.01) | 0.05 |
| 564.9 | Personal history of diseases of digestive system | digestive | 16 | 1.29 (0.99-1.66) | 0.05 |
| 366 | Cataract | sense organs | 16 | 0.81 (0.65-1.00) | 0.06 |
| 362.2 | Degeneration of macula and posterior pole of retina | sense organs | 16 | 0.55 (0.29-1.01) | 0.06 |
| 530 | Diseases of esophagus | digestive | 16 | 0.88 (0.78-1.00) | 0.06 |
| 696.4 | Psoriasis | dermatologic | 16 | 0.53 (0.27-1.02) | 0.06 |
| 287.3 | Thrombocytopenia | hematopoietic | 16 | 0.58 (0.33-1.02) | 0.06 |
| 362.29 | Macular degeneration (senile) of retina NOS | sense organs | 16 | 0.54 (0.29-1.02) | 0.06 |
| 274.1 | Gout | endocrine/metabolic | 16 | 0.48 (0.22-1.02) | 0.06 |
| 965 | Poisoning by analgesics, antipyretics, and antirheumatics | injuries & poisonings | 16 | 0.77 (0.58-1.01) | 0.06 |
| 287 | Purpura and other hemorrhagic conditions | hematopoietic | 16 | 0.60 (0.35-1.02) | 0.06 |
| 681.6 | Cellulitis and abscess of foot, toe | dermatologic | 16 | 0.75 (0.55-1.01) | 0.06 |
| 225 | Benign neoplasm of brain and other parts of nervous system | neoplasms | 16 | 0.47 (0.22-1.03) | 0.06 |
| 150 | Cancer of esophagus | neoplasms | 16 | 2.22 (0.96-5.11) | 0.06 |
| 859 | Complication due to other implant and internal device | injuries & poisonings | 16 | 1.46 (0.98-2.16) | 0.06 |
| 331 | Other cerebral degenerations | neurological | 16 | 0.53 (0.27-1.04) | 0.06 |
| 426.24 | Atrioventricular block, complete | circulatory system | 16 | 2.40 (0.95-6.04) | 0.06 |
| 395.6 | Heart valve replaced | circulatory system | 16 | 0.44 (0.19-1.05) | 0.06 |
| 1000 | Burns | injuries & poisonings | 16 | 2.39 (0.95-6.05) | 0.07 |
| 300.13 | Phobia | mental disorders | 16 | 0.39 (0.15-1.06) | 0.07 |
| 296 | Mood disorders | mental disorders | 16 | 0.75 (0.55-1.02) | 0.07 |
| 737 | Curvature of spine | musculoskeletal | 16 | 1.87 (0.96-3.63) | 0.07 |
| 454 | Varicose veins | circulatory system | 16 | 0.82 (0.66-1.01) | 0.07 |
| 740.11 | Osteoarthrosis, localized, primary | musculoskeletal | 16 | 1.25 (0.98-1.59) | 0.07 |
| 751.2 | Congenital anomalies of urinary system | congenital anomalies | 16 | 0.49 (0.23-1.05) | 0.07 |
| 216 | Benign neoplasm of skin | neoplasms | 16 | 1.27 (0.98-1.64) | 0.07 |
| 705.8 | Hyperhidrosis | dermatologic | 16 | 0.43 (0.17-1.08) | 0.07 |
| 426.21 | First degree AV block | circulatory system | 16 | 0.52 (0.26-1.07) | 0.07 |
| 720 | Spinal stenosis | musculoskeletal | 16 | 0.71 (0.49-1.03) | 0.07 |
| 426.3 | Bundle branch block | circulatory system | 16 | 0.70 (0.48-1.04) | 0.07 |
| 696.41 | Psoriasis vulgaris | dermatologic | 16 | 0.61 (0.35-1.05) | 0.08 |
| 791 | Gangrene | symptoms | 16 | 0.42 (0.16-1.09) | 0.08 |
| 965.1 | Opiates and related narcotics causing adverse effects in therapeutic use | injuries & poisonings | 16 | 0.44 (0.18-1.09) | 0.08 |
| 253 | Disorders of the pituitary gland and its hypothalamic control | endocrine/metabolic | 16 | 0.30 (0.08-1.14) | 0.08 |
| 737.3 | Kyphoscoliosis and scoliosis | musculoskeletal | 16 | 1.85 (0.93-3.68) | 0.08 |
| 345.1 | Epilepsy | neurological | 16 | 0.52 (0.25-1.08) | 0.08 |
| 471 | Nasal polyps | respiratory | 16 | 1.42 (0.96-2.10) | 0.08 |
| 740.9 | Osteoarthrosis NOS | musculoskeletal | 16 | 1.28 (0.97-1.68) | 0.08 |
| 788 | Syncope and collapse | symptoms | 16 | 0.81 (0.64-1.03) | 0.08 |
| 716.2 | Unspecified monoarthritis | musculoskeletal | 16 | 0.85 (0.70-1.02) | 0.08 |
| 427.11 | Paroxysmal supraventricular tachycardia | circulatory system | 16 | 0.66 (0.42-1.06) | 0.08 |
| 531.1 | Hemorrhage from gastrointestinal ulcer | digestive | 16 | 2.21 (0.90-5.43) | 0.09 |
| 556 | Ulceration of the lower GI tract | digestive | 16 | 0.55 (0.28-1.09) | 0.09 |
| 395 | Heart valve disorders | circulatory system | 16 | 0.74 (0.52-1.04) | 0.09 |
| 575.8 | Other disorders of biliary tract | digestive | 16 | 1.83 (0.92-3.67) | 0.09 |
| 592 | Cystitis and urethritis | genitourinary | 16 | 1.42 (0.95-2.13) | 0.09 |
| 571.81 | Portal hypertension | digestive | 16 | 0.43 (0.16-1.13) | 0.09 |
| 564.8 | Abnormal findings on exam of gastrointestinal tract/ abdominal area | digestive | 16 | 0.53 (0.26-1.10) | 0.09 |
| 275 | Disorders of mineral metabolism | endocrine/metabolic | 16 | 0.66 (0.40-1.07) | 0.09 |
| 389.4 | Tinnitus | sense organs | 16 | 2.33 (0.87-6.24) | 0.09 |
| 550 | Abdominal hernia | digestive | 16 | 0.91 (0.82-1.02) | 0.09 |
| 251 | Other disorders of pancreatic internal secretion | endocrine/metabolic | 16 | 1.87 (0.90-3.87) | 0.09 |
| 415 | Pulmonary heart disease | circulatory system | 16 | 0.67 (0.41-1.07) | 0.09 |
| 317.1 | Alcoholism | mental disorders | 16 | 0.81 (0.64-1.04) | 0.09 |
| 290.2 | Delirium due to conditions classified elsewhere | mental disorders | 16 | 0.48 (0.20-1.14) | 0.10 |
| 729.1 | Rheumatism, unspecified and fibrositis | musculoskeletal | 16 | 0.55 (0.27-1.11) | 0.10 |
| 418.1 | Precordial pain | circulatory system | 16 | 0.73 (0.50-1.06) | 0.10 |
| 274.21 | Chondrocalcinosis | endocrine/metabolic | 16 | 0.45 (0.18-1.16) | 0.10 |
| 1019 | Other ill-defined and unknown causes of morbidity and mortality | symptoms | 16 | 1.17 (0.97-1.40) | 0.10 |
| 198.5 | Secondary malignancy of brain/spine | neoplasms | 16 | 1.94 (0.88-4.26) | 0.10 |
| 701 | Other hypertrophic and atrophic conditions of skin | dermatologic | 16 | 0.76 (0.55-1.05) | 0.10 |
| 747.1 | Cardiac congenital anomalies | congenital anomalies | 16 | 0.69 (0.45-1.07) | 0.10 |
| 80 | Postoperative infection | infectious diseases | 16 | 0.75 (0.54-1.06) | 0.10 |
| 8 | Intestinal infection | infectious diseases | 16 | 1.22 (0.96-1.54) | 0.10 |
| 743.2 | Pathologic fracture | musculoskeletal | 16 | 2.26 (0.85-6.06) | 0.10 |
| 580.14 | Chronic glomerulonephritis, NOS | genitourinary | 16 | 0.53 (0.24-1.14) | 0.10 |
| 172.2 | Other non-epithelial cancer of skin | neoplasms | 16 | 1.20 (0.96-1.49) | 0.10 |
| 747 | Cardiac and circulatory congenital anomalies | congenital anomalies | 16 | 0.70 (0.46-1.08) | 0.11 |
| 208 | Benign neoplasm of colon | neoplasms | 16 | 1.20 (0.96-1.51) | 0.11 |
| 681.5 | Cellulitis and abscess of leg, except foot | dermatologic | 16 | 0.78 (0.58-1.06) | 0.11 |
| 681.3 | Cellulitis and abscess of arm/hand | dermatologic | 16 | 0.78 (0.58-1.06) | 0.11 |
| 530.12 | Ulcer of esophagus | digestive | 16 | 0.77 (0.57-1.06) | 0.11 |
| 728.71 | Contracture of palmar fascia [Dupuytren's disease] | musculoskeletal | 16 | 1.37 (0.93-2.01) | 0.11 |
| 611.3 | Lump or mass in breast | genitourinary | 16 | 1.59 (0.90-2.83) | 0.11 |
| 251.1 | Hypoglycemia | endocrine/metabolic | 16 | 1.81 (0.87-3.75) | 0.11 |
| 585.2 | Renal failure NOS | genitourinary | 16 | 0.51 (0.22-1.18) | 0.12 |
| 202.24 | Large cell lymphoma | neoplasms | 16 | 0.48 (0.19-1.21) | 0.12 |
| 523.31 | Acute periodontitis | digestive | 16 | 1.99 (0.84-4.74) | 0.12 |
| 611 | Abnormal findings on mammogram or breast exam | genitourinary | 16 | 1.57 (0.89-2.76) | 0.12 |
| 728 | Disorders of muscle, ligament, and fascia | musculoskeletal | 16 | 1.31 (0.93-1.84) | 0.12 |
| 244.4 | Hypothyroidism NOS | endocrine/metabolic | 16 | 0.55 (0.26-1.17) | 0.12 |
| 703 | Diseases of nail, NOS | dermatologic | 16 | 1.64 (0.88-3.06) | 0.12 |
| 276.4 | Acid-base balance disorder | endocrine/metabolic | 16 | 0.59 (0.31-1.14) | 0.12 |
| 386 | Vertiginous syndromes and other disorders of vestibular system | sense organs | 16 | 0.80 (0.60-1.06) | 0.12 |
| 728.7 | Fasciitis | musculoskeletal | 16 | 1.34 (0.93-1.93) | 0.12 |
| 793.2 | Nonspecific abnormal findings on radiological and other examination of other intrathoracic organs  (echocardiogram, etc) | circulatory system | 16 | 0.48 (0.19-1.24) | 0.13 |
| 366.2 | Senile cataract | sense organs | 16 | 0.82 (0.64-1.06) | 0.13 |
| 564.1 | Irritable Bowel Syndrome | digestive | 16 | 0.79 (0.58-1.07) | 0.13 |
| 743.11 | Osteoporosis NOS | musculoskeletal | 16 | 0.79 (0.58-1.07) | 0.13 |
| 565 | Anal and rectal conditions | digestive | 16 | 1.15 (0.96-1.39) | 0.13 |
| 386.1 | Meniere's disease | sense organs | 16 | 0.49 (0.19-1.24) | 0.13 |
| 805 | Fracture of vertebral column without mention of spinal cord injury | injuries & poisonings | 16 | 0.63 (0.34-1.15) | 0.13 |
| 695 | Erythematous conditions | dermatologic | 16 | 1.41 (0.90-2.22) | 0.14 |
| 350.2 | Abnormality of gait | neurological | 16 | 0.66 (0.38-1.14) | 0.14 |
| 580.1 | Glomerulonephritis | genitourinary | 16 | 0.51 (0.21-1.25) | 0.14 |
| 522 | Diseases of pulp and periapical tissues | digestive | 16 | 0.67 (0.40-1.14) | 0.14 |
| 742.8 | Articular cartilage disorder | musculoskeletal | 16 | 0.47 (0.17-1.28) | 0.14 |
| 276.1 | Electrolyte imbalance | endocrine/metabolic | 16 | 0.77 (0.54-1.09) | 0.14 |
| 285 | Other anemias | hematopoietic | 16 | 0.86 (0.70-1.05) | 0.14 |
| 560.3 | Peritoneal or intestinal adhesions | digestive | 16 | 0.56 (0.26-1.21) | 0.14 |
| 385.3 | Cholesteatoma | sense organs | 16 | 0.51 (0.21-1.26) | 0.14 |
| 939 | Atopic/contact dermatitis due to other or unspecified | dermatologic | 16 | 1.44 (0.88-2.35) | 0.14 |
| 572 | Ascites (non malignant) | digestive | 16 | 0.66 (0.37-1.16) | 0.14 |
| 157 | Pancreatic cancer | neoplasms | 16 | 1.98 (0.79-4.97) | 0.14 |
| 698 | Pruritus and related conditions | dermatologic | 16 | 1.81 (0.81-4.01) | 0.15 |
| 497 | Bronchitis | respiratory | 16 | 0.52 (0.21-1.26) | 0.15 |
| 741.4 | Joint effusions | musculoskeletal | 16 | 0.65 (0.36-1.17) | 0.15 |
| 276 | Disorders of fluid, electrolyte, and acid-base balance | endocrine/metabolic | 16 | 0.82 (0.63-1.07) | 0.15 |
| 443 | Peripheral vascular disease | circulatory system | 16 | 0.77 (0.54-1.10) | 0.15 |
| 994 | Sepsis and SIRS | infectious diseases | 16 | 0.74 (0.48-1.12) | 0.15 |
| 994.2 | Sepsis | infectious diseases | 16 | 0.74 (0.48-1.12) | 0.15 |
| 381.1 | Otitis media | sense organs | 16 | 0.68 (0.40-1.16) | 0.16 |
| 773 | Pain in limb | symptoms | 16 | 0.81 (0.61-1.08) | 0.16 |
| 696.42 | Psoriatic arthropathy | dermatologic | 16 | 0.44 (0.14-1.37) | 0.16 |
| 332 | Parkinson's disease | neurological | 16 | 1.61 (0.83-3.14) | 0.16 |
| 742 | Derangement of joint, nontraumatic | musculoskeletal | 16 | 1.35 (0.89-2.04) | 0.16 |
| 530.11 | GERD | digestive | 16 | 0.87 (0.72-1.06) | 0.16 |
| 695.7 | Prurigo and Lichen | dermatologic | 16 | 1.78 (0.80-3.96) | 0.16 |
| 159 | Malignant neoplasm of other and ill-defined sites within the digestive organs and peritoneum | neoplasms | 16 | 0.81 (0.60-1.09) | 0.16 |
| 158 | Neoplasm of unspecified nature of digestive system | neoplasms | 16 | 1.63 (0.82-3.24) | 0.16 |
| 596.5 | Functional disorders of bladder | genitourinary | 16 | 0.68 (0.39-1.17) | 0.16 |
| 276.41 | Acidosis | endocrine/metabolic | 16 | 0.62 (0.31-1.22) | 0.17 |
| 561.2 | Flatulence | digestive | 16 | 0.68 (0.39-1.17) | 0.17 |
| 244 | Hypothyroidism | endocrine/metabolic | 16 | 0.59 (0.28-1.25) | 0.17 |
| 225.1 | Benign neoplasm of brain, cranial nerves, meninges | neoplasms | 16 | 0.57 (0.25-1.27) | 0.17 |
| 803.2 | Fracture of radius and ulna | injuries & poisonings | 16 | 1.25 (0.91-1.70) | 0.17 |
| 452 | Other venous embolism and thrombosis | circulatory system | 16 | 1.93 (0.75-4.97) | 0.17 |
| 599.3 | Dysuria | genitourinary | 16 | 0.64 (0.33-1.22) | 0.17 |
| 426.32 | Left bundle branch block | circulatory system | 16 | 0.69 (0.40-1.18) | 0.17 |
| 426.9 | Cardiac pacemaker/device in situ | circulatory system | 16 | 0.73 (0.47-1.15) | 0.17 |
| 577 | Diseases of pancreas | digestive | 16 | 1.35 (0.88-2.08) | 0.17 |
| 379.3 | Aphakia and other disorders of lens | sense organs | 16 | 0.69 (0.41-1.18) | 0.18 |
| 598 | Abnormal findings on examination of urine | genitourinary | 16 | 1.44 (0.84-2.47) | 0.18 |
| 444 | Arterial embolism and thrombosis | circulatory system | 16 | 0.52 (0.20-1.36) | 0.18 |
| 743.1 | Osteoporosis | musculoskeletal | 16 | 0.82 (0.62-1.09) | 0.18 |
| 916 | Contusion | injuries & poisonings | 16 | 0.67 (0.38-1.20) | 0.18 |
| 229 | Benign neoplasm of unspecified sites | neoplasms | 16 | 0.73 (0.47-1.16) | 0.18 |
| 596 | Other disorders of bladder | genitourinary | 16 | 0.86 (0.68-1.08) | 0.19 |
| 261.2 | Vitamin B-complex deficiencies | endocrine/metabolic | 16 | 0.58 (0.26-1.31) | 0.19 |
| 454.1 | Varicose veins of lower extremity | circulatory system | 16 | 0.87 (0.70-1.07) | 0.19 |
| 530.5 | Disorders of esophageal motility | digestive | 16 | 1.80 (0.74-4.37) | 0.19 |
| 681.7 | Cellulitis and abscess of trunk | dermatologic | 16 | 0.55 (0.22-1.36) | 0.19 |
| 381 | Otitis media and Eustachian tube disorders | sense organs | 16 | 0.73 (0.46-1.17) | 0.19 |
| 292.1 | Aphasia/speech disturbance | mental disorders | 16 | 0.68 (0.38-1.21) | 0.19 |
| 591 | Urinary tract infection | genitourinary | 16 | 0.83 (0.63-1.10) | 0.20 |
| 293.1 | Swelling, mass, or lump in head and neck [Space occupying lesion, intracranial NOS] | mental disorders | 16 | 1.63 (0.78-3.41) | 0.20 |
| 729 | Other disorders of soft tissues | musculoskeletal | 16 | 0.83 (0.62-1.11) | 0.20 |
| 361.1 | Retinal detachment with retinal defect | sense organs | 16 | 1.48 (0.81-2.69) | 0.21 |
| 274.2 | Crystal arthropathies | endocrine/metabolic | 16 | 0.56 (0.23-1.38) | 0.21 |
| 580 | Nephritis; nephrosis; renal sclerosis | genitourinary | 16 | 0.69 (0.39-1.23) | 0.21 |
| 555 | Inflammatory bowel disease and other gastroenteritis and colitis | digestive | 16 | 1.41 (0.83-2.42) | 0.21 |
| 530.1 | Esophagitis, GERD and related diseases | digestive | 16 | 0.92 (0.80-1.05) | 0.21 |
| 523 | Gingival and periodontal diseases | digestive | 16 | 1.41 (0.83-2.41) | 0.21 |
| 174 | Breast cancer | neoplasms | 16 | 1.23 (0.89-1.71) | 0.21 |
| 455 | Hemorrhoids | circulatory system | 16 | 0.91 (0.78-1.06) | 0.21 |
| 255 | Disorders of adrenal glands | endocrine/metabolic | 16 | 0.57 (0.24-1.38) | 0.21 |
| 592.12 | Chronic cystitis | genitourinary | 16 | 1.61 (0.76-3.39) | 0.21 |
| 200 | Myeloproliferative disease | neoplasms | 16 | 0.54 (0.21-1.43) | 0.22 |
| 702.2 | Seborrheic keratosis | dermatologic | 16 | 1.29 (0.86-1.93) | 0.22 |
| 1011 | Complications of surgical and medical procedures | injuries & poisonings | 16 | 0.86 (0.68-1.09) | 0.22 |
| 716.1 | Unspecified polyarthropathy or polyarthritis | musculoskeletal | 16 | 1.39 (0.82-2.35) | 0.23 |
| 550.2 | Diaphragmatic hernia | digestive | 16 | 0.91 (0.79-1.06) | 0.23 |
| 241 | Nontoxic nodular goiter | endocrine/metabolic | 16 | 0.67 (0.34-1.29) | 0.23 |
| 766 | Neuralgia, neuritis, and radiculitis NOS | symptoms | 16 | 1.49 (0.78-2.86) | 0.23 |
| 280.1 | Iron deficiency anemias, unspecified or not due to blood loss | hematopoietic | 16 | 1.17 (0.90-1.53) | 0.23 |
| 295 | Schizophrenia and other psychotic disorders | mental disorders | 16 | 1.59 (0.74-3.43) | 0.23 |
| 444.1 | Arterial embolism and thrombosis of lower extremity artery | circulatory system | 16 | 0.56 (0.22-1.45) | 0.23 |
| 523.32 | Chronic periodontitis | digestive | 16 | 1.75 (0.68-4.50) | 0.24 |
| 317 | Alcohol-related disorders | mental disorders | 16 | 0.85 (0.64-1.12) | 0.24 |
| 506 | Empyema and pneumothorax | respiratory | 16 | 1.47 (0.77-2.82) | 0.24 |
| 351 | Other peripheral nerve disorders | neurological | 16 | 0.89 (0.72-1.09) | 0.25 |
| 689 | Disorder of skin and subcutaneous tissue NOS | dermatologic | 16 | 1.19 (0.88-1.59) | 0.25 |
| 348 | Other conditions of brain | neurological | 16 | 0.71 (0.39-1.29) | 0.26 |
| 761 | Cervicalgia | symptoms | 16 | 0.74 (0.44-1.26) | 0.26 |
| 586.2 | Cyst of kidney, acquired | genitourinary | 16 | 1.43 (0.76-2.68) | 0.27 |
| 803.3 | Fracture of clavicle or scapula | injuries & poisonings | 16 | 0.72 (0.41-1.28) | 0.27 |
| 568.1 | Peritoneal adhesions (postoperative) (postinfection) | digestive | 16 | 1.25 (0.84-1.87) | 0.27 |
| 946 | Anaphylactic shock NOS | injuries & poisonings | 16 | 0.59 (0.23-1.53) | 0.27 |
| 530.9 | Heartburn | digestive | 16 | 0.76 (0.47-1.24) | 0.28 |
| 563 | Constipation | digestive | 16 | 1.13 (0.90-1.42) | 0.28 |
| 334 | Degenerative disease of the spinal cord | neurological | 16 | 0.75 (0.44-1.27) | 0.28 |
| 300 | Anxiety disorders | mental disorders | 16 | 0.86 (0.66-1.13) | 0.28 |
| 285.2 | Anemia of chronic disease | hematopoietic | 16 | 1.96 (0.57-6.69) | 0.28 |
| 782 | Symptoms involving skin and other integumentary tissue | symptoms | 16 | 0.75 (0.44-1.27) | 0.28 |
| 381.11 | Suppurative and unspecified otitis media | sense organs | 16 | 0.66 (0.31-1.42) | 0.29 |
| 733.4 | Aseptic necrosis of bone | musculoskeletal | 16 | 0.59 (0.23-1.55) | 0.29 |
| 250.1 | Type 1 diabetes | endocrine/metabolic | 16 | 0.71 (0.37-1.34) | 0.29 |
| 735.23 | Hallux rigidus | musculoskeletal | 16 | 0.74 (0.42-1.30) | 0.29 |
| 568 | Other disorders of peritoneum | digestive | 16 | 1.23 (0.84-1.82) | 0.29 |
| 783 | Fever of unknown origin | symptoms | 16 | 0.77 (0.47-1.26) | 0.29 |
| 401 | Hypertension | circulatory system | 16 | 1.15 (0.88-1.50) | 0.29 |
| 740.2 | Osteoarthrosis, generalized | musculoskeletal | 16 | 1.63 (0.65-4.06) | 0.29 |
| 525 | Other diseases of the teeth and supporting structures | digestive | 16 | 1.26 (0.82-1.93) | 0.30 |
| 556.1 | Ulceration of intestine | digestive | 16 | 0.63 (0.27-1.50) | 0.30 |
| 8.6 | Viral Enteritis | infectious diseases | 16 | 1.49 (0.70-3.19) | 0.30 |
| 41.2 | Streptococcus infection | infectious diseases | 16 | 0.75 (0.43-1.30) | 0.31 |
| 41.1 | Staphylococcus infections | infectious diseases | 16 | 0.81 (0.55-1.22) | 0.31 |
| 738.4 | Acquired spondylolisthesis | musculoskeletal | 16 | 0.75 (0.42-1.32) | 0.31 |
| 411.41 | Aneurysm and dissection of heart | circulatory system | 16 | 0.65 (0.28-1.51) | 0.32 |
| 368.9 | Subjective visual disturbances | sense organs | 16 | 0.63 (0.26-1.55) | 0.32 |
| 339 | Other headache syndromes | neurological | 16 | 0.88 (0.68-1.13) | 0.32 |
| 756 | Other congenital musculoskeletal anomalies | congenital anomalies | 16 | 1.59 (0.63-3.99) | 0.32 |
| 727.4 | Ganglion and cyst of synovium, tendon, and bursa | musculoskeletal | 16 | 0.82 (0.55-1.22) | 0.32 |
| 384 | Other disorders of tympanic membrane | sense organs | 16 | 0.74 (0.40-1.35) | 0.32 |
| 327 | Sleep disorders | neurological | 16 | 0.85 (0.62-1.17) | 0.32 |
| 275.5 | Disorders of calcium/phosphorus metabolism | endocrine/metabolic | 16 | 0.62 (0.24-1.61) | 0.32 |
| 401.1 | Essential hypertension | circulatory system | 16 | 0.87 (0.67-1.14) | 0.33 |
| 443.1 | Raynaud's syndrome | circulatory system | 16 | 1.39 (0.72-2.69) | 0.33 |
| 430 | Intracranial hemorrhage | circulatory system | 16 | 0.77 (0.45-1.30) | 0.33 |
| 327.3 | Sleep apnea | neurological | 16 | 0.84 (0.60-1.19) | 0.33 |
| 836 | Traumatic arthropathy | injuries & poisonings | 16 | 0.62 (0.23-1.63) | 0.33 |
| 555.21 | Ulcerative colitis (chronic) | digestive | 16 | 1.61 (0.61-4.23) | 0.33 |
| 521.1 | Dental caries | digestive | 16 | 0.82 (0.54-1.23) | 0.33 |
| 300.1 | Anxiety disorder | mental disorders | 16 | 0.87 (0.65-1.16) | 0.33 |
| 1005 | Other symptoms | symptoms | 16 | 1.37 (0.72-2.59) | 0.34 |
| 367 | Disorders of refraction and accommodation; blindness and low vision | sense organs | 16 | 0.80 (0.51-1.26) | 0.34 |
| 416 | Cardiomegaly | circulatory system | 16 | 0.81 (0.52-1.26) | 0.34 |
| 198.3 | Secondary malignant neoplasm of digestive systems | neoplasms | 16 | 1.32 (0.75-2.34) | 0.34 |
| 275.1 | Disorders of iron metabolism | hematopoietic | 16 | 0.67 (0.30-1.52) | 0.34 |
| 531.2 | Gastric ulcer | digestive | 16 | 0.84 (0.59-1.20) | 0.35 |
| 202.2 | Non-Hodgkins lymphoma | neoplasms | 16 | 0.78 (0.46-1.32) | 0.35 |
| 79 | Viral infection | infectious diseases | 16 | 1.22 (0.80-1.86) | 0.35 |
| 751.1 | Congenital anomalies of genital organs | congenital anomalies | 16 | 1.48 (0.65-3.40) | 0.35 |
| 353 | Nerve root and plexus disorders | neurological | 16 | 1.37 (0.70-2.66) | 0.35 |
| 915 | Superficial injury without mention of infection | injuries & poisonings | 16 | 1.17 (0.83-1.65) | 0.36 |
| 112 | Candidiasis | infectious diseases | 16 | 0.80 (0.49-1.29) | 0.36 |
| 465.2 | Acute pharyngitis | respiratory | 16 | 1.42 (0.67-3.04) | 0.36 |
| 149 | Cancer of larynx, pharynx, nasal cavities | neoplasms | 16 | 0.66 (0.27-1.61) | 0.36 |
| 736 | Other acquired deformities of limbs | musculoskeletal | 16 | 0.77 (0.45-1.34) | 0.36 |
| 966 | Poisoning by anticonvulsants and anti Parkinsonism drugs | injuries & poisonings | 16 | 0.63 (0.23-1.70) | 0.36 |
| 198.4 | Secondary malignant neoplasm of liver | neoplasms | 16 | 1.23 (0.79-1.90) | 0.36 |
| 278 | Overweight, obesity and other hyperalimentation | endocrine/metabolic | 16 | 0.86 (0.61-1.20) | 0.36 |
| 514 | Abnormal findings examination of lungs | respiratory | 16 | 0.81 (0.52-1.27) | 0.36 |
| 564 | Functional digestive disorders | digestive | 16 | 1.10 (0.89-1.37) | 0.37 |
| 521 | Diseases of hard tissues of teeth | digestive | 16 | 0.83 (0.55-1.25) | 0.37 |
| 871 | Open wounds of extremities | injuries & poisonings | 16 | 1.17 (0.83-1.66) | 0.37 |
| 446 | Polyarteritis nodosa and allied conditions | circulatory system | 16 | 0.63 (0.22-1.76) | 0.38 |
| 374 | Other disorders of eyelids | sense organs | 16 | 1.14 (0.85-1.54) | 0.38 |
| 579.8 | Nonspecific abnormal findings in stool contents | digestive | 16 | 0.78 (0.45-1.35) | 0.38 |
| 531 | Peptic ulcer (excl. esophageal) | digestive | 16 | 0.89 (0.68-1.16) | 0.38 |
| 707.1 | Decubitus ulcer | dermatologic | 16 | 0.72 (0.34-1.50) | 0.38 |
| 733 | Other disorders of bone and cartilage | musculoskeletal | 16 | 0.85 (0.59-1.22) | 0.38 |
| 289.4 | Lymphadenitis | hematopoietic | 16 | 1.22 (0.79-1.88) | 0.38 |
| 513 | Respiratory abnormalities | respiratory | 16 | 1.50 (0.61-3.70) | 0.38 |
| 250.7 | Diabetic retinopathy | endocrine/metabolic | 16 | 1.32 (0.71-2.43) | 0.38 |
| 365.1 | Open-angle glaucoma | sense organs | 16 | 1.36 (0.68-2.73) | 0.38 |
| 764 | Sciatica | symptoms | 16 | 0.82 (0.51-1.29) | 0.38 |
| 571 | Chronic liver disease and cirrhosis | digestive | 16 | 0.77 (0.43-1.38) | 0.39 |
| 809 | Fracture of unspecified bones | injuries & poisonings | 16 | 1.31 (0.71-2.44) | 0.39 |
| 577.2 | Chronic pancreatitis | digestive | 16 | 1.54 (0.58-4.13) | 0.39 |
| 426.91 | Cardiac pacemaker in situ | circulatory system | 16 | 0.81 (0.51-1.30) | 0.39 |
| 722.9 | Other and unspecified disc disorder | musculoskeletal | 16 | 1.16 (0.83-1.63) | 0.39 |
| 189 | Cancer of urinary organs (incl. kidney and bladder) | neoplasms | 16 | 0.86 (0.60-1.22) | 0.39 |
| 379 | Other disorders of eye | sense organs | 16 | 0.86 (0.60-1.23) | 0.39 |
| 414 | Other forms of chronic heart disease | circulatory system | 16 | 0.79 (0.47-1.35) | 0.40 |
| 288.1 | Decreased white blood cell count | hematopoietic | 16 | 1.19 (0.80-1.76) | 0.40 |
| 288.11 | Neutropenia | hematopoietic | 16 | 1.19 (0.80-1.76) | 0.40 |
| 278.1 | Obesity | endocrine/metabolic | 16 | 0.86 (0.62-1.21) | 0.40 |
| 365.11 | Primary open angle glaucoma | sense organs | 16 | 1.35 (0.67-2.71) | 0.40 |
| 350 | Abnormal movement | neurological | 16 | 0.83 (0.54-1.28) | 0.40 |
| 202 | Cancer of other lymphoid, histiocytic tissue | neoplasms | 16 | 0.82 (0.51-1.31) | 0.41 |
| 396 | Abnormal heart sounds | circulatory system | 16 | 0.75 (0.37-1.49) | 0.41 |
| 850 | Hemorrhage or hematoma complicating a procedure | injuries & poisonings | 16 | 1.19 (0.79-1.79) | 0.41 |
| 41.4 | E. coli | infectious diseases | 16 | 1.20 (0.78-1.84) | 0.41 |
| 335 | Multiple sclerosis | neurological | 16 | 1.29 (0.70-2.38) | 0.41 |
| 364 | Corneal opacity and other disorders of cornea | sense organs | 16 | 1.41 (0.62-3.21) | 0.42 |
| 714.1 | Rheumatoid arthritis | musculoskeletal | 16 | 0.80 (0.46-1.38) | 0.42 |
| 389 | Hearing loss | sense organs | 16 | 1.15 (0.81-1.63) | 0.42 |
| 530.2 | Esophageal bleeding (varices/hemorrhage) | digestive | 16 | 0.80 (0.46-1.38) | 0.43 |
| 357 | Inflammatory and toxic neuropathy | neurological | 16 | 0.79 (0.43-1.44) | 0.43 |
| 687.1 | Rash and other nonspecific skin eruption | dermatologic | 16 | 0.83 (0.51-1.34) | 0.43 |
| 280 | Iron deficiency anemias | hematopoietic | 16 | 1.11 (0.86-1.43) | 0.43 |
| 38.1 | Gram negative septicemia | infectious diseases | 16 | 0.74 (0.34-1.60) | 0.44 |
| 550.4 | Umbilical hernia | digestive | 16 | 1.16 (0.80-1.68) | 0.44 |
| 367.9 | Blindness and low vision | sense organs | 16 | 1.38 (0.60-3.17) | 0.44 |
| 70 | Viral hepatitis | infectious diseases | 16 | 0.78 (0.41-1.48) | 0.44 |
| 743 | Osteoporosis, osteopenia and pathological fracture | musculoskeletal | 16 | 0.90 (0.70-1.17) | 0.45 |
| 804 | Fracture of hand or wrist | injuries & poisonings | 16 | 0.86 (0.59-1.27) | 0.45 |
| 747.11 | Cardiac shunt/ heart septal defect | congenital anomalies | 16 | 1.43 (0.57-3.58) | 0.45 |
| 594.3 | Calculus of ureter | genitourinary | 16 | 1.19 (0.75-1.89) | 0.45 |
| 81 | Infection/inflammation of internal prosthetic device; implant; and graft | infectious diseases | 16 | 0.84 (0.54-1.32) | 0.45 |
| 535.1 | Acute gastritis | digestive | 16 | 1.28 (0.67-2.46) | 0.45 |
| 686 | Other local infections of skin and subcutaneous tissue | dermatologic | 16 | 0.88 (0.63-1.23) | 0.46 |
| 250.24 | Type 2 diabetes with neurological manifestations | endocrine/metabolic | 16 | 0.70 (0.28-1.78) | 0.46 |
| 527 | Diseases of the salivary glands | digestive | 16 | 1.37 (0.59-3.17) | 0.46 |
| 430.1 | Subarachnoid hemorrhage | circulatory system | 16 | 0.74 (0.34-1.63) | 0.46 |
| 204.12 | Lymphoid leukemia, chronic | neoplasms | 16 | 0.69 (0.26-1.86) | 0.47 |
| 427.9 | Palpitations | circulatory system | 16 | 0.87 (0.61-1.26) | 0.47 |
| 743.9 | Osteopenia or other disorder of bone and cartilage | musculoskeletal | 16 | 1.34 (0.61-2.92) | 0.47 |
| 378.1 | Strabismus (not specified as paralytic) | sense organs | 16 | 0.70 (0.27-1.83) | 0.47 |
| 250.23 | Type 2 diabetes with ophthalmic manifestations | endocrine/metabolic | 16 | 1.26 (0.67-2.34) | 0.47 |
| 800 | Fracture of lower limb | injuries & poisonings | 16 | 0.91 (0.70-1.18) | 0.47 |
| 211 | Benign neoplasm of other parts of digestive system | neoplasms | 16 | 0.84 (0.51-1.36) | 0.47 |
| 540.11 | Acute appendicitis | digestive | 16 | 1.17 (0.76-1.83) | 0.48 |
| 578 | Gastrointestinal hemorrhage | digestive | 16 | 0.95 (0.81-1.10) | 0.48 |
| 681.1 | Cellulitis and abscess of fingers/toes | dermatologic | 16 | 1.40 (0.55-3.57) | 0.48 |
| 189.11 | Malignant neoplasm of kidney, except pelvis | neoplasms | 16 | 0.78 (0.38-1.57) | 0.48 |
| 450 | Noninfectious disorders of lymphatic channels | circulatory system | 16 | 1.35 (0.58-3.13) | 0.49 |
| 716.9 | Arthropathy NOS | musculoskeletal | 16 | 1.05 (0.92-1.19) | 0.49 |
| 290.1 | Dementias | mental disorders | 16 | 0.78 (0.38-1.59) | 0.49 |
| 579 | Other symptoms involving abdomen and pelvis | digestive | 16 | 0.87 (0.59-1.29) | 0.49 |
| 745 | Pain in joint | musculoskeletal | 16 | 0.91 (0.71-1.18) | 0.49 |
| 474.2 | Chronic tonsillitis and adenoiditis | respiratory | 16 | 1.42 (0.52-3.90) | 0.49 |
| 800.1 | Fracture of neck of femur | injuries & poisonings | 16 | 0.77 (0.36-1.63) | 0.49 |
| 715 | Other inflammatory spondylopathies | musculoskeletal | 16 | 0.83 (0.48-1.43) | 0.50 |
| 369 | Infection of the eye | sense organs | 16 | 1.38 (0.54-3.53) | 0.51 |
| 512.8 | Cough | respiratory | 16 | 1.15 (0.76-1.75) | 0.51 |
| 252.1 | Hyperparathyroidism | endocrine/metabolic | 16 | 0.77 (0.34-1.71) | 0.51 |
| 252 | Disorders of parathyroid gland | endocrine/metabolic | 16 | 0.78 (0.37-1.66) | 0.52 |
| 770 | Myalgia and myositis unspecified | symptoms | 16 | 1.34 (0.55-3.23) | 0.52 |
| 199 | Neoplasm of uncertain behavior | neoplasms | 16 | 0.82 (0.45-1.49) | 0.52 |
| 727.5 | Rupture of synovium | musculoskeletal | 16 | 1.35 (0.54-3.38) | 0.52 |
| 368 | Visual disturbances | sense organs | 16 | 0.88 (0.60-1.30) | 0.52 |
| 240 | Simple and unspecified goiter | endocrine/metabolic | 16 | 1.35 (0.54-3.34) | 0.52 |
| 715.2 | Ankylosing spondylitis | musculoskeletal | 16 | 1.34 (0.54-3.29) | 0.52 |
| 470 | Septal Deviations/Turbinate Hypertrophy | respiratory | 16 | 0.87 (0.58-1.33) | 0.53 |
| 367.1 | Myopia | sense organs | 16 | 0.81 (0.43-1.53) | 0.53 |
| 714 | Rheumatoid arthritis and other inflammatory polyarthropathies | musculoskeletal | 16 | 0.85 (0.51-1.41) | 0.53 |
| 288 | Diseases of white blood cells | hematopoietic | 16 | 1.12 (0.78-1.61) | 0.53 |
| 726 | Peripheral enthesopathies and allied syndromes | musculoskeletal | 16 | 1.06 (0.88-1.28) | 0.53 |
| 250.2 | Type 2 diabetes | endocrine/metabolic | 16 | 1.13 (0.76-1.70) | 0.54 |
| 420.3 | Endocarditis | circulatory system | 16 | 0.77 (0.33-1.81) | 0.55 |
| 590 | Pyelonephritis | genitourinary | 16 | 0.83 (0.45-1.54) | 0.55 |
| 751 | Genitourinary congenital anomalies | congenital anomalies | 16 | 0.84 (0.48-1.48) | 0.55 |
| 557 | Intestinal malabsorption (non-celiac) | digestive | 16 | 1.33 (0.52-3.40) | 0.55 |
| 853 | Complication of colostomy or enterostomy | injuries & poisonings | 16 | 1.35 (0.50-3.66) | 0.55 |
| 537 | Other disorders of stomach and duodenum | digestive | 16 | 0.89 (0.61-1.31) | 0.56 |
| 741 | Symptoms and disorders of the joints | musculoskeletal | 16 | 1.12 (0.77-1.62) | 0.56 |
| 291 | Other specified nonpsychotic and/or transient mental disorders | mental disorders | 16 | 1.30 (0.54-3.15) | 0.56 |
| 276.13 | Hyperpotassemia | endocrine/metabolic | 16 | 0.81 (0.39-1.65) | 0.56 |
| 386.3 | Labyrinthitis | sense organs | 16 | 0.79 (0.35-1.76) | 0.56 |
| 344 | Other paralytic syndromes | neurological | 16 | 0.77 (0.32-1.84) | 0.56 |
| 574.11 | Cholelithiasis with acute cholecystitis | digestive | 16 | 1.28 (0.55-2.99) | 0.56 |
| 802 | Fracture of pelvis | injuries & poisonings | 16 | 1.28 (0.56-2.94) | 0.56 |
| 574.2 | Calculus of bile duct | digestive | 16 | 1.14 (0.73-1.76) | 0.57 |
| 722 | Intervertebral disc disorders | musculoskeletal | 16 | 0.93 (0.73-1.18) | 0.57 |
| 345.3 | Convulsions | neurological | 16 | 0.83 (0.44-1.58) | 0.57 |
| 722.6 | Degeneration of intervertebral disc | musculoskeletal | 16 | 0.89 (0.58-1.35) | 0.57 |
| 694 | Dyschromia and Vitiligo | dermatologic | 16 | 0.82 (0.40-1.66) | 0.58 |
| 198.6 | Secondary malignancy of bone | neoplasms | 16 | 0.87 (0.54-1.41) | 0.58 |
| 716 | Other arthropathies | musculoskeletal | 16 | 1.05 (0.88-1.25) | 0.58 |
| 586.4 | Stricture/obstruction of ureter | genitourinary | 16 | 0.81 (0.39-1.70) | 0.58 |
| 227 | Benign neoplasm of other endocrine glands and related structures | neoplasms | 16 | 1.23 (0.58-2.62) | 0.58 |
| 743.13 | Other specified osteoporosis | musculoskeletal | 16 | 0.76 (0.29-2.01) | 0.59 |
| 573 | Other disorders of liver | digestive | 16 | 0.92 (0.69-1.24) | 0.59 |
| 402 | Elevated blood pressure reading without diagnosis of hypertension | circulatory system | 16 | 0.81 (0.37-1.75) | 0.59 |
| 569 | Other disorders of intestine | digestive | 16 | 1.10 (0.78-1.56) | 0.59 |
| 191.1 | Cancer of brain and nervous system | neoplasms | 16 | 1.30 (0.49-3.43) | 0.60 |
| 350.1 | Abnormal involuntary movements | neurological | 16 | 0.82 (0.39-1.73) | 0.60 |
| 782.3 | Edema | symptoms | 16 | 0.86 (0.50-1.50) | 0.60 |
| 217 | Vascular hamartomas and non-neoplastic nevi | neoplasms | 16 | 1.27 (0.53-3.05) | 0.60 |
| 710 | Osteomyelitis, periostitis, and other infections involving bone | musculoskeletal | 16 | 0.79 (0.32-1.94) | 0.61 |
| 724.9 | Other unspecified back disorders | musculoskeletal | 16 | 0.86 (0.50-1.51) | 0.61 |
| 858 | Complication of internal orthopedic device | injuries & poisonings | 16 | 0.90 (0.61-1.34) | 0.61 |
| 420.2 | Pericarditis | circulatory system | 16 | 0.85 (0.45-1.59) | 0.62 |
| 800.3 | Fracture of tibia and fibula | injuries & poisonings | 16 | 1.13 (0.70-1.83) | 0.62 |
| 599 | Other symptoms/disorders or the urinary system | genitourinary | 16 | 1.05 (0.86-1.29) | 0.62 |
| 260.6 | Anorexia | endocrine/metabolic | 16 | 1.21 (0.56-2.61) | 0.63 |
| 801 | Fracture of ankle and foot | injuries & poisonings | 16 | 0.89 (0.56-1.42) | 0.63 |
| 687 | Symptoms affecting skin | dermatologic | 16 | 0.93 (0.69-1.25) | 0.63 |
| 535.6 | Duodenitis | digestive | 16 | 0.94 (0.72-1.22) | 0.64 |
| 560 | Intestinal obstruction without mention of hernia | digestive | 16 | 1.09 (0.76-1.55) | 0.64 |
| 528 | Diseases of the oral soft tissues, excluding lesions specific for gingiva and tongue | digestive | 16 | 0.92 (0.64-1.32) | 0.65 |
| 415.2 | Chronic pulmonary heart disease | circulatory system | 16 | 0.81 (0.32-2.03) | 0.65 |
| 531.3 | Duodenal ulcer | digestive | 16 | 0.91 (0.60-1.37) | 0.65 |
| 540.1 | Appendicitis | digestive | 16 | 1.10 (0.74-1.63) | 0.65 |
| 529 | Diseases and other conditions of the tongue | digestive | 16 | 1.16 (0.61-2.19) | 0.65 |
| 726.3 | Bursitis | musculoskeletal | 16 | 0.84 (0.39-1.80) | 0.65 |
| 362.4 | Retinal vascular changes and abnomalities | sense organs | 16 | 1.19 (0.55-2.57) | 0.65 |
| 472 | Chronic pharyngitis and nasopharyngitis | respiratory | 16 | 1.18 (0.57-2.42) | 0.65 |
| 425.1 | Primary/intrinsic cardiomyopathies | circulatory system | 16 | 1.16 (0.61-2.21) | 0.66 |
| 379.2 | Disorders of vitreous body | sense organs | 16 | 0.87 (0.48-1.60) | 0.66 |
| 153.2 | Colon cancer | neoplasms | 16 | 0.89 (0.52-1.52) | 0.66 |
| 586 | Other disorders of the kidney and ureters | genitourinary | 16 | 0.92 (0.62-1.36) | 0.68 |
| 296.1 | Bipolar | mental disorders | 16 | 0.86 (0.43-1.72) | 0.68 |
| 574.12 | Cholelithiasis with other cholecystitis | digestive | 16 | 1.12 (0.65-1.93) | 0.68 |
| 204.1 | Lymphoid leukemia | neoplasms | 16 | 0.82 (0.32-2.08) | 0.68 |
| 204.4 | Multiple myeloma | neoplasms | 16 | 1.22 (0.47-3.15) | 0.68 |
| 594.1 | Calculus of kidney | genitourinary | 16 | 0.92 (0.62-1.37) | 0.69 |
| 451 | Phlebitis and thrombophlebitis | circulatory system | 16 | 0.89 (0.49-1.59) | 0.69 |
| 151 | Cancer of stomach | neoplasms | 16 | 0.82 (0.32-2.13) | 0.69 |
| 599.5 | Frequency of urination and polyuria | genitourinary | 16 | 0.93 (0.65-1.33) | 0.69 |
| 555.1 | Regional enteritis | digestive | 16 | 0.90 (0.52-1.54) | 0.69 |
| 465 | Acute upper respiratory infections of multiple or unspecified sites | respiratory | 16 | 1.10 (0.69-1.74) | 0.69 |
| 1008 | Crushing or internal injury to organs | injuries & poisonings | 16 | 0.87 (0.43-1.75) | 0.69 |
| 427.2 | Atrial fibrillation and flutter | circulatory system | 16 | 0.96 (0.79-1.17) | 0.69 |
| 528.5 | Diseases of lips | digestive | 16 | 0.84 (0.36-1.98) | 0.70 |
| 352 | Disorders of other cranial nerves | neurological | 16 | 0.89 (0.49-1.62) | 0.70 |
| 573.5 | Jaundice (not of newborn) | digestive | 16 | 1.13 (0.62-2.04) | 0.70 |
| 575.7 | Other disorders of gallbladder | digestive | 16 | 1.13 (0.62-2.04) | 0.70 |
| 394.2 | Mitral valve disease | circulatory system | 16 | 1.08 (0.72-1.64) | 0.70 |
| 303 | Psychogenic and somatoform disorders | mental disorders | 16 | 0.83 (0.31-2.18) | 0.70 |
| 277 | Other disorders of metabolism | endocrine/metabolic | 16 | 0.89 (0.49-1.61) | 0.70 |
| 803 | Fracture of upper limb | injuries & poisonings | 16 | 0.95 (0.74-1.22) | 0.70 |
| 78 | Viral warts & HPV | infectious diseases | 16 | 0.87 (0.43-1.76) | 0.70 |
| 510 | Other diseases of lung | respiratory | 16 | 1.17 (0.52-2.60) | 0.71 |
| 281.1 | Megaloblastic anemia | hematopoietic | 16 | 0.88 (0.44-1.74) | 0.71 |
| 292.3 | Memory loss | mental disorders | 16 | 0.85 (0.37-1.98) | 0.71 |
| 427.7 | Tachycardia NOS | circulatory system | 16 | 0.91 (0.57-1.48) | 0.71 |
| 593 | Hematuria | genitourinary | 16 | 1.03 (0.86-1.24) | 0.71 |
| 475 | Chronic sinusitis | respiratory | 16 | 0.92 (0.59-1.43) | 0.72 |
| 189.1 | Cancer of kidney and renal pelvis | neoplasms | 16 | 0.88 (0.44-1.76) | 0.72 |
| 395.1 | Nonrheumatic mitral valve disorders | circulatory system | 16 | 1.08 (0.71-1.64) | 0.72 |
| 41 | Bacterial infection NOS | infectious diseases | 16 | 0.96 (0.79-1.18) | 0.72 |
| 411.9 | Other acute and subacute forms of ischemic heart disease | circulatory system | 16 | 0.89 (0.46-1.71) | 0.72 |
| 613.1 | Inflammatory disease of breast | genitourinary | 16 | 0.87 (0.38-1.99) | 0.73 |
| 703.1 | Ingrowing nail | dermatologic | 16 | 1.13 (0.55-2.32) | 0.74 |
| 371.3 | Inflammation of eyelids | sense organs | 16 | 0.93 (0.59-1.46) | 0.74 |
| 550.5 | Ventral hernia | digestive | 16 | 0.94 (0.64-1.38) | 0.74 |
| 594 | Urinary calculus | genitourinary | 16 | 1.07 (0.72-1.57) | 0.74 |
| 819 | Skull and face fracture and other intercranial injury | injuries & poisonings | 16 | 0.91 (0.53-1.58) | 0.74 |
| 153 | Colorectal cancer | neoplasms | 16 | 0.93 (0.59-1.45) | 0.75 |
| 338 | Pain | symptoms | 16 | 1.14 (0.51-2.54) | 0.75 |
| 385 | Other disorders of middle ear and mastoid | sense organs | 16 | 0.88 (0.41-1.92) | 0.75 |
| 8.52 | Intestinal infection due to C. difficile | infectious diseases | 16 | 0.87 (0.36-2.09) | 0.75 |
| 709.2 | Sicca syndrome | dermatologic | 16 | 0.78 (0.16-3.70) | 0.76 |
| 501 | Pneumonitis due to inhalation of food or vomitus | respiratory | 16 | 1.16 (0.46-2.91) | 0.76 |
| 195 | Cancer, suspected or other | neoplasms | 16 | 0.97 (0.82-1.16) | 0.76 |
| 198.2 | Secondary malignancy of respiratory organs | neoplasms | 16 | 0.93 (0.58-1.50) | 0.76 |
| 426.2 | Atrioventricular [AV] block | circulatory system | 16 | 0.93 (0.57-1.51) | 0.77 |
| 727 | Other disorders of synovium, tendon, and bursa | musculoskeletal | 16 | 0.96 (0.74-1.25) | 0.77 |
| 823 | Torus fracture | injuries & poisonings | 16 | 0.92 (0.53-1.59) | 0.77 |
| 907 | Injuries to the nervous system | injuries & poisonings | 16 | 0.92 (0.49-1.70) | 0.78 |
| 771 | Musculoskeletal symptoms referable to limbs | symptoms | 16 | 0.96 (0.71-1.30) | 0.78 |
| 281 | Other deficiency anemia | hematopoietic | 16 | 0.91 (0.47-1.77) | 0.79 |
| 425 | Cardiomyopathy | circulatory system | 16 | 1.09 (0.58-2.06) | 0.79 |
| 681.2 | Cellulitis and abscess of face/neck | dermatologic | 16 | 1.14 (0.43-3.03) | 0.79 |
| 1009 | Injury, NOS | injuries & poisonings | 16 | 0.97 (0.75-1.24) | 0.79 |
| 599.2 | Retention of urine | genitourinary | 16 | 1.04 (0.79-1.37) | 0.80 |
| 250 | Diabetes mellitus | endocrine/metabolic | 16 | 1.05 (0.70-1.58) | 0.81 |
| 574 | Cholelithiasis and cholecystitis | digestive | 16 | 0.95 (0.64-1.42) | 0.81 |
| 701.2 | Scar conditions and fibrosis of skin | dermatologic | 16 | 0.94 (0.60-1.50) | 0.81 |
| 512.9 | Other dyspnea | respiratory | 16 | 1.09 (0.52-2.30) | 0.81 |
| 451.2 | Phlebitis and thrombophlebitis of lower extremities | circulatory system | 16 | 0.93 (0.53-1.64) | 0.81 |
| 430.2 | Intracerebral hemorrhage | circulatory system | 16 | 0.90 (0.39-2.11) | 0.82 |
| 292 | Neurological disorders | mental disorders | 16 | 0.96 (0.69-1.34) | 0.82 |
| 165.1 | Cancer of bronchus; lung | neoplasms | 16 | 0.94 (0.58-1.54) | 0.82 |
| 558 | Noninfectious gastroenteritis | digestive | 16 | 0.97 (0.73-1.28) | 0.82 |
| 289 | Other diseases of blood and blood-forming organs | hematopoietic | 16 | 1.06 (0.65-1.71) | 0.83 |
| 578.8 | Hemorrhage of rectum and anus | digestive | 16 | 1.02 (0.84-1.24) | 0.83 |
| 710.1 | Osteomyelitis | musculoskeletal | 16 | 0.91 (0.35-2.31) | 0.84 |
| 789 | Nausea and vomiting | symptoms | 16 | 0.97 (0.74-1.28) | 0.84 |
| 427.5 | Arrhythmia (cardiac) NOS | circulatory system | 16 | 0.93 (0.44-1.94) | 0.84 |
| 368.1 | Amblyopia | sense organs | 16 | 1.10 (0.42-2.89) | 0.84 |
| 210 | Benign neoplasm of lip, oral cavity, and pharynx | neoplasms | 16 | 0.93 (0.46-1.90) | 0.84 |
| 727.1 | Synovitis and tenosynovitis | musculoskeletal | 16 | 0.96 (0.62-1.47) | 0.85 |
| 540 | Appendiceal conditions | digestive | 16 | 0.96 (0.65-1.42) | 0.85 |
| 8.5 | Bacterial enteritis | infectious diseases | 16 | 1.04 (0.68-1.60) | 0.85 |
| 371 | Inflammation of the eye | sense organs | 16 | 0.96 (0.65-1.43) | 0.85 |
| 818 | Intracranial hemorrhage (injury) | injuries & poisonings | 16 | 0.91 (0.34-2.46) | 0.85 |
| 386.9 | Dizziness and giddiness (Light-headedness and vertigo) | sense organs | 16 | 0.97 (0.70-1.35) | 0.86 |
| 798.1 | Chronic fatigue syndrome | symptoms | 16 | 0.92 (0.37-2.30) | 0.86 |
| 741.2 | Stiffness of joint | musculoskeletal | 16 | 1.08 (0.44-2.66) | 0.86 |
| 578.9 | Hemorrhage of gastrointestinal tract | digestive | 16 | 1.03 (0.75-1.40) | 0.86 |
| 378 | Strabismus and other disorders of binocular eye movements | sense organs | 16 | 0.95 (0.53-1.71) | 0.87 |
| 214.1 | Lipoma of skin and subcutaneous tissue | neoplasms | 16 | 1.03 (0.74-1.43) | 0.87 |
| 520 | Disorders of tooth development | digestive | 16 | 1.04 (0.66-1.63) | 0.87 |
| 722.1 | Displacement of intervertebral disc | musculoskeletal | 16 | 1.08 (0.40-2.90) | 0.87 |
| 214 | Lipoma | neoplasms | 16 | 1.03 (0.69-1.56) | 0.88 |
| 560.4 | Other intestinal obstruction | digestive | 16 | 1.03 (0.70-1.52) | 0.88 |
| 526 | Diseases of the jaws | digestive | 16 | 0.95 (0.46-1.95) | 0.88 |
| 726.1 | Enthesopathy | musculoskeletal | 16 | 1.02 (0.81-1.28) | 0.89 |
| 571.5 | Other chronic nonalcoholic liver disease | digestive | 16 | 0.93 (0.34-2.51) | 0.89 |
| 717 | Polymyalgia Rheumatica | musculoskeletal | 16 | 0.95 (0.49-1.84) | 0.89 |
| 979 | Adverse drug events and drug allergies | injuries & poisonings | 16 | 0.94 (0.40-2.20) | 0.89 |
| 458.1 | Orthostatic hypotension | circulatory system | 16 | 1.04 (0.57-1.92) | 0.89 |
| 204 | Leukemia | neoplasms | 16 | 0.97 (0.56-1.67) | 0.90 |
| 807 | Fracture of ribs | injuries & poisonings | 16 | 0.95 (0.43-2.12) | 0.90 |
| 687.4 | Disturbance of skin sensation | dermatologic | 16 | 1.02 (0.68-1.55) | 0.91 |
| 479 | Other upper respiratory disease | respiratory | 16 | 0.98 (0.69-1.39) | 0.91 |
| 289.5 | Diseases of spleen | hematopoietic | 16 | 0.95 (0.35-2.54) | 0.92 |
| 520.2 | Disturbances in tooth eruption | digestive | 16 | 0.98 (0.62-1.55) | 0.92 |
| 560.1 | Paralytic ileus | digestive | 16 | 0.94 (0.25-3.55) | 0.93 |
| 165 | Cancer within the respiratory system | neoplasms | 16 | 1.02 (0.66-1.57) | 0.93 |
| 561 | Symptoms involving digestive system | digestive | 16 | 1.01 (0.84-1.21) | 0.93 |
| 195.1 | Malignant neoplasm, other | neoplasms | 16 | 0.99 (0.83-1.19) | 0.94 |
| 217.1 | Nevus, non-neoplastic | neoplasms | 16 | 1.04 (0.42-2.58) | 0.94 |
| 394.7 | Disease of tricuspid valve | circulatory system | 16 | 0.98 (0.49-1.94) | 0.94 |
| 300.12 | Agorophobia, social phobia, and panic disorder | mental disorders | 16 | 1.03 (0.44-2.39) | 0.94 |
| 260 | Protein-calorie malnutrition | endocrine/metabolic | 16 | 1.02 (0.52-2.03) | 0.95 |
| 575 | Other biliary tract disease | digestive | 16 | 0.99 (0.69-1.42) | 0.95 |
| 694.2 | Other dyschromia | dermatologic | 16 | 1.02 (0.46-2.27) | 0.95 |
| 573.7 | Abnormal results of function study of liver | digestive | 16 | 0.99 (0.67-1.45) | 0.95 |
| 599.4 | Urinary incontinence | genitourinary | 16 | 1.01 (0.72-1.42) | 0.96 |
| 706.2 | Sebaceous cyst | dermatologic | 16 | 0.99 (0.78-1.27) | 0.96 |
| 706 | Diseases of sebaceous glands | dermatologic | 16 | 0.99 (0.78-1.26) | 0.96 |
| 172.3 | Carcinoma in situ of skin | neoplasms | 16 | 1.02 (0.43-2.43) | 0.96 |
| 241.2 | Nontoxic multinodular goiter | endocrine/metabolic | 16 | 1.02 (0.43-2.43) | 0.96 |
| 281.11 | Pernicious anemia | hematopoietic | 16 | 1.02 (0.43-2.43) | 0.96 |
| 686.3 | Pilonidal cyst | dermatologic | 16 | 1.02 (0.40-2.57) | 0.97 |
| 594.2 | Calculus of lower urinary tract | genitourinary | 16 | 0.98 (0.32-3.01) | 0.98 |
| 574.1 | Cholelithiasis | digestive | 16 | 1.01 (0.65-1.56) | 0.98 |
| 276.5 | Hypovolemia | endocrine/metabolic | 16 | 1.01 (0.66-1.53) | 0.98 |
| 286 | Coagulation defects | hematopoietic | 16 | 0.99 (0.48-2.06) | 0.98 |
| 559 | Ileostomy status | digestive | 16 | 0.99 (0.57-1.72) | 0.98 |
| 771.1 | Swelling of limb | symptoms | 16 | 1.00 (0.72-1.38) | 0.98 |
| 619.1 | Noninflammatory disorders of ovary, fallopian tube, and broad ligament | genitourinary | 16 | 1.01 (0.44-2.31) | 0.98 |
| 153.3 | Malignant neoplasm of rectum, rectosigmoid junction, and anus | neoplasms | 16 | 1.00 (0.61-1.63) | 0.99 |
| 215 | Other benign neoplasm of connective and other soft tissue | neoplasms | 16 | 0.99 (0.51-1.94) | 0.99 |
| 295.1 | Schizophrenia | mental disorders | 16 | 1.01 (0.39-2.56) | 0.99 |
| 361 | Retinal detachments and defects | sense organs | 16 | 1.00 (0.67-1.49) | 0.99 |
| 785 | Abdominal pain | symptoms | 16 | 1.00 (0.86-1.16) | 0.99 |
| 292.4 | Altered mental status | mental disorders | 16 | 1.00 (0.62-1.61) | 1.00 |
| 502 | Postinflammatory pulmonary fibrosis | respiratory | 16 | 1.00 (0.47-2.12) | 1.00 |
| 191 | Manlignant and unknown neoplasms of brain and nervous system | neoplasms | 16 | 1.00 (0.42-2.39) | 1.00 |
| 384.4 | Perforation of tympanic membrane | sense organs | 16 | 1.00 (0.50-2.00) | 1.00 |

Odds ratios (ORs) with their 95% confidence intervals (CIs) represent the association estimates with the risks of non-breast cancer of per 1-SD increase of acetate, respectively.

Significant threshold in stage 2 was set at set at *P*<0.05/1358=3.68×10^-5^, which was corrected for multiple comparisons using the Bonferroni method (0.05/1358 [2 identified breast cancer metabolites in stage 1×679 diseases]).

Abbreviations: Phe-MR, phenome-wide Mendelian randomization; SNPs, single nucleotide polymorphisms.

**Table S8. Sensitivity analyses for all significant results** **in the inverse-variance weighted Phe-MR analysis.**

| **Metabolite** | **PheCode** | **Outcome** | **Disease chapter** | **SNPs** | **Weighted median** | | **MR-RAPS** | | **MR-Egger Intercept** |
| --- | --- | --- | --- | --- | --- | --- | --- | --- | --- |
|  |  |  |  |  | **OR (95% CI)** | ***P* value** | **OR (95% CI)** | ***P* value** | ***P* value** |
| **HDL-C** | | | | | | | | | |
|  | 401.1 | Essential hypertension | circulatory system | 272 | 0.93(0.90-0.97) | 2.88×10^-4^ | 0.90(0.88-0.91) | 4.76×10^-25^ | 0.01 |
|  | 250.2 | Type 2 diabetes | endocrine/metabolic | 272 | 0.91(0.85-0.98) | 0.01 | 0.83(0.80-0.86) | 2.91×10^-23^ | 1.05×10^-4^ |
|  | 250 | Diabetes mellitus | endocrine/metabolic | 272 | 0.95(0.88-1.01) | 0.11 | 0.84(0.81-0.87) | 3.50×10^-21^ | 4.83×10^-5^ |
|  | 411.2 | Myocardial infarction | circulatory system | 272 | 0.84(0.77-0.92) | 1.12×10^-4^ | 0.80(0.77-0.84) | 1.40×10^-21^ | 0.06 |
|  | 411.3 | Angina pectoris | circulatory system | 272 | 0.88(0.81-0.94) | 3.32×10^-4^ | 0.83(0.79-0.86) | 1.99×10^-21^ | 0.01 |
|  | 411.4 | Coronary atherosclerosis | circulatory system | 272 | 0.85(0.79-0.91) | 3.96×10^-6^ | 0.80(0.77-0.83) | 2.45×10^-33^ | 0.07 |
|  | 411.8 | Other chronic ischemic heart disease, unspecified | circulatory system | 272 | 0.92(0.86-1.00) | 0.04 | 0.86(0.82-0.89) | 9.30×10^-14^ | 0.03 |
|  | 411 | Ischemic Heart Disease | circulatory system | 272 | 0.86(0.81-0.91) | 3.72×10^-7^ | 0.83(0.80-0.85) | 2.66×10^-36^ | 0.02 |
|  | 426 | Cardiac conduction disorders | circulatory system | 272 | 0.86(0.78-0.95) | 3.94×10^-3^ | 0.83(0.78-0.88) | 1.45×10^-10^ | 0.32 |
|  | 585 | Renal failure | genitourinary | 272 | 0.92(0.83-1.01) | 0.08 | 0.87(0.83-0.92) | 3.50×10^-6^ | 0.07 |
|  | 599.4 | Urinary incontinence | genitourinary | 272 | 0.90(0.82-0.99) | 0.02 | 0.89(0.84-0.93) | 4.09×10^-6^ | 0.47 |
|  | 1010 | Other tests | symptoms | 272 | 0.79(0.81-0.89) | 6.13×10^-5^ | 0.88(0.82-0.93) | 2.35×10^-5^ | 0.74 |
|  | 225.1 | Benign neoplasm of brain, cranial nerves, meninges | neoplasms | 272 | 1.29(0.97-1.73) | 0.08 | 1.47(1.24-1.74) | 8.84×10^-6^ | 0.86 |
|  | 440.2 | Atherosclerosis of the extremities | circulatory system | 272 | 0.58(0.43-0.79) | 4.63×10^-4^ | 0.61(0.52-0.73) | 8.52×10^-9^ | 0.64 |
|  | 225 | Benign neoplasm of brain and other parts of nervous system | neoplasms | 272 | 1.34(1.00-1.80) | 0.05 | 1.45(1.23-1.71) | 7.59×10^-6^ | 0.62 |
|  | 381.11 | Suppurative and unspecified otitis media | sense organs | 272 | 0.68(0.51-0.89) | 5.51×10^-3^ | 0.69(0.59-0.81) | 5.62×10^-6^ | 0.24 |
|  | 440 | Atherosclerosis | circulatory system | 272 | 0.72(0.58-0.90) | 4.15×10^-3^ | 0.71(0.62-0.81) | 1.80×10^-7^ | 0.99 |
|  | 442.1 | Aortic aneurysm | circulatory system | 272 | 0.72(0.57-0.91) | 6.16×10^-3^ | 0.71(0.62-0.80) | 8.80×10^-8^ | 0.58 |
|  | 442.11 | Abdominal aortic aneurysm | circulatory system | 272 | 0.52(0.39-0.69) | 7.56×10^-6^ | 0.61(0.52-0.72) | 1.01×10^-9^ | 0.52 |
|  | 444 | Arterial embolism and thrombosis | circulatory system | 272 | 0.52(0.39-0.70) | 1.12×10^-5^ | 0.59(0.50-0.69) | 2.70×10^-11^ | 0.72 |
|  | 447 | Other disorders of arteries and arterioles | circulatory system | 272 | 0.75(0.59-0.95) | 0.02 | 0.76(0.66-0.86) | 2.25×10^-5^ | 0.74 |
|  | 1001 | Foreign body injury | injuries & poisonings | 272 | 0.59(0.46-0.75) | 3.01×10^-5^ | 0.65(0.56-0.74) | 2.10×10^-10^ | 0.19 |
|  | 555 | Inflammatory bowel disease and other gastroenteritis and colitis | digestive | 272 | 0.85(0.75-0.97) | 0.01 | 0.83(0.77-0.89) | 4.00×10^-7^ | 0.33 |
|  | 204 | Leukemia | neoplasms | 272 | 1.33(1.09-1.62) | 4.87×10^-3^ | 1.35(1.20-1.52) | 3.18×10^-7^ | 0.42 |
|  | 381 | Otitis media and Eustachian tube disorders | sense organs | 272 | 0.78(0.65-0.94) | 7.66×10^-3^ | 0.80(0.72-0.88) | 1.16×10^-5^ | 0.75 |
|  | 381.1 | Otitis media | sense organs | 272 | 0.77(0.63-0.93) | 8.30×10^-3^ | 0.76(0.68-0.85) | 1.80×10^-6^ | 0.54 |
|  | 443.9 | Peripheral vascular disease, unspecified | circulatory system | 272 | 0.80(0.67-0.94) | 6.26×10^-3^ | 0.79(0.72-0.87) | 1.48×10^-6^ | 0.20 |
|  | 572 | Ascites (non- malignant) | digestive | 272 | 0.76(0.62-0.92) | 6.48×10^-3^ | 0.75(0.66-0.84) | 1.92×10^-6^ | 0.14 |
|  | 411.1 | Unstable angina (intermediate coronary syndrome) | circulatory system | 272 | 0.81(0.72-0.92) | 8.05×10^-4^ | 0.80(0.75-0.86) | 1.0×10^-10^ | 0.20 |
|  | 444.1 | Arterial embolism and thrombosis of lower extremity artery | circulatory system | 272 | 0.53(0.38-0.75) | 3.41×10^-4^ | 0.58(0.48-0.71) | 1.32×10^-7^ | 0.22 |
|  | 850 | Hemorrhage or hematoma complicating a procedure | injuries & poisonings | 272 | 0.87(0.77-0.97) | 0.02 | 0.85(0.79-0.90) | 4.05×10^-7^ | 0.11 |
|  | 681.5 | Cellulitis and abscess of leg, except foot | dermatologic | 272 | 0.87(0.78-0.97) | 0.02 | 0.87(0.81-0.93) | 1.42×10^-5^ | 0.98 |
| **Acetate** | | | | | | | | | |
|  | 477 | Epistaxis or throat hemorrhage | respiratory | 16 | 0.28(0.15-0.55) | 2.08×10^-4^ | 0.23(0.14-0.37) | 1.19×10^-9^ | 0.08 |
|  | 318 | Tobacco use disorder | mental disorders | 16 | 0.58(0.45-0.75) | 2.84×10^-5^ | 0.60(0.50-0.72) | 9.91×10^-9^ | 0.92 |
|  | 394.3 | Aortic valve disease | circulatory system | 16 | 0.30(0.12-0.75) | 0.01 | 0.19(0.10-0.36) | 4.33×10^-7^ | 0.79 |
|  | 433 | Cerebrovascular disease | circulatory system | 16 | 0.67(0.48-0.95) | 0.02 | 0.54(0.42-0.69) | 1.22×10^-6^ | 0.03 |
|  | 365 | Glaucoma | sense organs | 16 | 1.93(1.17-3.18) | 0.01 | 2.33(1.64-3.30) | 2.53×10^-6^ | 0.83 |
|  | 411.1 | Unstable angina (intermediate coronary syndrome) | circulatory system | 16 | 0.42(0.27-0.65) | 9.25×10^-5^ | 0.46(0.33-0.64) | 4.12×10^-6^ | 0.19 |
|  | 854 | Complications of cardiac/vascular device, implant, and graft | injuries & poisonings | 16 | 0.26(0.13-0.54) | 3.14×10^-4^ | 0.27(0.16-0.47) | 2.76×10^-6^ | 0.08 |
|  | 960 | Poisoning by antibiotics | injuries & poisonings | 16 | 0.70(0.55-0.90) | 4.67×10^-3^ | 0.67(0.56-0.80) | 1.17×10^-5^ | 0.09 |
|  | 411.8 | Other chronic ischemic heart disease, unspecified | circulatory system | 16 | 0.64(0.49-0.84) | 1.17×10^-3^ | 0.54(0.44-0.66) | 2.74×10^-9^ | 0.09 |
|  | 960.2 | Allergy/adverse effect of penicillin | injuries & poisonings | 16 | 0.68(0.52-0.89) | 4.89×10^-3^ | 0.66(0.55-0.80) | 2.06×10^-5^ | 0.14 |
|  | 480 | Pneumonia | respiratory | 16 | 0.64(0.47-0.87) | 4.51×10^-3^ | 0.62(0.49-0.78) | 5.00×10^-5^ | 0.62 |

Odds ratios (ORs) with their 95% confidence intervals (CIs) represent the association estimates with the risks of non-breast cancer of per 1-SD increase of HDL-C and acetate, respectively.

Significant threshold in stage 2 was set at set at *P*<0.05/1358=3.68×10^-5^, which was corrected for multiple comparisons using the Bonferroni method (0.05/1358 [2 identified breast cancer metabolites in stage 1×679 diseases]).

Abbreviations: MR-RAPS, Mendelian randomization robust adjusted profile score; Phe-MR, phenome-wide Mendelian randomization; SNPs, single nucleotide polymorphisms.

**Table S9. Summary of significant Phe-MR findings representing on-target side-effect of metabolites supplementation.**

| **Metabolite** | **Number of significant disease associations** | **Number of beneficial/deleterious side effects (%)** | **Top ICD-9 disease chapters (%)** | **Most significant disease (beneficial or deleterious)** |
| --- | --- | --- | --- | --- |
| HDL-C | 4 | 0/4 (100) | Circulatory system (75);  injuries & poisonings (25) | Coronary atherosclerosis |
| Acetate | 1 | 0/1 (100) | Mental disorders (100) | Tobacco use disorder |
| **Total** | **5** | **0/5** | **Circulatory system (60)** | - |

Abbreviations: HDL-C, High density liptein cholesterol; ICD, International Classification of Diseases; Phe-MR, phenome-wide Mendelian, randomization.
